# A Geometric Clustering Tool (AGCT) to robustly unravel the inner cluster structures of time-series gene expressions

**DOI:** 10.1371/journal.pone.0233755

**Published:** 2020-07-06

**Authors:** Richard Nock, Natalia Polouliakh, Frank Nielsen, Keigo Oka, Carlin R. Connell, Cedric Heimhofer, Kazuhiro Shibanai, Samik Ghosh, Ken-ichi Aisaki, Satoshi Kitajima, Jun Kanno, Taketo Akama, Hiroaki Kitano

**Affiliations:** 1 NICTA/Data61 & the Australian National University, Alexandria, Australia; 2 Sony Computer Science Laboratories Inc., Tokyo, Japan; 3 Department of Ophthalmology and Visual Science, Yokohama City University, Yokohama, Japan; 4 Systems Biology Institute, Tokyo, Japan; 5 The University of Tokyo, Tokyo, Japan; 6 Department of Computer Science, Queensland University of Technology, Brisbane, Australia; 7 Department of Computer Science, ETH Zurich, Zurich, Switzerland; 8 Department of Computer Science, School of Computing, Tokyo Institute of Technology, Tokyo, Japan; 9 National Institute of Health Science, Kawasaki, Japan; 10 Japan Bioassay Research Center, Japan Organization of Occupational Health and Safety, Hadano, Japan; University of Texas Health Science Center at San Antonio, UNITED STATES

## Abstract

Systems biology aims at holistically understanding the complexity of biological systems. In particular, nowadays with the broad availability of gene expression measurements, systems biology challenges the deciphering of the genetic cell machinery from them. In order to help researchers, reverse engineer the genetic cell machinery from these noisy datasets, interactive exploratory clustering methods, pipelines and gene clustering tools have to be specifically developed. Prior methods/tools for time series data, however, do not have the following four major ingredients in analytic and methodological view point: (i) principled time-series feature extraction methods, (ii) variety of manifold learning methods for capturing high-level view of the dataset, (iii) high-end automatic structure extraction, and (iv) friendliness to the biological user community. With a view to meet the requirements, we present AGCT (A Geometric Clustering Tool), a software package used to unravel the complex architecture of large-scale, non-necessarily synchronized time-series gene expression data. AGCT capture signals on exhaustive wavelet expansions of the data, which are then embedded on a low-dimensional non-linear map using manifold learning algorithms, where geometric proximity captures potential interactions. Post-processing techniques, including hard and soft information geometric clustering algorithms, facilitate the summarizing of the complete map as a smaller number of principal factors which can then be formally identified using embedded statistical inference techniques. Three-dimension interactive visualization and scenario recording over the processing helps to reproduce data analysis results without additional time. Analysis of the whole-cell Yeast Metabolic Cycle (YMC) moreover, Yeast Cell Cycle (YCC) datasets demonstrate AGCT's ability to accurately dissect all stages of metabolism and the cell cycle progression, independently of the time course and the number of patterns related to the signal. Analysis of Pentachlorophenol iduced dataset demonstrat how AGCT dissects data to identify two networks: Interferon signaling and NRF2-signaling networks.

## Introduction

A systematic analysis of massive, high-throughput biological data must be based on sound mathematics and flexible control to handle the stochasticity, plasticity, and modularity seen in genetic applications. An essential step in the analysis of the gene expression is grouping genes by the similarity of their expression profile, representing gene expression changes over time.

There is wealthy literature on cluster analysis going back over three decades [1, 2], among those are famous techniques as k-means [3], hierarchical clustering (HC) [4], self-organizing maps (SOM) [5] or simulated annealing [6].

Based on these methods, many software tools applicable to the time-series data have been created; and examples are Expander [7], STEM [8], TimeCluster [9] or GenePattern [10]. Such methods are excellent at representing details or comparatively small or medium size structures, but they may fall short in providing a high-level view of the data set. In part, this is because they rely on pairwise distance computations, and the distance function is often chosen *a priori* among a set of candidates such as the Euclidean, Manhattan, or more generally Minkowski norm-based distances. Setting the distance a priori neglects the intrinsic underlying geometry of data [11, 12]. When processing large data sets, such as those from eukaryote cell gene expression, it is useful if clustering is accompanied by methods that fit high-level structures by embedding the geometry in a low-dimensional interpretable space, as with linear dimensionality reduction (e.g., principal component analysis [13]).

Recent non-linear dimensionality reduction methods like the t-distributed Stochastic Neighbor Embedding (t-SNE) [14] improved the visualization of high-dimensional non-affine data manifolds considerably, and have also shown promising results for gene expression analysis [15]. However, t-SNE requires both the manual definition of low-level features from data sets and the prescription of an ad hoc distance function. Moreover, the t-SNE embedding is often inspected and evaluated by users in 2D space and lacks interactivity for exploring the various potential clusterings as well as in other nonlinear dimensionality reduction tool Isomap [16].

We present a cross-platform Java™ tool, AGCT (A Geometric Clustering Tool) that examines the dynamics of gene expression by principled time-series feature extraction method, state-of-the-art low-dimensional manifold learning techniques coupled with robust methods for clustering, data visualization, and statistical validation. To demonstrate the analysis workflows, we selected three datasets: the Yeast Metabolic Cycle (YMC) experiment [17, 18], the Yeast Cell Cycle (YCC) [19, 20] and dioxin supplemented mouse liver dataset [21].

Among diverse methods for each workflow provided by AGCT (see Sec.2), we employed the following methods. Time-series datasets are encoded with n = 64 on Haar wavelets, a cosine similarity matrix is calculated followed by sparsification. The matrix is normalized as a transition matrix of Markov chain, which is then eigen decomposed to compute the manifold coordinates. On the manifolds, we performed clustering by NNMF, Bregman k-means, and further analysed with Delaunay triangulation on Yeast data. We also applied Affinity propagation method followed by Delaunay triangulation extraction on mouse data. The results showed that a careful blend of tailored representation and manifold learning techniques results in a very accurate bird’s-eye view of the YCC. It also provides a cluster alignment with the YMC, which, prior to AGCT, was mostly out of reach by analysis techniques used to date. Beyond the yeast cycles, we make the striking observation that the manifold successfully captures, in low dimensions, a meaningful organization of the intertwined structures of large-scale multidimensional data.

Table 1 summarizes the key novelty of AGCT compared with other tools. Detailed comparisons are described later in Table 2 and the corresponding section.

**Table 1:** The key novelty of AGCT.

Name of Tool	principle time-series feature extraction method	manifold-learning method for capturing high-level view	high-end automatic structure extraction	friendliness to the biological user community
AGCT	yes	yes	yes	yes
EXPANDER	no	no	yes	partially yes
STEM	no	no	yes	yes
Scikit	no	yes	yes	yes
Visgene2.0	no	yes	partially yes	partially yes

**Table 2:** (a). Characteristics of tools similar in features with AGCT and selected for testing. Methodoly, features, and presense/absence of graphical user interface and Gene ontology analyses are listed. (b) Validation of clustering tools on 3656 probes dataset. (c) Validation of clustering tools on 9335 probes dataset.

A					
Tool	Single Tool/Benchmark	Clustering method	Feature computing/ Manifold learning	GUI	GO
AGCT	Benchmark	Bregman K-means (K = 3, K = 7), Affinity propagation	Haar wavelet/YES	Yes	Yes
EXPANDER	Benchmark	CLICK	No/No	Yes	Yes
EXPANDER	Benchmark	SOM (w = 93, h = 93) (w = 30, h = 24)	No/No	Yes	Yes
EXPANDER	Benchmark	SAMBA (Biclustring)	No/No	Yes	Yes
STEM	Benchmark	STEM (m = 3, m = 7)	No/No	Yes	Yes
STEM	Benchmark	STEM (m = 50)	No/No	Yes	Yes
Scikit	Single	t-SNE, K-means (K = 3, K = 7), time-series data	No/Yes	Yes	No
Scikit	Single	t-SNE, K-means (K = 3, K = 7), manifold data	No/Yes	Yes	No
Visgene 2.0	Single	t-SNE, K-means (K = 4)	No/Yes	No	Yes
Scikit	Single	Isomap, K-means (K = 7), time-series data	No/Yes	No	No
Scikit	Single	Isomap, K-means (K = 7), manifold data	No/Yes	No	No
B					
	**3656** probes (with 136 sentinels: 55 Ox, 40 R/B, 41 R/C Tu et al.)				
	#cl(def)	Executable	Sent Executed	Sent Assign Correct	Sent Cluster Collision
AGCT	7	100.0	100.0	90.0	0.0
EXPANDER (CLICK)	11	100.0	100.0	100.0	0.0
EXPANDER (SOM)	36	100.0	100.0	10.0	10.0
EXPANDER (SAMBA)	21	90.0	40.0	60.0	0.0
STEM m = 7	7	90.0	100.0	80.0	10.0
STEM m = 50	50	80.0	100.0	90.0	0.0
Scikit t-SNE non-manifold	7	100.0	100.0	50.0	40.0
Scikit t-SNE manifold	7	100.0	100.0	80.0	0.0
Visgene 2.0 t-SNE	4	100.0	100.0	90.0	0.0
Scikit Isomap non-manifold	7	100.0	100.0	40.0	20.0
Scikit Isomap manifold	7	100.0	100.0	30.0	60.0
C					
** **	**9335** probes (with 146 sentinels: 61 Ox, 40 R/B, 45 R/C Tu et al.)				
** **	#cl(def)	Executable	Sent Executed	Sent Assign Correct	Sent Cluster Collision
AGCT	3	100.0	100.0	100.0	0.0
EXPANDER (CLICK)	1	99.0	100.0	100.0	60.0
EXPANDER (SOM)	46	100.0	100.0	10.0	0.0
EXPANDER (SAMBA)	26	90.0	50.0	70.0	20.0
STEM m = 3	3	30.0	20.0	10.0	0.0
STEM m = 50	50	50.0	10.0	70.0	0.0
Scikit t-SNE non-manifold	3	10.0	10.0	80.0	50.0
Scikit t-SNE manifold	3	10.0	10.0	80.0	50.0
Visgene 2.0 t-SNE	-	-	-	-	-
Scikit Isomap non-manifold	-	-	-	-	-
Scikit Isomap manifold	-	-	-	-	-

Table titles are as following: #cl(def)—number of clusters under default, "Executable"—number of probes (%) a tool able to use, "Sent Executable" -number of sentinels (selected meaningful periodic Tu et al.) probes a tool includes in analysis, "Sent Assigned Correct."—a number of sentinels assigned correctly into the same clusters, as co-reculated; Sent Cluster Collision—number of misallocation of co-regulated sentinels to different clusters. For example, CLICK assigning 9335 dataset makes one cluster. Clustering 61 Oxidative, 40 Reactive Building, and 45 Reactive Charging sentinels probes together. Given that there are 145 sentinels and 84 are sharing the same cluster, giving it a value of 60.0.

## Results

### AGCT analysis tool

We present AGCT (A Geometric Clustering Tool), a software package used to unravel the complex architecture of large-scale, non-necessarily synchronized time-series gene expression data. In the context of existing tools the reason why the contribution by AGCT is significant stems more from three independent considerations on what a useful clustering tool for gene profile clustering should satisfy at large: (A) facilitate the exploratory process (which clustering is) with easy reproducibility, (B) provide a large panel of choice of methods for clustering, (C) make sure that these methods operate on a good representation of the data manifold. Consideration (C) is the one that has been considerably developed in machine learning over decades, and it is arguably most important to have accurate clustering results. In AGCT, the numerous techniques including crafting the manifold coordinates are the cornerstone that links the coordinate description (wavelets) to the clustering algorithms, for most accurate techniques.

For flexibility, each of the three main steps in AGCT has several different, compelling alternatives. To compute the similarity between two genes, we first process each gene time series into wavelets. Genes and wavelet features can be post-processed using prototype and feature selection methods (see Supplementary Information 2.3) that help address memory constraints or retain the most relevant information.

Then, we can compute between-gene similarities for the remaining genes using the remaining features. One example is the cosine similarity, which computes the cosine of the angle between the two gene representations and normalizes the result in the interval [0,1], where 1 means the angle is zero, indicating maximal similarity.

After all the similarities are computed, AGCT can learn the low-dimensional manifold using only the similarities. The output of this step is a new coordinate system for genes, which is typically low dimensional and nonlinear (unlike principal component analysis). This coordinate system tends to bring together sets of genes that are strongly similar and separates less similar genes.

As manifold learning algorithms, AGCT implements state-of-the-art techniques that include Markov chains, graph Laplacian, Bethe Hessian, and doubly stochastic matrix modeling. One key statistic is obtained from this second step: the local dimensions of the variety. This value is a pointwise estimator, for each gene, of its local number of "principal axes." The smaller this number, the more likely that the correlations between the time series result from meaningful interactions. Thus, a manifold learned from random data would have very high local dimensions. The last step is the extraction of the structure from these coordinate maps using powerful unsupervised algorithms. The main algorithms are clustering algorithms, including state-of-the-art affinity propagation, Bregman k-means++ clustering, expectation-maximization clustering, non-negative matrix factorization, complete positive factorization, and hierarchical clustering. A filtered Delaunay triangulation is also available, which represents and highlights all significant correlations between neighboring genes using colored edges. Because of a vast number of algorithms, a simple command-line language in AGCT allows users to run batches of these algorithms with a large number of parameters.

**Fig 1:**
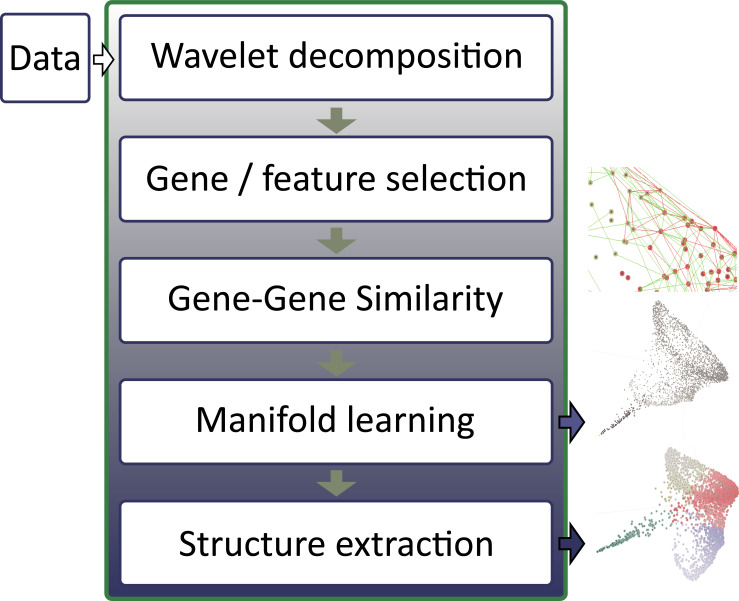
Analytic workflow in AGCT software.

Then, a search tool and graphical display allow users to pinpoint the most interesting clustering results for analysis. Since the complete workflow from the time series to post-processing is complex, a scenario recording and replaying tool (avoids re-computation and facilitates reproducibility in the analysis. Analysis workflow is depicted in Fig 1, and computational steps and techniques implemented in AGCT are described in S1 File and S1–S2 Tables of S1 File.

### Yeast metabolic cell cycle analysis

#### YMC whole-cell dataset, ground truth dataset, and sentinels

In an experiment using data from Tu et al. [17] (9,334 probes, 36 time points in three repetitive cycles, 300 minutes), our initial challenge was to determine if AGCT is sensitive enough to build a biologically reasonable architecture from a data set with a substantial number of irrelevant patterns, i.e., two-third (considering that only about a third are periodic). Periodic probes which number is 3,656 were experimentally identified in Tu et al., and used further as a “golden truth” for testing t-sne, manifold learning method in Bushati et al., [22] work. Ground truth dataset includes probes whose expression was periodic and exceeded 1.7 fold change. It comprises three groups of genes, i.e., clusters, named as Oxidative cluster (OX), induced by oxygen consumption; reductive-building cluster (R/B) acting when mitochondria respiration is needed in the protein synthesis stage and reductive-charging (R/C) which is active in non-respiratory mitotic phase. These three clusters include top periodically fluctuating gene probes, i.e., sentinels and represented by 55, 40, and 44 probes, respectively. The whole dataset of 9,334 probes includes 61, 40, and 44 probes, respectively. Those sentinels serve orienteers for accurate clustering if those sentinels are allocated together in the same cluster nevertheless of the noise size, which is two/third of data in the case of whole cell size (9,334 probes).

#### Analysis of Yeast Metabolic Cycles

AGCT uses the whole dataset (9,334 probes) to draw biologically meaningful conclusions basing on unsupervised learning clustering analysis solely. In the course of analysis, AGCT uses a local dimension score metric applied to the biological data for the first time to assess the role of each gene within the network in terms of its connectivity to other genes.

**Fig 2:**
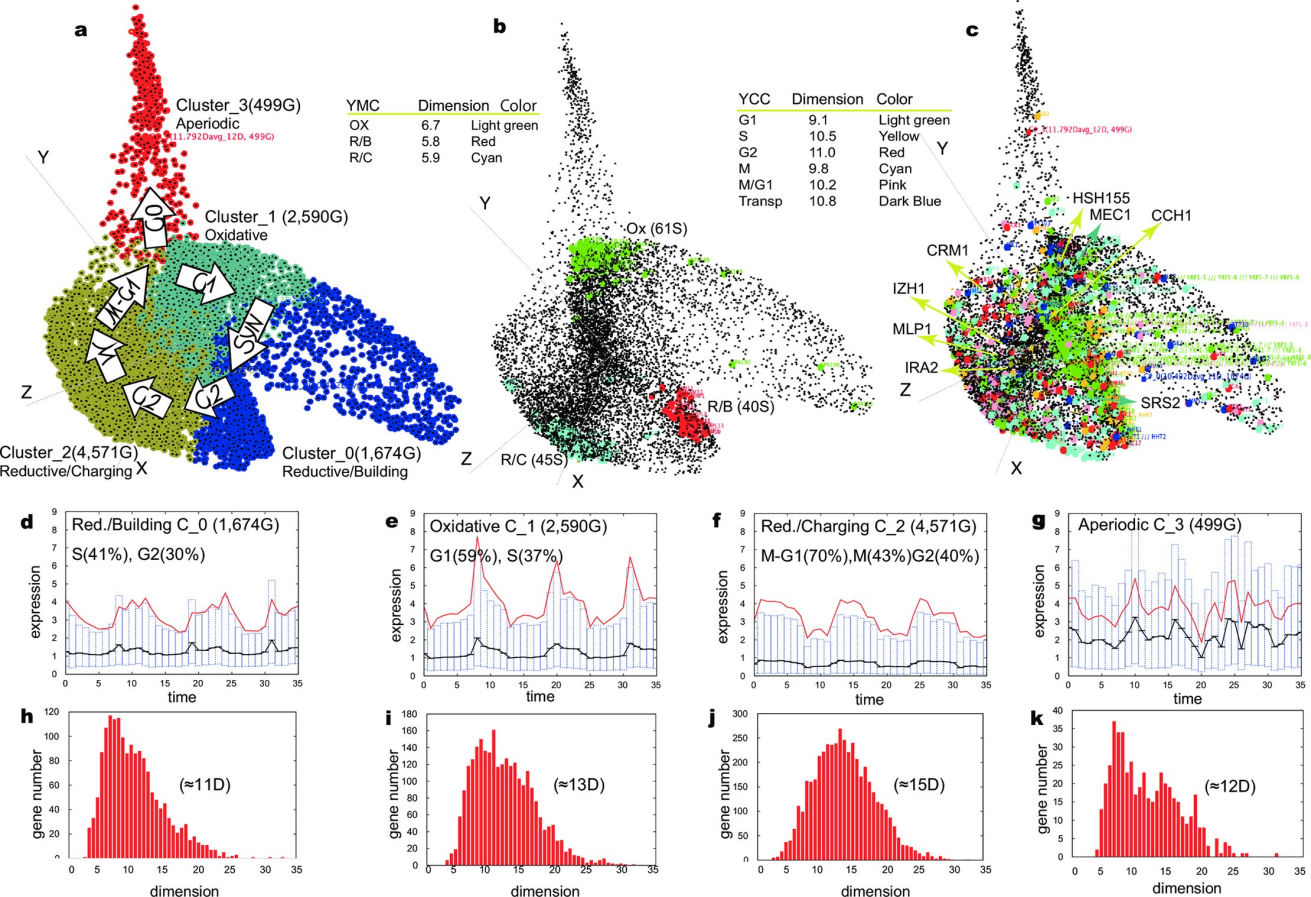
Clusters for the Yeast Metabolic Cycle (YMC) dataset (9,334G, 36 time points, 300 minutes): (a) Metabolic clusters aligned with the cell cycle. (b) YMC sentinel genes with average local dimension for each YMC phase in the table near the manifold. (c) YCC sentinels depicted by colored balls with a mean dimension for each YCC phase in the table near the manifold. Top Barycentre genes at a high local dimension are shown with respective dimensions in brackets and are pointed to with lines. (d)–(g) Cluster expression profiles, with candlesticks (solid red: average values; solid black: median values). Boxes indicate the Q25, Q50 (black), and Q75 quantiles. The x-axis shows time point tags, and Y-axis shows gene expression. (h)–(k) Dimension histograms of local dimensions (X-axes) and the number of genes (Y-axes) for respective clusters in (d)–(g). Local dimension scale of X-axes in (d)–(g) fits the scale of ambient space (X-axes) in (h)–(k).

To 9,334 probes we applied the Bregman k-means with five consecutive restarts within a range of 3≤k≤15, and obtained the best trade-off between the number of clusters and the data fitting for four clusters as shown in Fig 2 and S1A Fig. Four clusters obtained on AGCT contained yeast metabolic cycle sentinel groups experimentally identified in a previous Tu et al., study [17], as shown in Fig 2A and 2B: (1) Cluster 2 (4,571 genes (G) with 45 reductive-charging (R/C) sentinels; GO Tag for biological process: ribosome biogenesis 1.34E-74, mitochondrial translation 3.75E-62, rRNA processing 2.57E-35); (2) Cluster 1 (2,590 G, 61 oxidative (OX) sentinels; GO Tag for biological process: DNA recombination ∞, Transposition ∞, Proteolysis ∞); (3) Cluster 0 (1,674 G with 40 reductive-building (R/B) sentinels; GO Tag for biological process: translation 3.09E-53, ribosome biogenesis 1.32E-45, viral procapsid maturation 1.62E-28); and (4) *Aperiodic* Cluster 3 (499 G, GO Tag for biological process: translation 1.03E-60, DNA integration 1.35E-19, transposition 4.23E-19). S3 Table represents top scoring Gene Ontology terms in four clusters. Based on the clear separation of Yeast Metabolic Cycle sentinels into three clusters confirmed formerly in Tu et al., experiment [17], as shown in Fig 2B, the clusters with sentinels were named Reductive/Charging (R/C), Oxidative (OX), or Reductive/Building (R/B). The YMC sentinel details identified in the clusters are shown in S1a–1c Data. Cross-validation using non-negative matrix factorization (NNMF) [23, 24] clustering confirmed the existence of only two sub-spaces: the first with three metabolic clusters and the second with an *Aperiodic* cluster as shown in S2 Fig. Inspection of clusters obtained on AGCT also was done using experimentally verified [20] Yeast Cell Cycle sentinel genes representing cell cycle phases and the results demonstrated the meaningful overlap of cell cycle phases with the three metabolic clusters shown in Fig 2C.

The Reductive/Charging Cluster 2 is the largest cluster with the largest local dimensions (≈15D) (Fig 2F and 2J) and it fits the mitotic phase of YCC, where fatty acid oxidation under anaerobic respiration produces acetyl-CoA units as an energy source for cell division. The Reductive/Charging cluster (≈15D) (Fig 2F and 2J) and Oxidative cluster (≈13D) (Fig 2E and 2I)—tagged in the G1, M-G1, and M phases—comprise a *Barycentre* of the data. The *Barycentre* includes genes in a large local dimension, activating the **selection switch** between (1) *YCC progression state*, the (2) *stationary dormant state G0* depending on the presence/absence of an energy supply, in this case, glucose and *mating state* [25, 26]. Having a role of switch Barycentre large dimension genes act as a hyperplane classifier that conducts the behaviour of genes in smaller dimensions [27]. The top large (Fig 2C) dimension classifiers for the Yeast Metabolic Cycle are _35.8_*CRM1*^R/C(2)^ (exportin), _32.6_*MEC1*^OX(1)^ (genome integrity checkpoint), _30.4_*MLP1*^R/C(2)^ (telomere length control), _29.4_*IRA2*^R/C(2)^ (cAMP-dependent nutrient-limiting conditions), _26.6_*USO1*^OX(1)^ (transport from ER to Golgi), _26.5_*IZH4* ^M/G,R/C(2)^ (zinc metabolism), _26.5_*GCN1*^R/C(2)^ (regulator of the Gcn2p kinase activity), _25.3_*SRS2*^R/C(1)^ (commitment to meiotic recombination), _21.1_*CCH1*^R/C(2)^ (calcium channel to some environmental stresses and mating pheromones), and _23.7_*HSH155*^R/C(2)^ (genome integrity checkpoint protein) (S1i Data). (The left lower cases near the gene name show the average dimension computed for a gene; the upper right cases show the cluster and annotation by metabolic or cell cycle when available.)They belong to the Reductive/Charging Cluster 2 and Oxidative Cluster 1 and are main cellular switches to the global environmental signals. For example, large dimension Barycenter genes regulate transition from M-phase to G1-phase of Yeast Cell Cycle as it is shown in S3A, S3B, S3C Fig. Yeast Cell Cycle genes are in a smaller dimension (Fig 2C), and Yeast Metabolic Cycle genes are in an even smaller dimension than YCC genes (Fig 2B). This indicates a small number of free (unknown) parameters (CIT2 [28]) governing tight, noise-free interactions (CIT [29]) and therefore robust interactions. Small dimension genes can be observed on the top of the Delaunay triangulation connected by green edges (Supplementary Information, paragraph 3.2), and they are the most co-interacting/co-regulated genes, i.e. genes robust to perturbation. Statistics on Delaunay triangulation for YMC datasets in represented in S2a Table. Examples of small dimension genes can be seen in S4 Fig, where Reductive/Charging small dimension sentinels _3.36_*ADH2*^R/C(2)^, _4.2_*ATO3* ^R/C(2)^, and _3.7_*ACS1*^R/C(2)^ are observed in the proximity of cell cycle genes _3.56_*TIP1*^M/G1(2)^, _7.57_*DSE3*^M/G1(2)^, and _7.37_*EGT2*^M/G1(2)^ which explains the demand of Coenzyme A (CoA) for genome stability and centromere/kinetochore-mediated mitotic progression [30]. Closely residing are small dimension metabolic genes _5.6_*FOX2* ^R/C(2)^, _7_*PFK26*^(2)^, _6.6_*TSL1*^G1(2)^, _4.5_*PRR2*^(2)^, and _4.4_*NCE102*^(2)^.

As is known, Diploid cells of budding yeast may enter a (2) *stationary dormant phase*, *G0*, upon starvation [17, 31, 32]. This mechanism induces Ty1, Ty2, and Ty3 transposon regulation of tRNA Polymerase III-mediated genes [33], and more than 300 long terminal repeat (LTR) genes are determined in our *Aperiodic* Cluster 3, represented by Cluster 0 in NNMF clustering (S2 Fig). *Aperiodic* Cluster 3 peaks out of the *Barycentre* to lock the cell in the dormant state when sensing the shortage of nutrition or the presence of the mating pheromone [31, 32]. Transposon genes, examples of which are _20.1_*MMS1*^Tr(0,2)^, _15.7_*RTF*^Tr(1)^, _14.1_*MMS22*^Tr(1)^, _14.0_*BRE1*^Tr(1)^, _13.1_*RRT103*^Tr(3)^, and _13.9_*ARG82*^Tr(2)^, embody the global cell surveillance mechanism and are found in each cluster (Fig 2C, blue balls).

#### Analysis of yeast cell cycle

**Fig 3:**
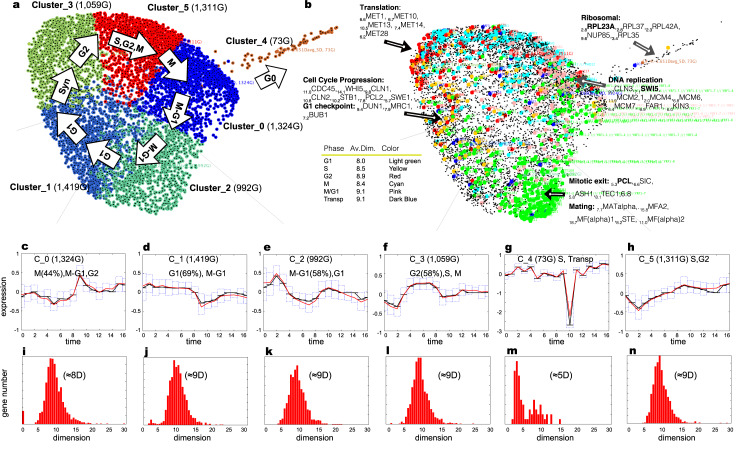
Clusters for the Yeast Metabolic Cycle (YMC) dataset (9,334G, 36 time points, 300 minutes): (a) Metabolic clusters aligned with the cell cycle. (b) YMC sentinel genes with average local dimension for each YMC phase in the table near the manifold. (c) YCC sentinels depicted by colored balls with a mean dimension for each YCC phase in the table near the manifold. Top Barycentre genes at a high local dimension are shown with respective dimensions in brackets and are pointed to with lines. (d)–(g) Cluster expression profiles, with candlesticks (solid red: average values; solid black: median values). Boxes indicate the Q25, Q50 (black), and Q75 quantiles. The x-axis shows time point tags, and Y-axis shows gene expression. (h)–(k) Dimension histograms of local dimensions (X-axes) and the number of genes (Y-axes) for respective clusters in (d)–(g). Local dimension scale of X-axes in (d)–(g) fits the scale of ambient space (X-axes) in (h)–(k).

The YCC dataset of 6,178 genes (17-time points, 160 min) (Cho, et al., 1998; Spellman, et al., 1998) separated into six clusters, and the Bregman k-means revealed an arrangement within the context of cell cycle phases, as seen in Fig 3A and S1B Fig. Fig 3B represents an allocation in clusters YCC regulator genes for the cell cycle progression identified as an anchors of each phase in the previous studies. S3 Table represents the assessment of Gene Ontology (GO) terms in six clusters and S2B Table represents statistics on Delaunay triangulation for YCC datasets.

Cluster 0 (1,324 G; GO Tag for biological process: translation 1.23E-22, mitochondrial translation 8.24E-09, regulation of transcription, DNA-dependent 1.37E-06) and Cluster 2 (992 G; GO Tag for biological process: mitotic sister chromatid cohesion 3.83E-15, DNA replication 7.97E-13, lagging strand elongation 2.03E-06) relate to mitosis and cell movement to the G1 phase (Fig 3A, 3D, 3F, 3J and 3L). Mitotic markers (Spellman, et al., 1998),—such as _9.7_CLN3M^(0)^, _7.1_SWI5M^(0)^ and DNA replication “MCM” genes by Spellman’s (Spellman, et al., 1998), control the cell cycle and regulate the mitotic exit resided in Cluster 0. Proton pumps _8.8_PMA1M^(2)^, _12.9_PMA2M^(2)^, _7.8_PMPM^(2)^, and _6.5_PMP3M^(2)^ are known to be regulated by “MCM” genes and mitotic exit genes _5.3_PCL9^M-G1(2)^, _6.6_SIC1^M-G1(2)^ and _8.1_TEC1^M-G1(2)^ genes reside in Cluster 2 nearby “MCM” genes of Cluster 0 (Fig 3B). The ‘MAT’ (Spellman, et al., 1998) genes _7.7_MATALPHA1^M(2)^, _15.8_MFA2^M(2)^, _18.7_MF(ALPHA)1^M-G1(2)^, _8.2_STE3^M-G1(2)^, ^11.0^MF(ALPHA)2^G1(2)^ are also found in Cluster 2 which is shown to be the case of mating in the presence of mating pheromone [25, 26].

Cluster 1 (1,419 G; GO Tag for biological process: transport 4.44E-09, ribosome biogenesis 2.99E-07, rRNA processing 1.12E-06) (Fig 3E and 3K) includes 207 out of 298 (70%) of G-1 phase sentinels classified by Spellman’s (Spellman, et al., 1998) and G1-phase checkpoint genes, such as _9.4_DUN1^G1(1)^, _7.9_MRC1 ^G1(1)^, and _7.2_BUB1^G1(1)^. And, finally, Cluster 3 (1,059 G; GO Tag for biological process: transcription 5.15E-13, regulation of transcription, DNA-dependent 1.37E-12, cell cycle 2.74E-07) relates to the synthesis phase (Fig 3G and 3M). It includes the ‘MET’ (Spellman, et al., 1998) genes, which are indispensable for DNA replication as a starvation for sulfur or methionine, causing G1-phase arrest. MCM and MET genes are seen near each other on the manifold in S5 Fig. Cluster 4 (73 G; GO Tag for biological process: translation 1.81E-111, translational elongation 2.42E-30, rRNA export from nucleus 1.63E-13) includes 39 small and large ribosomal subunit genes in very small dimensions (Fig 3A, 3H and 3N).

Transposon genes are also identified as spread over all clusters, as in the Tu et al. dataset, ensuring a defensive response to undesirable environmental perturbations (Fig 3B, blue balls).

In the Barycentre of the YCC datasets, transporter function genes are mainly found: _30.3_CCT2^(3)^ chaperone activity, _28.8_DJP1^(0)^ peroxisomal protein transport, _28.8_PEP8^(0)^ endosome-to-Golgi retrograde protein transport, _22.7_SWH1^(2)^ oxysterol-binding protein localized to the Golgi, _21.9_GAT4^(1)^ GATA family zinc finger motifs, _21.8_NRT1^(1)^ nicotinamide riboside transporter, and _19.9_AQY1^(0)^ spore maturation transporter.

#### Discovery on Pentachlorophenol (PCP) gene expression data

As described in Kanno et al. [34], male mice were divided into four groups with twelve each and given a single dose of pentachlorophenol (PCP) at 0, 10, 30 and 100 mg/kg by oral gavage. At 2, 4, 8, and 24 hours post-gavage, from each dose group, the liver was excised from three mice into ice-cooled plastic dishes. Tissue blocks weighing 30 to 60 mg were soaked in RNA later (Ambion Inc., TX) within 3 min of the beginning of anesthesia, finishing 12 animal sampling at each time points within 25 to 30 minutes to avoid circadian-based variation within a time point.

Forty-eight liver samples (triplicates for dose and time) were homogenated and spiked with standard external RNAs for Percellome normalization [21] and processed according to the Affymetrix standard protocol. The GeneChips used were Mouse 430–2. By the in-house software SCal4 (Spike Calculation version 4, [21, 35]), the efficiency of in vitro transcription (IVT), and the dose-response linearity of the five GSC spikes was checked, and Percellome data, i.e., absolutized mRNA copy numbers of each probe sets were generated. The data consist of four dose levels and four-time points, generating a 4x4 matrix. The mean value (m) with standard deviation (sd) was calculated from the triplicate for all of the 45,000 probe sets (PSs) generated by the GeneChip for each dose-time point.

Another in-house software named RSort (“Roughness Sort”, [36]) was used for the automatic selection of treatment-responding mRNAs. This program sorts the 45,000 PSs based on the roughness of the 3D surface. It filters the PSs by the number of peaks (three or fewer peaks) and additional parameters such as maximum expression level (more than two copies per cell), Student’s t-test p values between vehicle and top dose groups (P<0.05) leaving about 3,500 probes as significantly altered genes. These automatically pre-selected PSs were then visually checking for its 3D-surface shape for eliminating noise data, leaving 513 genes as showing biologically plausible alteration by the PCP administration.

The 513 probes were directed to the unsupervised clustering by AGCT. As a result, the NNMF method generated two clusters (S6A Fig), and the Affinity Propagation method resulted in 21 clusters (S6B Fig). Canonical pathways and Upstream regulators gave by the Ingenuity Pathway Analysis [37] showed that, in NNMF clusters, one smaller cluster was highly concentrated with genes in Interferon Signaling and another more massive cluster containing NRF2 regulated genes and others.

Affinity Propagation clusters showed a more detailed clustering of various pathways. Although divided into a few clusters, there were clusters enriched with Interferon signaling genes (clusters #5, #7, #11) and enriched with NRF2 regulated genes (clusters #4, #9, #10, #17), shown in S7 Fig. Both groups of genes were shown to be induced only at 24 hours after oral administration. This data indicated that up to 8 hours, no alterations were induced by the PCP treatment. This finding strongly suggests that some of the treatment was sequentially inducing changes to turn on the Interferon and NRF2 genes. PCP might have turned on PXR/RXR or AhR signaling to induced enzyme activity that can metabolize PCP to more toxic metabolites such as hydroquinones and/or benzoquinones. These metabolites are known to strongly turn on NRF2 via binding to the SH residues of Keap1 molecule know as a sensor to initial NRF2 activation. Indeed, PXR/RXR activation is suggested by cluster #13. Close observation of the 3D surface of the cluster member genes revealed that some P450 genes are induced from 4 hours (Cyp2a4 (1422230_s_at), Cyp7a1 (1422100_at)), all of them are known to be activated by PXR and found in cluster#13. PXR (NR1I2) itself was induced at 2 hours (1425723_at), 1451807_at), strongly supporting that PCP can trigger PXR activation, which leads to the induction of PCP metabolism to the more active forms. On the other hand, the precise molecular mechanism that PCP metabolites turn on the Interferon signaling is not clear, either by the gene ontology of the cluster members and by IPA (Ingenuity Pathway Analysis) [37] upstream analysis. However, TRIM24 is strongly highlighted by the upstream analysis, suggesting that PCP metabolites might activate Pattern Recognition Receptors to initiate Interferon signaling.

Poisoning with Pentachlorophenol produces Hyperthermia or hyperpyrexia, profuse sweating, uncoordinated movement, muscle twitching, and coma. The induction of Interferon signaling may pose new mechanism for some of those acute symptoms [34]. PCP has been shown to induced liver tumors in rodent studies and the metabolites of PCP mentioned above were considered as the cause of oxidative stress or the hydroxyl radical insults against the liver [38, 39].

#### Testing other tools in application to time-series biological data

To verify AGCT status within the context of current applications, we searched for tools/methods within the same claim scope. Table 2 (a,b,c) represents the description of the tools tested and validation results on two datasets. S8A–S8H Fig briefly visualize the results of each tool tested.

For all tools, we used a “ground truth” dataset of 3,565 periodic selected probes by Tu et al., of yeast metabolic cycle and whole yeast metabolic cycle dataset of Tu et al., that contains 9,335 probes. Data includes three periodic groups of genes active in different phases, such as oxidative (OX), reductive-building (R/B) and reductive-charging (R/C), that therefore includes top active gene probes in number of 55, 40, and 41 for 3,656 probes set and 61, 40 and 45 for 9,335 whole-cell probes set. Hence 3,565 probes are periodic; they are used as a ground truth dataset in Bushati et al. [22] study for the testing novel algorithm as well. However, a 9,335 whole-cell dataset, which includes 3,565 periodic probes and 5,770 probes that with fold change less than 1.7 folds might be intricate for the analysis. That is a reason we use these two datasets to check the performance of AGCT and other tools selected by us for testing.

First of all, it is Expander [40] analyzer with three methods CLICK, SOM, and SAMBA was applied to data, and results are in S8A–S8C Fig. While CLICK correctly separated sentinels in a smaller dataset (3,565), it fails to maintain on a big dataset (S8A Fig). SAMBA is dividing both datasets for testing into two spaces while dropping out Reductive/Building sentinels altogether (S8C Fig). Expander does not include Gene Ontology annotations, which might help to receive information on genes in clusters; biological annotations have to be brought from the other databases in case of Expander.

STEM works on short (3–8 time points) series and visualizes the behavior of genes belonging to a given Gene Ontology category, as well as AGCT does. It includes k-means and STEM original clustering methods; fifty models that are being fitted to the data given represent the data (S8D Fig). STEM correctly separated sentinels into clusters for smaller dataset of 3,565 probes, however on a big dataset of 9,334 probes, it has correctly assigned only a part of sentinel probes. A convenient option is visualizing GO terms and sorting clusters by the GO terms and their significance for clusters.

Python’s Scikit library includes many algorithms for unsupervised clustering and manifold learning, including t-SNE [22] and Isomap [16]. t-SNE is an algorithm designed for visualizing high-dimensional data by projecting each datapoint into a two or three dimensional-map. It was executed on original format time-series data, and manifold data pre-processed by AGCT and clustered by k-means. As it is seen in S8E Fig, t-SNE accurately assigned sentinels into clusters on AGCT-pre-processed data and fails on original time-series (see Tables in S8E Fig). In the case of a large dataset of 9,335 probes (S8F Fig), it failed to allocate probes in both cases (see Tables in S8F Fig). Isomap is a non-linear dimensionality reduction, like t-SNE. It was run on using the original data and manifold data provided by AGCT on the small dataset (2 tests), then made into 4 clusters using k-means. On the dataset Scikit partially clusters the sentinels into one cluster. There is no improvement from using clustering using the manifold data provided by AGCT (S8G Fig). The poor cluster might be due to the input data not having enough features. Isomap [11] is designed to handle a dimension space much greater than 36 points.

Visgenex 2.0 [22] suite runs on gene time-series data and offers thresholding by F-score, t-SNE pro, and K-means for analysis. Visgenex was able to allocate Oxidative and Reductive/Building sentinels correctly on a small data, but only partially the Reductive/ Charging sentinel (S8H Fig). Visgenex 2.0 was not able to process the big dataset of 9,335 probes.

## Discussion

In this study, we present a new clustering software tool called AGCT (A Geometric Clustering Tool) for the analysis of large-scale time-series data.

By employing a spectral manifold visualization technique and clustering methods, such as Bregman k-means++, non-negative matrix factorization (NNMF), and affinity propagation, we successfully detected the biologically meaningful architecture of the whole cell expression profile for the Yeast Metabolic Cycle (YMC) and the Yeast Cell Cycle (YCC), without data pre-processing. Data pre-processing may significantly reduce the size of the network, thus breaking the original topology the of the dataset.

The YMC (three cycles, 300 min) clustering analysis revealed an exact assessment of Reductive/Charging, Reductive/Building, and Oxidative sentinels in three clusters with periodic behaviour regulated by the aerobic/anaerobic status of mitochondrial respiration, as well as one *Aperiodic* cluster responsible for the stationary phase when a cell stops growing due to nutrient limitation. An *Aperiodic* cluster essential in YMC architecture was lost in the previous study [17], since its supervised learning approach was insensitive to unexpected discoveries. The analysis of a single cycle (160 min) of YCC data exposed six clusters that successfully fit the mitotic, growth, and synthesis phases, as verified by the allocation of phase-specific sentinels identified in a molecular biology experiment [20].

AGCT provides both a bird’s-eye view of all clusters as well as an expanded view of each small passage of data, achieved by zooming in on the manifold. The Delaunay triangulation technique can easily find strongly co-regulated genes, i.e. robust genes with small dimension score, and allows investigating their neighborhood, as no molecule can be disregarded within the dynamics of the biological network.

AGCT introduces a new value, the *dimension score*, for use in the analysis of biological data. By our analysis, we postulate that genes with similar roles have similar dimension scores, as we can see in the case of ribosomal (score 2–5), metabolic (score 5–6), and cell cycle genes (score 8–10). This tendency is observed for both YCC and YMC. Since random manifolds would have intrinsic dimensions approaching the ambient space’s (CIT [29]), and low-dimensional manifolds imply latent free variables that govern the manifold structure (CIT2 [28]), this means that the dimension score can serve as a metric for the robustness/fragility of various parts of a gene network.

AGCT allows for processing any set of synchronized time series, regardless of the number of time stamps and spacing between the time stamps. The analysis does not assume any restrictive assumption about time series. For example, we do not assume stationarity or specific shapes for autocorrelation; the wavelets we extract can capture the entire signal and efficiently learn a manifold regardless of the shape or value of these characteristics. This is another reason why the use of AGCT should be encouraged outside our primary field of genetics. Only the synchronous nature of time series is assumed, which means that there exists a fixed set of time stamps for which each time series is captured by a set of readings.

This is an important consideration for wavelet methods. Also, there is no limitation on the purpose of the analysis: instead of gene expression data, time series can be related to single nucleotide polymorphism genotyping [40], genomic clustering [41], economy development forecast, or toxicity fingerprint analysis [42].

We emphasize one pattern in these examples. Time series are grouped according to “individuals” in the broadest sense; an individual can be a gene (which is the main focus of our paper), a fermentor, a person, a crop field, and so on. The manifold, therefore displays these “individuals”, not in a way, without connection to displays obtained using conventional factorial analyses, but in a much more sophisticated and powerful way. The best choice of parameters for each of the different applications should then be data-driven, and this is a reason why AGCT integrates such a large spectrum of choices for all the steps, starting from the number and types of wavelet transforms available, and including the number, types, and sophistication of clustering algorithms on a manifold. We have extensively addressed this problem in our specific focus example of gene expression data, and we provide parameter choices that we think are reasonably close to the most accurate ones and bring results that support our findings. AGCT should thus be also thought as a contender to other pieces of software that offer visualization capabilities, but do not necessarily contain the amount of sophistication and state of the art algorithms we have brought in AGCT for these purposes.

## Methods

Algorithm workflow and various techniques of AGCT are described in Supplementary Information file. Here, we simly summarize the steps and options in AGCT that we used to obtain the results for each of the datasets.

### Encoding

The Yeast Metabolic Cycle (YMC) and Yeast Cell Cycle (YCC) datasets were encoded with n = 64 on Haar wavelets, and the results were similar to those obtained with n = 32.

### Prototype / feature selection

Computations were performed on all genes in the YMC and YCC datasets without using the prototype or feature selection mechanisms of AGCT. Note that AGCT is capable of processing (tens of) thousands of gene expression profiles on a commodity computer. For example, a set of 20,000 gene expression profiles yields a gene x gene similarity matrix **W** of 20 K x 20 K double precision floating point numbers (approximately 3 GB), which easily fits in, say, 32 GB RAM. Furthermore, its computation time is short; the computation of **W** on YCC and YMC takes less than 15 minutes on a 2.8 Ghz Intel Core i7 MacBook Pro. Each clustering roughly takes 8 x 20,000 x k (the number of clusters) bytes to store the results. Therefore, even when k = 100, 16 MB is enough to store the results of a hundred distinct clusterings on the whole dataset. This makes it easy to process large numbers of clusterings and to perform extensive statistical analyses.

### Building a sparse weighted graph representation by computing W

We compute the similarity matrix W whose entries are defined as the cosine similarity between the corresponding genes (see Supplementary Information sec. 2.4 for other choices of distances between gene feature vectors like the heat kernel similarity). To capture the manifold structure, we first define a weighted graph representation of the genes by sparsifying the dense similarity matrix by reducing the number of non-zero entries using the k-symmetric nearest neighbors. The k-symmetric nearest neighbors are obtained by selecting the k largest similarities for each gene and then taking the logical-or of rows and columns with the same index in order to keep its symmetric property.

The sparse symmetric matrix W encodes the weighted adjacency matrix of a graph of genes, and the choice of k is linked to the dimension of the clusters we seek to retrieve. In our experiments, we chose k = 10, but we observed that small variations in k did not produce qualitatively different results (see SI, sec. 2.4).

### Clustering by solving an eigenvalue problem for a stochastic matrix N

N is a normalized stochastic matrix (calculated from similarity matrix W) that represents the transition matrix of a Markov chain whose states are genes. In SI, sec. 2.5, we report six different methods to produce a row stochastic matrix N, whose row coefficients sum up to one. We then solve the eigenvalue problem for N.

### Clustering Bregman k-means

We used Bregman k-means clustering (without using the Iterative option) with the squared Euclidean distance. We obtained N = 4 as the number of clusters for YMC and N = 6 for YCC. S1a, S1B Fig displays the curve that justifies the choice of k. For YMC, the choice is optimal with k = 4 (and the clustering that was kept is the best for this value of k). The reason it is optimal is because the standard deviation is much smaller for the other values of k, indicating an easier problem, and because the split between the two regimes of the curve occurs at k = 4 (a sharp decrease before, mild decrease after). For YCC, the situation is a bit different and the choice of k is made for different reasons. Indeed, there is no “hard" clustering part as with YMC for k = 4. The break occurs at k = 4, but k = 6 is preferred because it provides clusters that are well aligned with YCC; hence, it provides a better interpretation of the clustering results.

### Delaunay triangulation

S3A and S3B Table displays the statistics of the Delaunay triangulation for YMC and YCC for different values of *p*. Both tables (a,b) show that the observed (i.e., significant) edges are short compared to the length of the average edge, which is a good sign since it means that genes whose profiles have the largest correlations tend to be brought nearer to each other on the manifold.

### Recording scenario

The scenario is a series of actions on AGCT with parameters. Recording is started automatically after the application is launched (The scenario icon blinks.) When the “Scenario save” button is pressed, the recording is saved into a text file. By loading the scenario users can reproduce the parameter settings and results up to the calculation of the manifold. This feature saves time by eliminating the need to memorize parameters and manually process the dataset each time. Parameters used for computing YMC and YCC data are described in sections 3.1–3.6.

## Supporting information

S1 File (PDF)

S2 File (PDF)

S3 File (ZIP)

S4 File (ZIP)

S1 Data (XLS)

S2 Data (XLS)

S1 Table Major GO tags that define four clusters of Tu et al dataset (9,334G), with p-value < 10E−3. P–GO biological process, F–molecular function, C–cellular component. (DOCX)

S2 Table Statistics of Delaunay triangulation for YMC (a) and YCC (b), for different p-values.
(PDF)

S3 Table Major GO tags that define six clusters of Spellman CDC28 et al dataset (6,178G), with p-value < 10E−2. P–GO biological process, F–molecular function, C–cellular component. (DOCX)

S1 Fig Black line is an average k-means potential (with arthur-vassilvitskii initialization); red area is ±σ area around mean. (PDF)

S2 Fig (a) Two clusters obtained with Non-Negative Matrix Factorization method on the Yeast Metabolic Cycle dataset (9,334G): Cluster 0 (596G) and Cluster 1(8,738G).(b) YMC and YCC sentinel genes belong to Cluster 1, space where most colored circles are observed. Delaunay triangulations are shown with green edges. (c, d) Cluster expression profile, with candlesticks (solid curves: in red, average values; in black, median values). Boxes indicate Q25, Q50 (black), and Q75 quantiles. (e, f) Dimension histograms for each cluster.
(PDF)

S3 Fig The transition between M-G1 phase (Reductive/Charging cluster 2) and G1 phase (Oxidative cluster 1) of Yeast Metabolic Cycle. Reductive/Charging and Oxidative sentinels are detected in groups up (blue) and down (light green) on the manifold edges (a,b).(a) M-G1 phase sentinels bridging two clusters to promote cell cycle progression. (b) High local dimension genes of YMC are in the core of Reductive/Charging cluster 2 and around “M-G1—G1” bridge area (a), specifying Barycenter of YMC dataset.
(PDF)

S4 Fig A network of highly co-regulated periodic genes (Spellman et al.) extracted on top of Delaunay triangulation (p≤0.001) of Reductive/Charging Cluster 2 (4,572G). Blue—Metabolic genes (ADH2, ACS1, ATO2, FDH1, POX1, FOX2), Red—YCC genes (EGT23, DSE3, TIP1, TSL1, Spellman at al.), Green—Yeast Cell Cycle (NCE102 PFK26, PRR2) identified in this study. The thick red line corresponds to the enlarged region.
(PDF)

S5 Fig View of ‘MCM’ DNA replication genes and ‘MET’ methionine synthesis genes on the manifold. (PDF)

S6 Fig Clustering results on 513 pentachlorophenole probes. (**a**) NNMF (k = 2), (**b**) Affinity propagation (k = 21), (**c**) Delaunay Triangulation on top co-regulated genes (p≤0.001, green edges).
(PNG)

S7 Fig Two sub-networks Interferon (#6, #8, #12) and NRF2 (#5, #10, #11, #18) signaling networks have been identified by Affinity propagation on 513 probes. Green and red edges equivalent to positive and negative correlations according to Delaunay triangulation (p≤0.001).
(PDF)

S8 Fig Results of analysis on bioinformatics tools tested using two Tu et al., dataset: 9,335 probes dataset and 3,656 probes dataset, referred as “ground truth” dataset. (PDF)
