# Supplementary material for: A Geometric Clustering Tool (AGCT) to robustly unravel the inner cluster structures of time-series gene expressions
Source: PLoS One. 2020 Jul 6;15(7):e0233755. doi: 10.1371/journal.pone.0233755 (PMC7337352; doi:10.1371/journal.pone.0233755)
Supplement: S1 File — (PDF) [file pone.0233755.s001.pdf]

Supplementary Information to A Geometric Clustering Tool (AGCT) to robustly unravel the inner cluster structures of time-series gene expressions

Richard Nock   Natalia Polouliakh   Frank Nielsen   Keigo Oka
Kazuhiro Shibanai   Hiroaki Kitano

The workflow of **AGCT** essentially consists of two parts: a set of algorithms that execute processes ranging from loading the data to displaying manifold and a set of algorithms that post-process the manifolds.

1 The scenarii mechanism of AGCT: ensuring fast reproducibility of processing

In **AGCT**, scenarii represent a specific macro-instruction language that allows the user to record and replay interactions and perform various computations (e.g., perform manifold computations or run clustering algorithms). Scenarii in **AGCT** allow the user to automate virtually any subset of the workflow: each scenario records a range of information, from the datasets loaded as parameters into the clustering algorithms to specific options for making 3D displays of the manifolds. Thus, for example, instead of providing a distant colleague with only a still snapshot (e.g., jpeg picture) of a manifold created from a particular dataset and a particular viewpoint, a researcher can provide him or her with the actual dataset used and a scenario that will automatically load and process the dataset until the corresponding 3D display with the particular viewpoint is obtained. After having processed a scenario, the colleague can then execute further processing stages (e.g., triangulation) and record them as new scenarii.

A scenario allows the user to automate processing in two ways: s/he can record the set of parameters used to run a specific algorithm (e.g., the number of clusters or the number of iterations in k-means) or record the set of parameters and the output of the algorithm (in the case of k-means, the clusters obtained). This latter possibility was implemented for two reasons: first, it enables fast reprocessing of data, and at the end of the scenario, the software produces a transcript (a set of values for the algorithms' inputs and outputs) that is virtually the same as the one from which the scenario was recorded. Second, some algorithms, like Forgy initialization in k-means, integrate a random component. Recording the output of k-means in the scenario guarantees a "replay" that exactly matches the run from which the scenario was recorded.

In addition to providing "dynamic reproducible" pictures of manifolds, the scenarii mechanism allows newcomers to **AGCT** to (re)run a complete set of experiments by making a single click to load a scenario. Finally, scenarii are stored in text format with explicit tokens, so that even a user minimally familiar with **AGCT** can easily modify the text of a scenario, for example, to select a different clustering algorithm.

S1 Fig. summarizes the options available in **AGCT** regarding scenarii. S2 Fig. shows part of an example scenario for the YMC dataset; the scenario allows the user to alter the display of the results directly: here, the Java interface is modified at scenario run time to make it look like somebody is interacting with it in order to perform the modifications recorded in the scenario. In addition, some of the visualisation parameters can be used to alter the 3D display automatically.

2 Part I: from data loading to displaying manifolds

S6 Fig. summarizes the different algorithms involved. The subsections below detail the different steps.

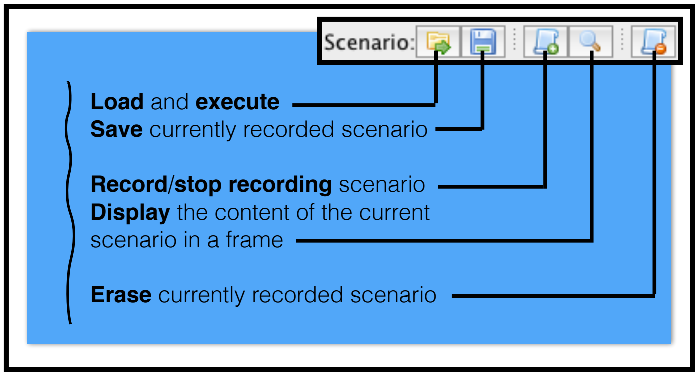

S1 Figure: Options available in **AGCT** for recording, loading and executing scenarii.

```
AGCT_Modification_Sort_Depth|0
AGCT_Modification_Sort_Depth|0
AGCT_Modification_Perspective|0
AGCT_Modification_Sort_Depth|0
AGCT_Modification_Use_Shadow|0
AGCT_Modification_Perspective|0
AGCT_Modification_Method_F|0
AGCT_Modification_Method_S|1
AGCT_Modification_Method_W|1
AGCT_Modification_Method_N|1
Load|/Users/rnock/Experimentations/Yeast_Tu.agct
AGCT_Modification_Number_Of_Wavelet_Stamps|256
JAGCTSelectionPane_Method_F|1
AGCT_Modification_Number_Of_Neighbors|10
AGCT_Modification_Number_Of_Wavelet_Stamps|64
JAGCTSelectionPane_Validate|
JAGCTManifoldPane_Manifold_Begin|
@Ordered_List_Names_Begin
10000_at|10001_at|10002_i_at|10003_f_at|10004_at|10005_a
10031_at|10032_at|10033_at|10034_at|10035_at|10036_at|10
10062_at|10063_i_at|10064_s_at|10065_at|10066_s_at|10067
10092_at|10093_at|10094_at|10095_at|10096_at|10097_at|10
10124_at|10125_at|10126_at|10127_at|10128_at|10129_at|10
```

```
9941_at|9942_at|9943_at|9944_at|9945_at
9976_at|9977_at|9978_at|9979_at|9980_at
AFFX-25srRnaa_at|AFFX-25srRnab_at|AFFX-
BioDn-5_st|AFFX-CreX-3_at|AFFX-CreX-3_s
ThrX-5_at|AFFX-ThrX-M_at|AFFX-TrpnX-3_a
AFFX-YFL039CM_at
@Ordered_List_Names_End
@Matrix_W_Begin
220|1.0|565|1.0|750|1.0|1866|1.0|2183|1
5|1.0|319|1.0|350|1.0|437|1.0|1170|1.0|
1233|1.0|4738|1.0|6881|1.0|7926|1.0|812
147|1.0|474|1.0|529|1.0|556|1.0|558|1.0
2280|1.0|2951|1.0|3887|1.0|4242|1.0|456
```

S2 Figure: Two parts of a scenario showing the scenarii tags and the way they are arranged. Scenarii are recorded using three types of special tags. The first type are tags recording the results of computations (*e.g.*, @Matrix_W_Begin), the second type are keywords recording some parameters that will help to make sure the data stored in the scenario is associated in the right way with the data stored in the data file (*e.g.*, @Ordered_List_Names_Begin, @Ordered_List_Names_End; the data file is also displayed, as Yeast_Tu.agct). The third type consists of tags referring to the parameterisation of the 3D displays (*e.g.* AGCT_Modification_Use_Shadow and AGCT_Modification_Sort_Depth), which makes it easy, via scenarii, for the user to redo the computations and to get the intended focus on the 3D results (manifold or PCA).

```
@DOMAIN_NAME Yeast_Tu_9335

@GENE_ANNOTATIONS Yeast_Tu_tags.txt

@GROUPS None
@TAG    GEN     YY      0.0     1.0     2.0     3.0     4.0     5.0     6.0     7.0     8.0     9.0     10.0    11.0    12.0    13.0    14.0    15.0    16.0    17.0    18.0
19.0    20.0    21.0    22.0    23.0    24.0    25.0    26.0    27.0    28.0    29.0    30.0    31.0    32.0    33.0    34.0    35.0

@DATA
10000_at        0.055618618     0.073988438     0.120317824     0.055118114     0.030303033     0.026143791     0.013001083     0.09195403      0.088377729     0.062780268
0.079908676     0.06395939      0.157290474     0.079545453     0.065462753     0.059360731     0.109253071     0.031632651     0.013144589     0.056155507     0.108571425
0.036045313     0.096566521     0.067527309     0.145299137     0.114792898     0.081447966     0.063894525     0.078512393     0.033613447     0.108671792     0.090234853
0.053846158     0.068897635     0.061678458     0.034232363
```

S3 Figure: Data format: a simple case (Yeast Metabolic Cycle, YMC dataset). The tag @TAG gives the list of time stamps of each time series. In this case, it says that each gene records a set of 36 time stamps, regularly spaced from 0.0 to 35.0. GEN and YY are dummy names not used in this case.

2.1 Sketch of data format

Each row in the dataset is associated with a gene; the gene's ID comes first in the row, and it is followed by a set of time series that is associated with the gene. At the beginning of the file, the user provides the number of points in each time series and the time stamps associated to each of these points. For each gene, the total number of records in the corresponding row must match the total number of time stamps. There is no other constraint on the time series. In particular, time stamps do not have to be regularly spaced and their number does not have to be a power of two. S3 Fig. presents an example of formatting in a simple case. S4 Fig. presents a more involved case. Here, each gene is associated with a set of time series instead of a single time series, and each refers to a particular modality of the @TAG variable.

After having read the data, **AGCT** computes features, selects prototypes and features and learns the manifold(s).

2.2 Computing features

Let g be a gene, and let f'_g be a time series of its expression profile. The time series are transformed using one of the two options:

1. each time series corresponds to one slope coefficient in a linear model;

```
@DOMAIN_NAME Gene_Regulation_BCell

//@GENE_ANNOTATIONS must be followed by the gene annotations file name
//In addition, the annotation file MUST be in the same directory as this file

@GENE_ANNOTATIONS Natalia_gene_annotations.txt

//@GROUPS MUST precede any @TAG token
//Time stamps MUST follow for each TAG, on the SAME line

@GROUPS AMPC-CA2+       Test

@TAG    2MA     YN      T1      0.5 1.0 2.0 4.0
@TAG    Anti-Ig YY      T2      0.5 1.0 2.0 4.0
@TAG    BAFF    NN      T1      0.5 1.0 2.0 4.0
@TAG    BLC     YY      T2      0.5 1.0 2.0 4.0
@TAG    BOM     YN      T2      0.5 1.0 2.0 4.0
@TAG    CD40L   YN      T1      0.5 1.0 2.0 4.0
@TAG    CGS     YN      T2      0.5 1.0 2.0 4.0
@TAG    CpG     NN      T1      0.5 1.0 2.0 4.0
@TAG    DIM     YN      T2      0.5 1.0 2.0 4.0
@TAG    ELC     YY      T1      0.5 1.0 2.0 4.0
@TAG    fMLP    NN      T2      0.5 1.0 2.0 4.0
@TAG    GHRH    YN      T1      0.5 1.0 2.0 4.0
@TAG    IFNb    NN      T1      0.5 1.0 2.0 4.0
@TAG    IFNg    NN      T1      0.5 1.0 2.0 4.0
@TAG    IGF-1   YN      T2      0.5 1.0 2.0 4.0
@TAG    I10     NN      T1      0.5 1.0 2.0 4.0
@TAG    I04     NN      T2      0.5 1.0 2.0 4.0
@TAG    LPA     YY      T2      0.5 1.0 2.0 4.0
@TAG    LPS     NN      T1      0.5 1.0 2.0 4.0
@TAG    LTB4    YN      T2      0.5 1.0 2.0 4.0
@TAG    M3A     NY      T1      0.5 1.0 2.0 4.0
@TAG    NEB     YN      T1      0.5 1.0 2.0 4.0
@TAG    NGFb    NN      T2      0.5 1.0 2.0 4.0
@TAG    NPY     NN      T1      0.5 1.0 2.0 4.0
@TAG    PAF     NN      T2      0.5 1.0 2.0 4.0
@TAG    PGE2    YN      T1      0.5 1.0 2.0 4.0
@TAG    SIP     YN      T1      0.5 1.0 2.0 4.0
@TAG    SDF1a   YY      T1      0.5 1.0 2.0 4.0
@TAG    SLC     YY      T1      0.5 1.0 2.0 4.0
@TAG    TER     YN      T1      0.5 1.0 2.0 4.0
@TAG    TGF     NN      T1      0.5 1.0 2.0 4.0
@TAG    TNFa    YN      T1      0.5 1.0 2.0 4.0

@DATA
20 -0.32165331 0.03935158 0.19360963 0.12290593 -0.03505208 -0.06864807 -0.18947158 -0.31824834 -0.22587460 0.2902
-0.07945253 -0.04131497 -0.09861530 -0.24700184 -0.02288732 0.15011163 -0.15876589 -0.06031716 0.01627137 0.107520
0.00848477 -0.13340076 0.09509929 0.31019243 0.10547013 0.22252258 -0.32183713 0.19390556 0.06306638 0.33388793 0.
-0.19337485 0.15304730 0.43940163 0.24592102 0.37501337 -0.52133109 -0.02269307 -0.00019310 0.15963693 -0.21618985
0.01311254 0.36238704 0.28037717 0.35636196 0.14993345 0.33173529 0.42111110 0.49851335 0.17605268 0.52392156 0.24
0.00273950 0.38710869 @ANNOTATION this is a test for the annotation of the first gene
```

S4 Figure: Data format: a more complex case. Each gene records 32 time series (=32 @TAG), each of which contains 4 time stamps, for a total of 128 records per gene. Each tag here is associated to a ligand (*e.g.* 2MA, Anti-Ig, BAFF, etc.) with two characteristics per ligand. The first takes values in {YY, YN, NY, NN} and the second in the set {T1, T2}. These characteristics are used to group the tags together for an easier processing of the genes.

2. each time series brings a series of n' wavelets coefficients. The available wavelets models are Daubechies and Haar wavelets.

As an example, we provide a derivation of the Haar wavelet coefficients (other wavelet models have similar steps):

1. choose the number of time stamps n', a power of two. There are two possible ways of choosing in **AGCT**. The first is an automatic guess that best fits the data and uses at most 256 time stamps; the second is that the user chooses;
2. interpolate the time series using n' regularly spaced time stamps;
3. computate the wavelet coefficients.

The interpolated time series yields f_g, a piecewise constant function ready for Haar wavelet decomposition. If, for the sake of readability, we make the assumption that the support of f_g is normalized to fit in $[0, 1]$, then the Haar wavelet decomposition of f_g is given by:

$$f_g \doteq f_{g0} + \sum_{j=0}^{N} \sum_{i=0}^{2^j-1} f_{gi}^j \psi_i^j \ , \quad (1)$$

where:

$$f_{g0} \doteq \langle f_g, \phi \rangle \ , \phi(x) = 1, \forall 0 \leq x < 1 \ , \quad (2)$$
$$f_{gi}^j \doteq \langle f_g, \psi_i^j \rangle \ , \psi_i^j(x) \doteq \sqrt{2^j}\psi(2^j x - i) \ , \quad (3)$$

and:

$$\psi(x) \doteq \begin{cases} 1 & \text{if} \quad 0 \leq x < 1/2 \\ -1 & \text{if} \quad 1/2 \leq x < 1 \end{cases} \ . \quad (4)$$

Here, $\langle f, g \rangle \doteq \int f(x)g(x)\mathrm{d}x$. There are 2^{N+1} coefficients. **AGCT** offers the option to discard f_{g0}, the average value of f_g which does not cause a variation of f_g over time and keep the remaining $n = 2^{N+1} - 1$ coefficients in a vector representation $\boldsymbol{x}_g$ of the time series. Each time series of each gene is then processed using the same model (same wavelets, same n'). At the end of the algorithm, the wavelet coefficients of the time series for each gene are concatenated into a feature vector.

2.3 Reducing the size of data

Manual time series selection Prior to computing the wavelet coefficients, the user can select sets of time series that will be removed from the computation (option available only when genes are described using more than one time series).

Automatic feature selection In **AGCT**, the user can reduce the number of features in the description vectors n, using a greedy procedure that iteratively removes one feature until a certain number $n_r < n$ is reached. The feature removed in each iteration is the one that has the largest coefficient of determination, r^2, with respect to the remaining features. At the end of the procedure, the subset of n_r features that are kept has smaller linear correlation in it than in the whole set. The $n - n_r$ features that were not selected are not included in the subsequent analysis (manifold computation and post-processing).

Automatic prototype selection When there is not enough memory to perform manifold learning on all the data, **AGCT** integrates a prototype selection method that selects, out of the m' total initial genes, a subset $m < m'$ on which the learning is to be done. Prototypes are selected using our Bregman k-Means Algorithm (see below). This algorithm has certain theoretical guarantees and can fit spherical clusters that spread regularly throughout the data. At the end of the k-means algorithm, each cluster center is replaced by its closest gene from the data; should a gene be able to be put in two clusters, the second closest gene to the current cluster center is selected. Thus, each prototype on the manifold represents a set of *prototype group genes* with similar profiles. The k-means algorithm is run for a maximum of 30 iterations. It halts earlier if the current relative decrease in the k-means within clusters sum of squares is below 10^{-10}.

2.4 Computing a similarity matrix W between genes

Similarity measures Prior to computing the manifold, the user picks a similarity measure to compute the similarity between each pair of genes. The user can choose from three possible similarity measures, each being symmetric and returning a value in $[0, 1]$. For any two genes g and g' and representation vectors $\boldsymbol{x}_g$ and $\boldsymbol{x}_{g'}$, the similarity between g and g', $\mathrm{sim}(\boldsymbol{x}_g, \boldsymbol{x}_{g'})$, can be measured in terms of:

- the cosine similarity,

$$\begin{aligned}\mathrm{sim}_c(\boldsymbol{x}_g, \boldsymbol{x}_{g'}) &\doteq \frac{1}{2}\left(1 + \cos(\boldsymbol{x}_g, \boldsymbol{x}_{g'})\right) \\ &= \frac{1}{2}\left(1 + \frac{\langle \boldsymbol{x}_g, \boldsymbol{x}_{g'} \rangle}{||\boldsymbol{x}_g||_2 ||\boldsymbol{x}_{g'}||_2}\right) \ ; \end{aligned} \quad (5)$$

- the absolute cosine similarity,

$$\begin{aligned}\mathrm{sim}_a(\boldsymbol{x}_g, \boldsymbol{x}_{g'}) &\doteq |\cos(\boldsymbol{x}_g, \boldsymbol{x}_{g'})| \\ &= \left|\frac{\langle \boldsymbol{x}_g, \boldsymbol{x}_{g'} \rangle}{||\boldsymbol{x}_g||_2 ||\boldsymbol{x}_{g'}||_2}\right| \ ; \end{aligned} \quad (6)$$

- or the heat kernel similarity,

$$\mathrm{sim}_h(\boldsymbol{x}_g, \boldsymbol{x}_{g'}) \doteq \exp\left(-\frac{\|\boldsymbol{x}_g - \boldsymbol{x}_{g'}\|_2^2}{T}\right) \ , \quad (7)$$

  where $T > 0$ is chosen by the user.

From now on, we will assume that there are m genes, either prototypes ($m < m'$) or initial genes ($m' = m$ if no prototype selection is necessary). A similarity above is used to build an $m \times m$ initial similarity matrix W, whose general term is:

$$(\mathrm{W})_{gg'} \doteq \mathrm{sim}(\boldsymbol{x}_g, \boldsymbol{x}_{g'}) \ . \quad (8)$$

Sparsification of W Sparsification is useful to capture the local structures of the manifold [1]. The user has two options to reduce the number of non-zero entries in W, thereby sparsifying the matrix. The first consists of transforming W into a symmetric nearest neighbor matrix: we compute, for each gene g, its k-symmetric nearest neighbors (SNN_g^k) [2], that is, the set of genes that are its k most similar genes, according to the chosen similarity $\mathrm{sim}(.,.)$. This boils down to binarizing each row of W, assigning the value "1" to the k largest similarities (and "0" to the others), and then taking the logical-or of rows and columns with the same index. To summarize the first sparsification, the general term of sparsified matrix $\mathrm{W} \in \{0,1\}^{m \times m}$ is of the form:

$$(\mathrm{W})_{gg'} \doteq \begin{cases} 1 & \text{if} \quad g' \in \mathrm{SNN}_g^k \\ 0 & \text{otherwise} \end{cases} \quad (9)$$

(we have assumed for brevity that g can also represent the corresponding gene's index). The second kind of sparsification is straightforward: the user specifies a percentage p%, and the computer removes the p% smallest similarities, replacing them by 0, and ensuring that the matrix W remains symmetric.

2.5 Computing the manifold coordinates — non linear case

Matrix W is normalized into an $m \times m$ matrix N that is then eigendecomposed, each of its $n' + 1 > 3$ non-trivial leading eigenvectors then giving a coordinate to each of the m genes. By leading eigenvectors, we mean those corresponding to the largest eigenvalues of N [3, 4]:

$$\begin{aligned}\mathrm{N}\boldsymbol{e}_i &= \lambda_i \boldsymbol{e}_i \ , i = 1, 2, ..., n' + 1 \ , \\ & \lambda_1 \geq \lambda_2 \geq ... \geq \lambda_{n+1} \ .\end{aligned}$$

Depending on the method chosen to compute N, we eventually discard the first eigenvector $\boldsymbol{e}_1$, and keep the n' remaining ones to obtain the coordinates of each gene in the resulting manifold:

$$\boldsymbol{x}_g^{\mathrm{m}} \doteq [\boldsymbol{e}_{2g}, \boldsymbol{e}_{3g}, ..., \boldsymbol{e}_{(n'+1)g}]^\top \ . \quad (10)$$

If $\boldsymbol{e}_1$ is not trivial, then $\boldsymbol{x}_g^{\mathrm{m}} \doteq [\boldsymbol{e}_{1g}, \boldsymbol{e}_{3g}, ..., \boldsymbol{e}_{n'g}]^\top$. There are six methods to compute N.

Markov chain normalization We compute diagonal matrix $\mathrm{D} \in \mathbb{N}^{m\times m}$ with:

$$(\mathrm{D})_{gg} \doteq \sum_{g'=1}^{m} (\mathrm{W})_{gg'} \ . \tag{11}$$

Then, we compute the normalized matrix $\mathrm{N} \in [0,1]^{m\times m}$ with:

$$\mathrm{N} = \mathrm{D}^{-1}\mathrm{W} \ . \tag{12}$$

Matrix N is a stochastic matrix and as such, it is a transition matrix of a Markov chain whose states are genes. It can be shown that $\lambda_1 = 1$ and $\boldsymbol{e}_1$ is a stationary distribution of the Markov chain described by N[3, 4]. Since this vector $\boldsymbol{e}_1$ is known beforehand, it is considered trivial and can be discarded.

Normalized graph Laplacian Using the definition of matrix D in eq. (11), we now compute

$$\mathrm{N} = \mathrm{D}^{-1/2}(\mathrm{W} - \mathrm{D})\mathrm{D}^{-1/2} \ . \tag{13}$$

Again, it can be shown that $\lambda_1 = 0$ and $\boldsymbol{e}_1 = \mathrm{D}^{1/2}\mathbf{1}$ [5], so we discard this first trivial eigenvector, and keep the n' remaining ones to obtain the coordinates of each gene in the resulting manifold as in (10).

Assortative and dissassortative Bethe Hessian Using again the definition of matrix D in eq. (11), we compute the Bethe Hessian normalized matrix N as:

$$\mathrm{N} = r\mathrm{W} - \mathrm{D} - (r^2 - 1)\mathrm{I} \ , \tag{14}$$

where I is the identity matrix. This recent technique has been mainly analyzed in the context of undirected graphs [6, 7], so we will restrict its use with symmetric nearest neighbors sparsification of matrix W (Subsubsection 2.4). In addition, it works best when $r = \pm\sqrt{c}$, where c is the expected degree of the graph [7]. In our case, we consider $c = 2k$, since $2k$ is the average degree of the SNN^k graph.

There are two flavor of Bethe Hessian, one which capture the assortative interactions, *i.e.*, those bringing together genes with large profile similarities, for $r = \sqrt{c}$, and one which captures the dissassortative interactions, for $r = -\sqrt{c}$.

Doubly stochastic approximation A N is doubly stochastic iff both N and $\mathrm{N}^\top$ are stochastic, where "$\top$" denotes transpose. This technique is similar to Markov chain normalization, as it seeks the symmetric doubly stochastic matrix N that best approximates W, by solving the following optimization problem:

$$\arg \min_{\mathrm{N}\in\mathbb{R}_+^{m\times m}} \|\mathrm{N} - \mathrm{W}\|_F^2, \quad \text{s.t. } \mathrm{N} = \mathrm{N}^\top, \mathrm{N}\mathbf{1} = \mathbf{1} \ , \tag{15}$$

where $\|.\|_F$ denotes Frobenius norm [8].

Doubly stochastic scaling and approximation It was previously remarked that the Markov chain normalization and the normalized graph Laplacian are in fact two first steps of different algorithms that iteratively compute a doubly stochastic approximation of W [9, 8]. The doubly stochastic approximation, however, creates a drift between the eigensystems of N and W. Our last normalization of W involves a preprocessing stage, computing the intermediate matrix W_i that is the closest to the doubly stochastic matrix under the constraint that its eigenspace is the *same* as W. Then, a doubly stochastic approximation is carried out on W_i instead of W. In general, this significantly reduces the number of iterations in the doubly stochastic approximation. Because scaling is much faster than doubly stochastic approximation, together they are significantly faster than doubly stochastic approximation alone.

2.6 Computing the manifold coordinates — linear case

To compare with nonlinear manifold learning, we have also implemented PCA in **AGCT**, thus providing the user with a 3D linear manifold display and correlation balls for the axes that are selected in the 3D display. S5 Fig. exemplifies this possibility.

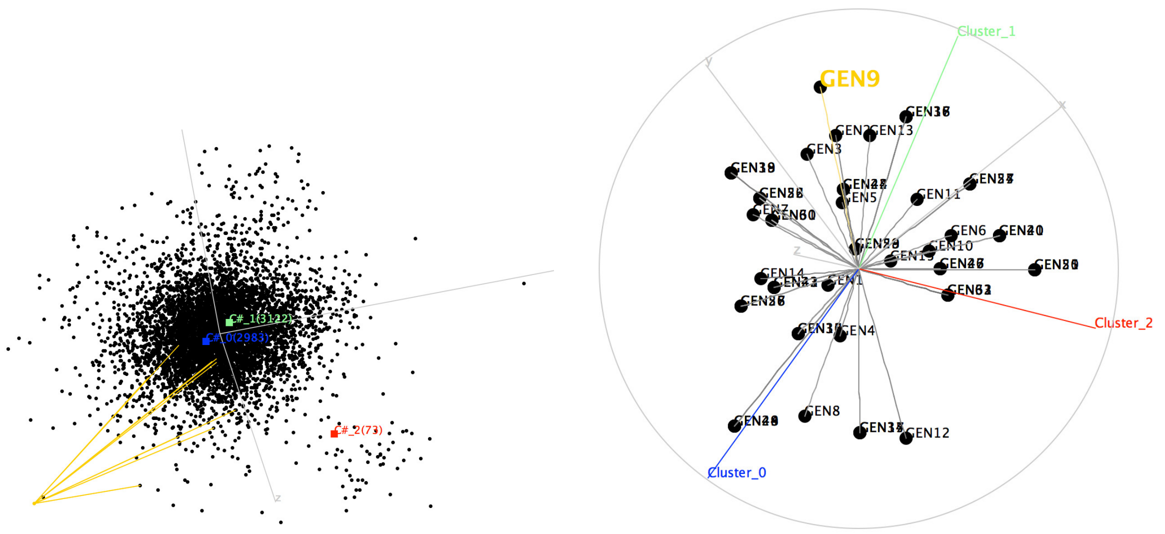

S5 Figure: Example of PCA results for the YCC (Yeast Cell Cycle) data (left) and correlation ball for the three lead axes (right). **AGCT** shows on the correlation ball the clusters obtained with a hard clustering algorithm, indicating the influential variables in the clusters; the interpretation of the influence on the cluster axes is the same as on the regular axes [10]. The yellow edges are Delaunay edges extracted from the non-linear manifold.

3 Part II: post-processings on the manifolds

S7 Fig. summarizes the post-processing workflow, starting with the display of the manifolds. The different steps in the workflow are described below.

3.1 Clustering algorithms

After the manifolds are computed and displayed, the main post-processing stages are the ones that usually follow manifold learning algorithms [6], namely clustering the manifold to reveal the clusters of genes whose profiles are similar to each other (because they are in general in similar regions of the manifold). There are six method families, and they include various options and improvements over the standard methods. All clustering algorithms share the following properties:

1. the data used to cluster genes can be: the complete non-linear manifold coordinates (default 20 dimensions), the current 3D non-linear manifold coordinates, or the current 3D PCA coordinates;

2. for algorithms that necessitate a guess as to the number of clusters (all algorithms except affinity propagation), the user can specify the number of clusters, or a range of integers for the cluster number. **AGCT** then runs the chosen clustering algorithms for each of the specified values.

3. for algorithms integrating a random component, which affects the final result (all algorithms except hierarchical clustering), there is also the possibility to automatically rerun the same clustering algorithm a specified number of times. If the user has specified the range of the number of clusters, **AGCT** runs the clustering algorithm for *all* specified values.

Furthermore, the clusterings are run from a specific pane, shown in S8 Fig. There is no limit on the number of clusterings stored, except for the memory allocated to the Java virtual machine. The clustering algorithms can be classified into two categories: hard membership clustering algorithms, which limit the clusters in which each gene belongs to exactly one, and soft membership clustering algorithms, for which each gene belongs to all clusters in variable proportion. For the latter algorithms, **AGCT** also computes a hard membership for each gene by picking the cluster with the largest proportion.

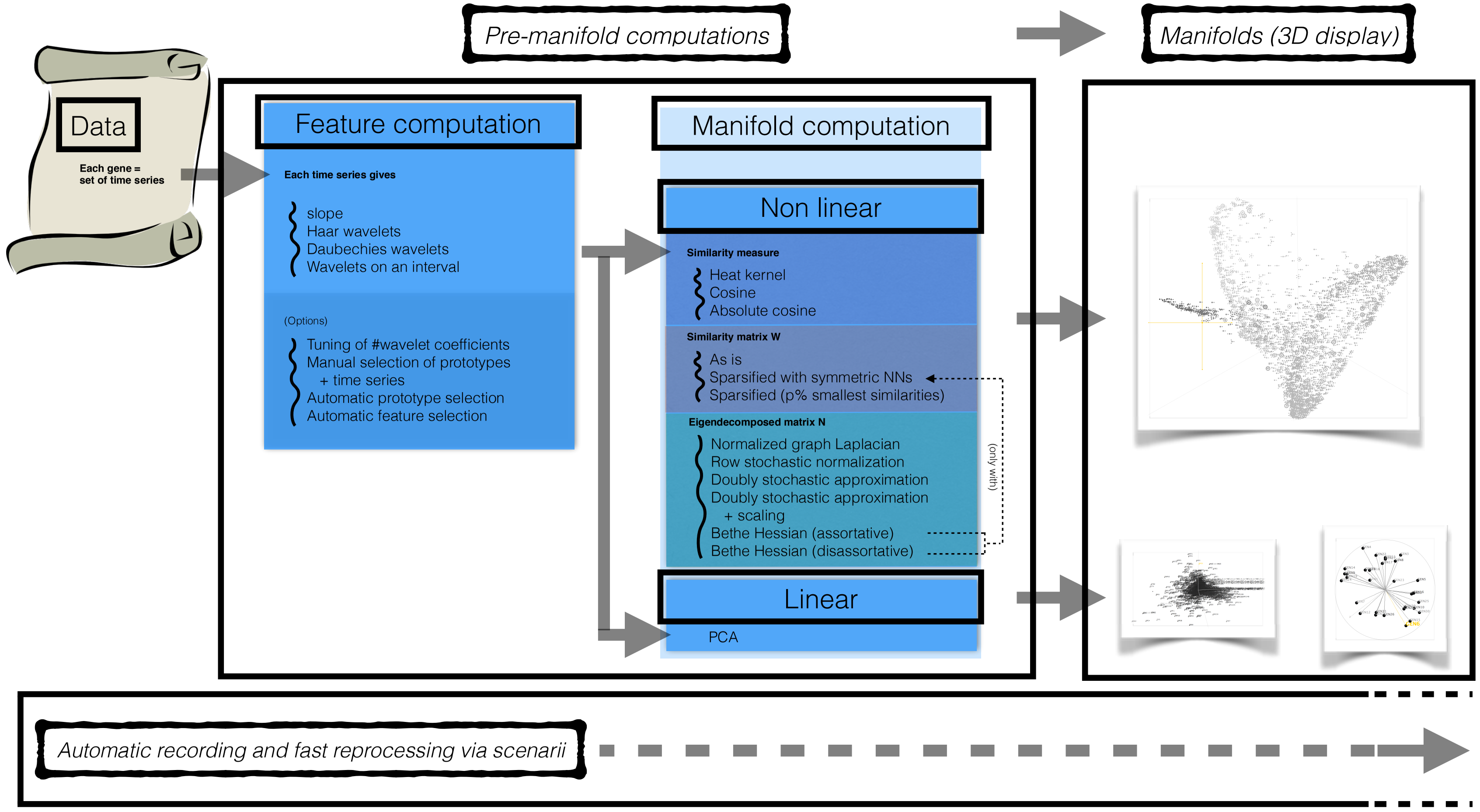

S6 Figure: Summary of the workflow from loading the data to computing and displaying the manifolds. Each step can be recorded in a scenario and replayed for automated and fast reprocessing.

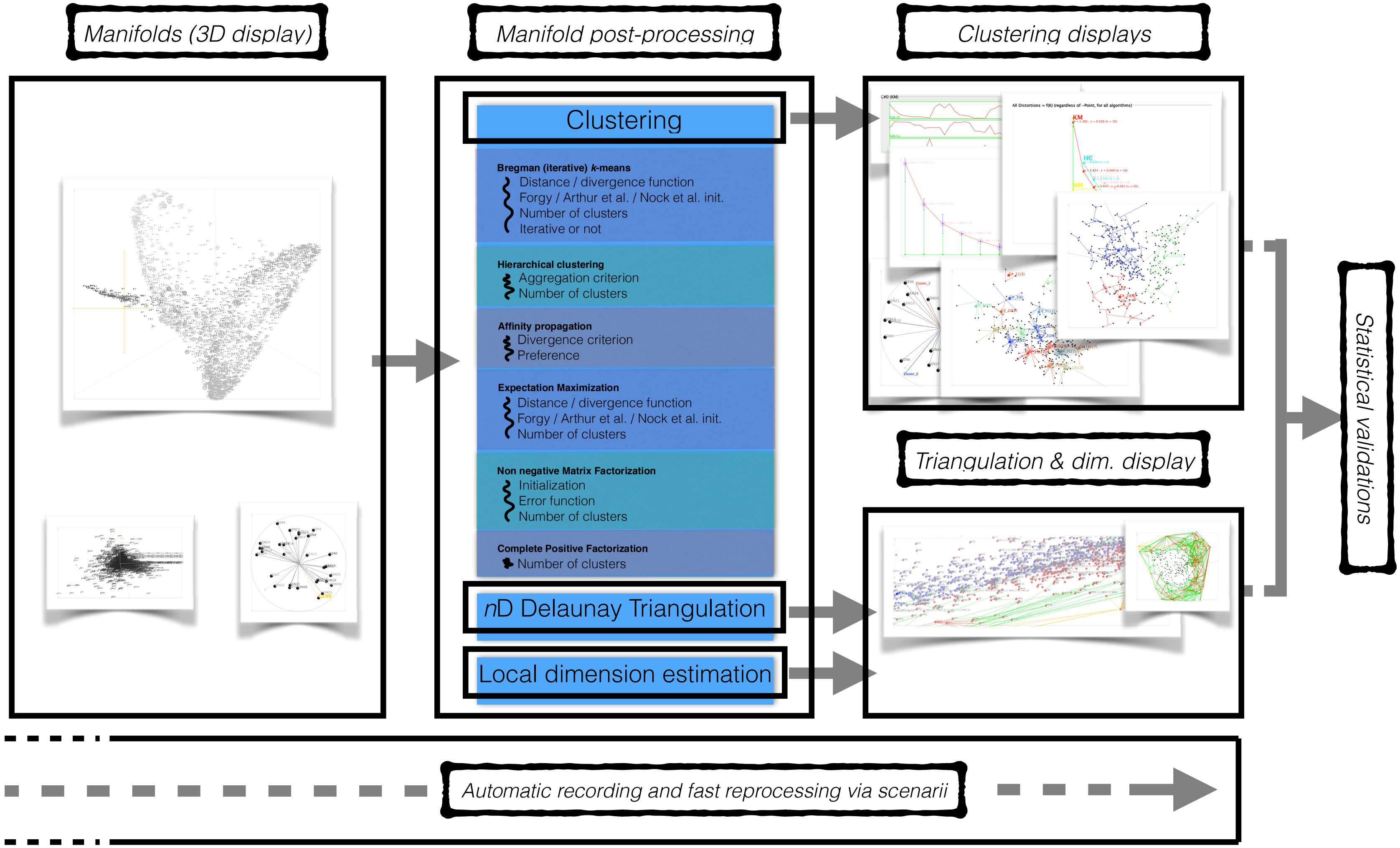

S7 Figure: Summary of the post-processing workflow, starting with the display of the manifolds. Similarly to the manifold pre-processing each step can be recorded in a scenario and replayed for automated and fast reprocessing.

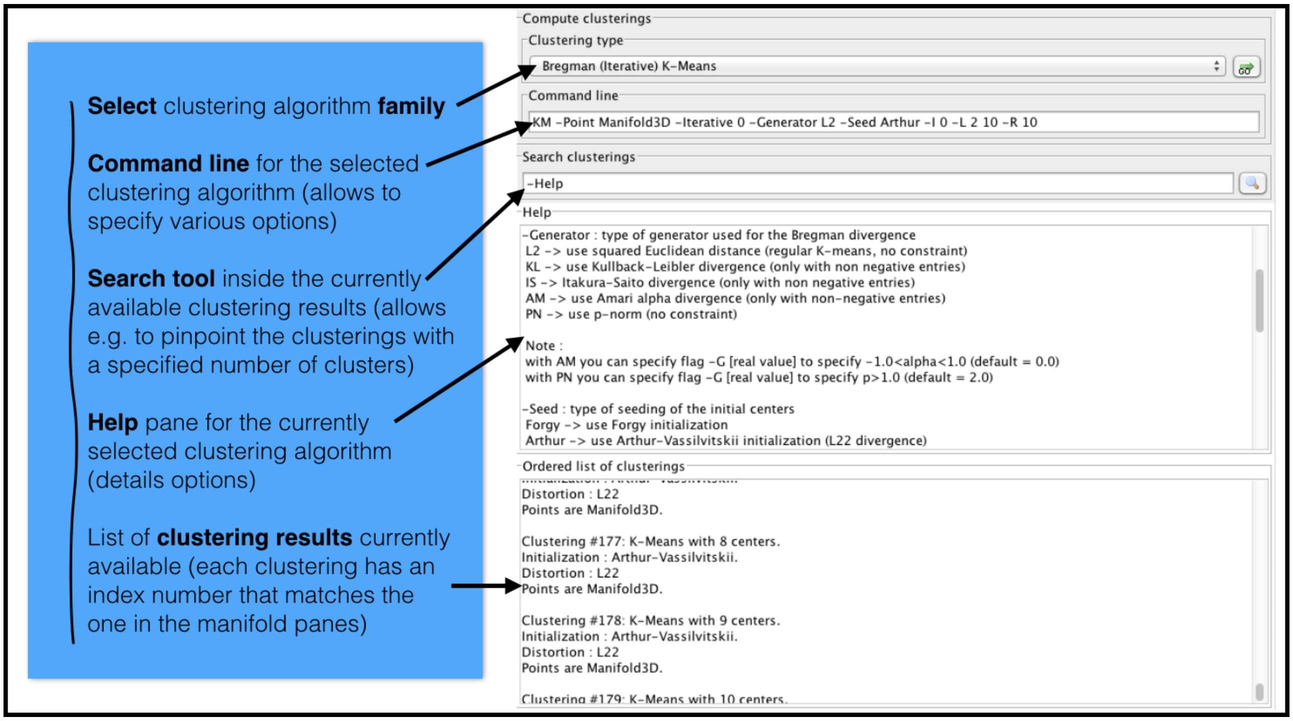

S8 Figure: Clustering pane of **AGCT**, allowing the user to select and parameterize the clustering algorithm and search the current set of clusterings.

Bregman iterative k-Means Clustering **AGCT** implements a generalization of Arthur-Vassilvitskii's algorithm for initializing the cluster centers in a generalization of k-means type algorithms to Bregman divergences[11, 12] (see Algorithm 1). The Bregman divergence $\Delta_\psi(\boldsymbol{x}_g||\boldsymbol{x}_{g'})$ between the vector representations of two genes g and g' is [13]:

$$\Delta_\psi(\boldsymbol{x}_g||\boldsymbol{x}_{g'}) \doteq \psi(\boldsymbol{x}_g) - \psi(\boldsymbol{x}_{g'}) - \langle \boldsymbol{x}_g - \boldsymbol{x}_{g'}, \boldsymbol{\nabla}_\psi(\boldsymbol{x}_{g'})\rangle \ , \tag{17}$$

where ψ is a strictly convex differentiable function. **AGCT** includes popular examples of Bregman divergences: the squared Euclidean distance, Kullback-Leibler divergence and Itakura-Saito divergence, *i.e.*,

$$\begin{aligned} D_{\mathrm{E}}(\boldsymbol{x}_g||\boldsymbol{x}_{g'}) &\doteq (\boldsymbol{x}_g - \boldsymbol{x}_{g'})^\top(\boldsymbol{x}_g - \boldsymbol{x}_{g'}) \ , \\ D_{\mathrm{KL}}(\boldsymbol{x}_g||\boldsymbol{x}_{g'}) &\doteq \sum_{i=1}^n x_{gi}\log(x_{gi}/x_{g'i}) - x_{gi} + x_{g'i} \ , \\ D_{\mathrm{IS}}(\boldsymbol{x}_g||\boldsymbol{x}_{g'}) &\doteq \sum_{i=1}^n (x_{gi}/x_{g'i}) - \log(x_{gi}/x_{g'i}) - 1 \ . \end{aligned} \tag{18}$$

The complete clustering algorithm works by replacing the usual k-means squared Euclidean distance by an arbitrary Bregman divergence. Its initialization phase, Bregman seeding, which chooses the initial cluster centers, works by repeatedly choosing a gene according to a biased distribution that favors examples that are far from the currently selected centers, as shown in algorithm 1[12]. This initialization is powerful in that, for any chosen k, if we denote opt_k as the *global* optimum for the clustering cost with k clusters[14], then after the initialization, we are already on average within a factor of order $u_\psi \log(k)$ of opt_k, where u_ψ is a factor depending on ψ and equals 1 for the Mahalanobis distortion, the generalization of the squared Euclidean distance. After initialization, the iterative phase of k-means is carried out as usual, with the important difference that the Bregman divergence chosen for the initialization phase still replaces the squared Euclidean distance[14]. We have also extended the algorithm to include an (optional) iterative clustering mechanism that allows it to escape

Algorithm 1: Bregman Seeding($\mathcal{S}$, k, ψ)

Input: Dataset $\mathcal{S}$, integer $k > 0$, Bregman divergence generator ψ;
Let $\mathcal{C} \leftarrow \{\boldsymbol{x}\}$;
//where $\boldsymbol{x}$ is chosen uniformly at random in $\mathcal{S}$;
for $i = 1, 2, ..., k-1$ **do**
  Pick at random point $\boldsymbol{x} \in \mathcal{S}$ with probability:

$$\pi_{\mathcal{S}}(\boldsymbol{x}) \doteq \frac{D_\psi(\boldsymbol{x}||\boldsymbol{c}_{\boldsymbol{x}})}{\sum_{\boldsymbol{y}\in\mathcal{S}} D_\psi(\boldsymbol{y}||\boldsymbol{c}_{\boldsymbol{y}})} , \quad (16)$$

  //where $\boldsymbol{c}_{\boldsymbol{x}} \doteq \arg\min_{\boldsymbol{z}\in\mathcal{C}} D_\psi(\boldsymbol{x}||\boldsymbol{z})$;
  $\mathcal{C} \leftarrow \mathcal{C} \cup \{\boldsymbol{x}\}$;
Output: Set of initial centroids $\mathcal{C}$;

local minima. This mechanism makes local changes by iteratively changing the cluster of a single point that decreases the cost function of k-means. The complete algorithm is a hard membership clustering algorithm.

Expectation maximization We implemented Gaussian expectation maximization [15] and augmented it with the same options for initializing clusters as in k-means (Forgy type, or the generalization of Arthur-Vassilvitskii's initialization). This is a soft membership clustering algorithm.

Complete positive factorization Complete positive factorization is reminiscent of doubly stochastic approximation [16]. Given a matrix X, we wish to find a matrix U that maximizes tr(XU) under the constraint that U is doubly stochastic, complete positive and admits a non-negative decomposition

$$\mathrm{U} = \mathrm{H}^\top \mathrm{H} ,$$

where H is a nonnegative $k \times m$ matrix, each row of which indicates all the clusters to which the gene belongs and in which proportion. This is a soft membership clustering algorithm.

Hierarchical clustering We have implemented hierarchical clustering with Ward and single linkage criteria for the aggregation of clusters. It is a hard membership clustering algorithm.

Affinity propagation Affinity propagation [17] is a message passing algorithm that yields a hard clustering solution whose number of clusters is not fixed in advance; it depends on a preference parameter P input by the user. In **AGCT**'s implementation, the user can specify a range of preferences, and the algorithm is then run for each integer in the range. Each preference defines a quantile whose preference is chosen as an initialization. Affinity propagation has an advantage over the other clustering algorithms: each cluster contains an *exemplar* [17], that is, a gene which is supposed to be the most representative of all genes in its cluster. One can provide a high-level description of the algorithm as a process alternating between genes exchanging two different types of messages until convergence. In the first round, each gene $\boldsymbol{x}_i$ sends a real-valued *responsibility* message to each gene $\boldsymbol{x}_k$ that indicates the extent that $\boldsymbol{x}_k$ may serve as an exemplar for $\boldsymbol{x}_i$. Then, each $\boldsymbol{x}_k$ sends a real-valued *availability* message to each $\boldsymbol{x}_i$, indicating the extent to which $\boldsymbol{x}_i$ may choose $\boldsymbol{x}_k$ as an exemplar. Before the next round of message exchanges begins, each gene selects its exemplar on the basis of the current availabilities and responsibilities [17]. Each cluster is thus a set of genes having chosen the same exemplar. This is a hard membership clustering algorithm.

Non-negative matrix factorization Non-negative matrix factorization [18] decomposes a $n \times m$ non-negative matrix X of data into a product of non-negative matrices,

$$\mathrm{X} \approx \mathrm{UH} ,$$

where H is a $k \times m$ matrix, each row of which indicates the clusters to which a gene is a member and in which proportion. Since our initial matrix X may have negative entries, we use two kinds of coding to make it non-negative. The first consists of crafting a $2k \times m$ matrix$\text{H}$, wherein the top half of a column encodes the positive coordinates, and the bottom half encodes minus the negative coordinates [19]. The second kind is simpler; it consists of subtracting the minimal coordinate of H from all coordinates. This is a soft membership clustering algorithm.

Comparing clusterings Clustering is a significant part of **AGCT**. Since the various algorithms it supports have numerous parameters, **AGCT** includes a graphical UI that makes it easy to compare the results of different clustering algorithms and select the most appropriate one.

First, each clustering result is directly displayed on the manifold (non-linear or PCA). For hard clustering results, the membership of each gene is represented by a disk colored the same as the cluster. For soft membership results, a pie chart is computed and displayed for each gene. In addition, the dendrogram of hierarchical clustering can be directly displayed on the manifold (non-linear, PCA), and the exemplars of affinity propagation can also be displayed with the genes of their clusters. The manifold panes also display of a subset of the clusters and their genes.

Second, additional graphical windows allows one to compare the results of different clustering algorithms. In particular, the "clustering results visualization frame" computes the average distortion as a function of the number of clusters k for each algorithm. The ability to compare such plots makes it easier for the user to pick *e.g.* the right k and right algorithm; the best run of the given algorithm can be found by conducting a searching in the clustering pane (See S8 Fig.). This window also allows one to compute a sequence of clustering losses for a specific clustering algorithm, a set of kernel similarities computed over each cluster, and dendrograms associated with the hierarchical clustering results. S9 Fig. summarizes some of the options available in this frame (top plots and bottom-left plot). Furthermore, as shown in S5 Fig., **AGCT** is able to display a clustering on the correlation ball of the PCA (see [10]).

3.2 Filtered Delaunay triangulation of the manifolds

We compute the Delaunay triangulation [20] of a manifold (either non-linear or PCA) from the three-dimensional view. The Delaunay triangulation is the dual of the Voronoi diagram, which partitions the manifold into m cells. Under the assumption that the genes are in distinct positions, cells and genes are in a bijection, with each cell being the volume capturing all points that are closer to the gene than to any other gene. Cells sharing a common frontier define a Delaunay edge between the genes. Thus, a Delaunay edge embodies a similarity between two genes that is not shared with others. The set of edges and genes define the complete Delaunay graph.

This graph is typically large, but it is useful for analyzing manifold's geometry, as it captures the whole data. Its filtering and analysis provides us with a meaningful tool to address the quality of the three-dimensional manifold's geometry, with respect to the information of the whole data it captures. A "good" manifold should first and foremost be one in which there are short edges between highly (anti-)correlated genes relative to the average length of all Delaunay edges. The user may keep the most highly correlated edges by specifying a p-like value to filter out any Delaunay edges between two genes g and g' for which:

$$\cos^{-1}(\boldsymbol{x}_g, \boldsymbol{x}_{g'}) \quad \in \quad [\pi \times p/2, \pi \times (1 - p/2)] \ (\mathrm{mod}\pi) \ . \tag{19}$$

AGCT displays all other edges by using two color codes: red is for edges for which $\cos^{-1}(\boldsymbol{x}_g, \boldsymbol{x}_{g'}) > \pi \times (1 - p/2) \ (\mathrm{mod}\pi)$, indicating a significant negative correlation between profiles. Green is for edges for which $\cos^{-1}(\boldsymbol{x}_g, \boldsymbol{x}_{g'}) < \pi \times p/2 \ (\mathrm{mod}\pi)$, indicating a significant positive correlation. Then, **AGCT** shows the ratio τ_m between the longest remaining edge and the longest Delaunay edge, as well as the ratio τ_a between the average length of remaining edges and the average length of Delaunay edges.

A good manifold should also be locally consistent with regard to filtered edges. Consider the set of all triangles on these edges. Any such triangle could have four configurations: three green edges (all positive correlations: 3/0), three red edges (three negative correlations: 0/3), two green and one red edge (2/1), and one green and two red edges (1/2). If high absolute correlations are borne out from the edges and the manifold is meaningful, then two configurations should almost never be observed: (0/3) and (2/1). **AGCT** compute the total number τ_f of such inconsistent configurations.

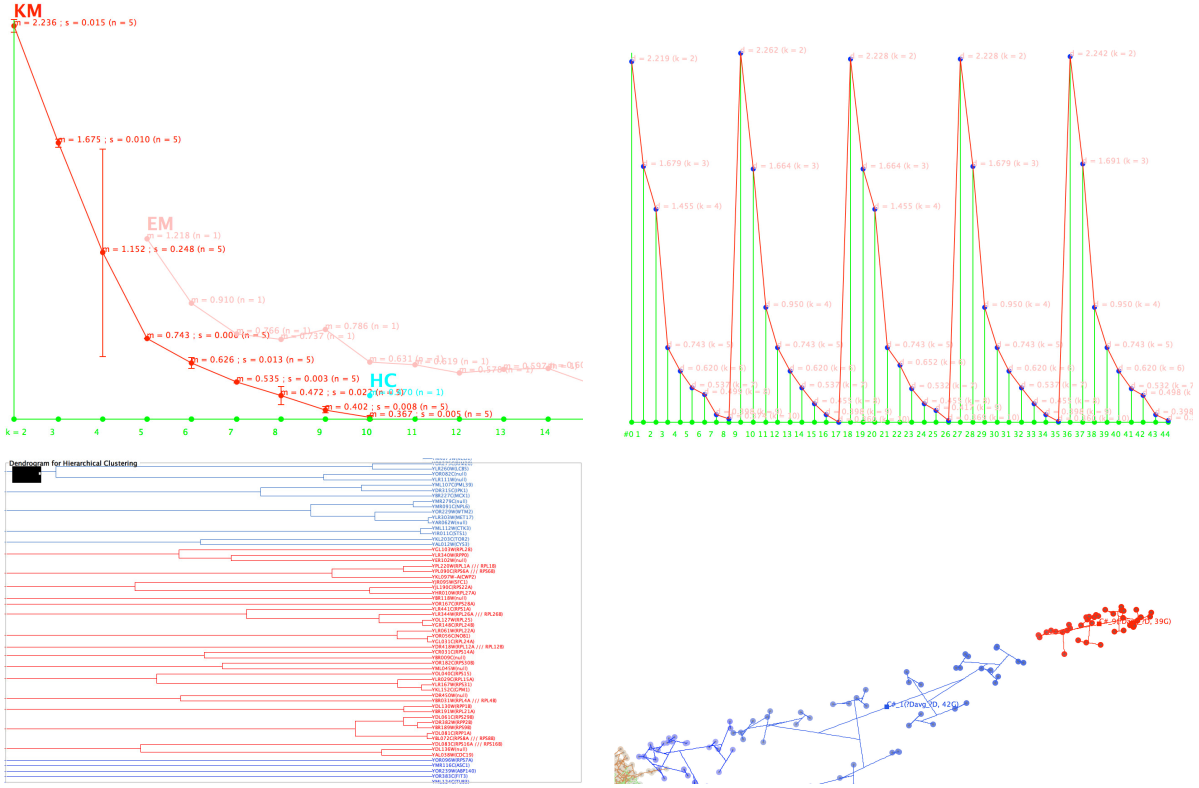

S9 Figure: Upper left: **AGCT**'s interface plots the clustering loss of all clustering algorithms, together with separate curves indicating the losses of each clustering algorithm. Here, more than fifty different runs of k-Means, EM and Hierarchical clustering are simultaneously displayed (m is the average, s standard deviation, and n the number of runs). Upper right: users can choose to display the sequence of clustering losses for one type of clustering algorithm, in this case, the k-Means, which allows for a finer comparison of losses, and select the most accurate values for k. Here, for example, the best clustering with $k = 2$ is the first one. Lower left: **AGCT** can visualize dendrograms of hierarchical clusterings and zoom/drag the dendrograms. The black rectangle in the upper left of this figure shows the whole dendrogram, and the white rectangle within it is the region currently being magnified. Lower right: users can also visualize clustering results directly on the manifolds with eventual structures specific to clusterings (Shown here is part of a dendrogram of hierarchical clustering for two clusters; see the text).

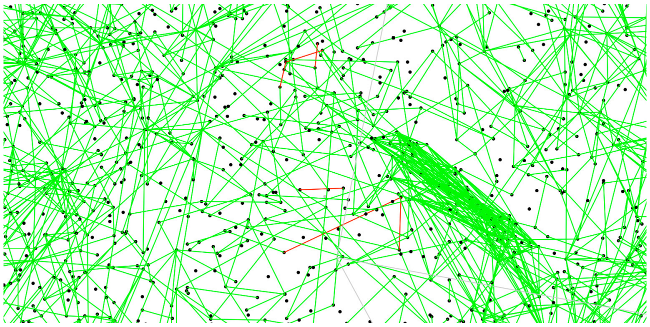

```
(*) Checking Delaunay properties for P-Delaunay = 0.5:
(**) Total observable edges / # >0 Corr. / # <0 Corr.
= 32026 / 32010 ( = 99.95004059201898%)
/ 16 ( = 0.049959407981015426%)
(***) Average observable length / Average length =
1.1953427754206976E-5 / 1.8562549880269796E-5 = 0.644
(****) Max obervable length / Max length =
0.006511828762950842 / 0.01499257108426231 = 0.434
(*****) Triangle consistency: found 14766 triangles,
(all+) pattern = 14766, (two-) pattern = 0
(one- pattern = 0, all- pattern = 0)
```

S10 Figure: Left: example of Delaunay triangulation (YCC) for p=.5. Most edges are green, but a few are red. Moreover, the distribution of significant edges is not uniform; there are dense patches containing a number of significant edges (center-right), indicating groups of heavily correlated profiles. Right: excerpt of statistical analysis of the displayed triangulation. (*) indicates the p value. (**) displays the total number of edges, as well as the number of green and red edges. Here, red edges represent $16/32026 = 0.05\%$ of all edges. (***) and (****) are τ_m- and τ_a-length statistics for the displayed edges, and (*****) is the triangle consistency analysis of the triangulation, showing in this case that there is no inconsistency.

S10 Fig. summarizes an example run of Delaunay triangulation on the YCC data.

3.3 Local manifold dimension estimation

Whereas the 3D view only gives an approximation of the actual manifold, **AGCT** also provides a way to estimate the true local dimensions of the manifold for each gene. The smaller the value, the larger the chance that the surrounding genes, as displayed on the 3D manifold, will be accurate. Furthermore, the local dimension is an indication of the local number of significant distinct "principal components" that explain the differences between a genes expression profile and those of its neighbors. In the case of PCA, these principal components would be the same for all genes. In our case, the manifold is globally non-linear but locally approximated by a linear manifold. Hence, these principal components give indications that are locally similar to PCA, and they may vary from gene to gene. This illustrates a significant power of non-linear manifolds that linear manifolds, as learned, *e.g.*, by PCA, do not have.

The technique we have used to estimate the intrinsic dimension is that of Levina and Bickel with the correction of MacKay and Ghahramani [21, 22]. More specifically, for a fixed a number of neighbors $k > 0$, we estimate the local dimension depending on k, $\hat{d}_g(k)$ for each gene g:

$$\hat{d}_g(k) \doteq \left(\frac{1}{k-1}\sum_{j=1}^{k-1}\log\frac{\|\boldsymbol{x}_g - \boldsymbol{x}_{g(k)}\|_2^2}{\|\boldsymbol{x}_g - \boldsymbol{x}_{g(j)}\|_2^2}\right)^{-1} , \quad (20)$$

where $g(j) \in \mathrm{NN}_g^k$ is the jth nearest neighbor of g. To smooth out the estimation and because there is no "best" value of k *a priori*, we compute, as the estimator of the local dimension for gene g, the harmonic average of $\hat{d}_g(k)$ over a fixed range of values for k:

$$\hat{d}_g \doteq (k_2 - k_1 + 1)\cdot\left(\sum_{k=k_1}^{k_2}\frac{1}{\hat{d}_g(k)}\right)^{-1} . \quad (21)$$

Here, **AGCT** sets k_1 to 10 and k_2 to 40.

S11 Fig. shows an example in which **AGCT** processed toy data, revealing the 2D/3D manifold and locally estimating dimensions. The local dimension $\hat{d}_.$, which is 1 (rounded) for each periodic profile, matches our intuition that profiles that are locally similar to one profile all vary according to a single parameter, the shift in the signal. In real-world domains, any gene with a large $\hat{d}_.$ may interact directly or indirectly in a number of distinct cellular processes or functions that are weakly correlated under the experimental conditions.

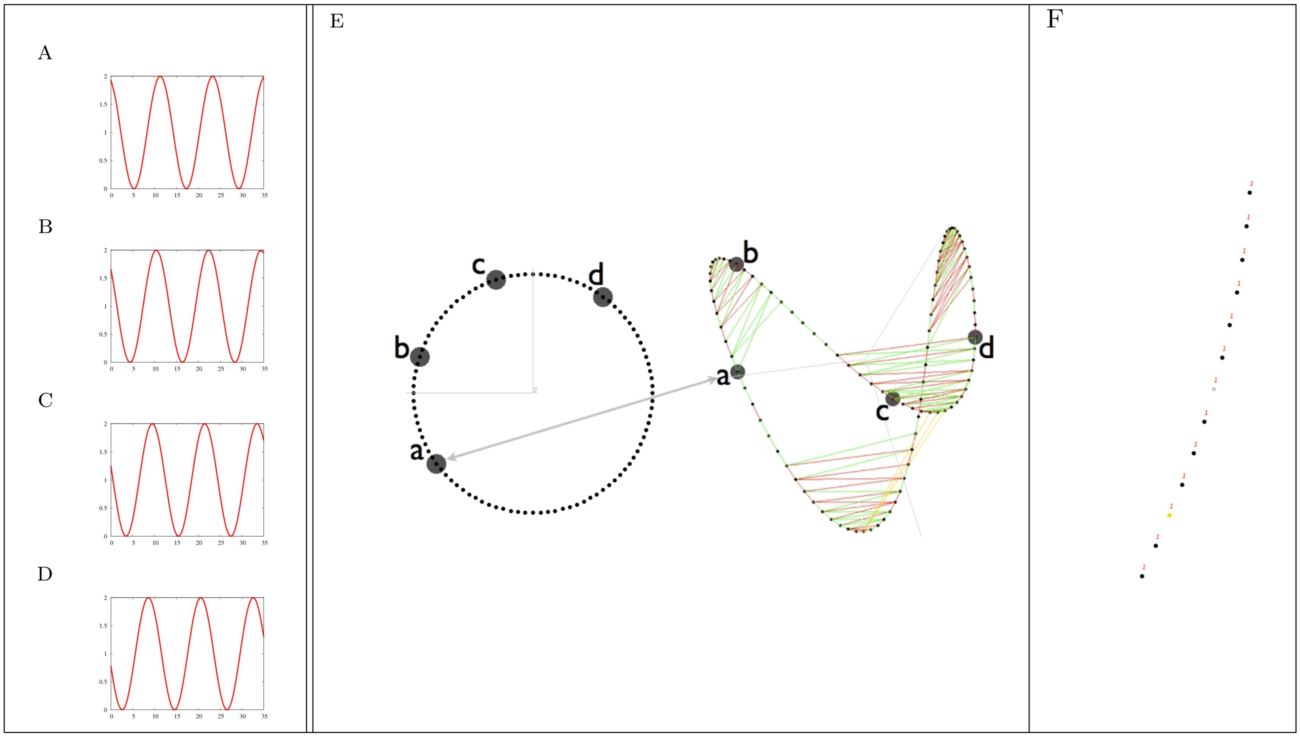

S11 Figure: Extraction of periodicity. A-D: four profiles of a toy periodic dataset processed by **AGCT**. E: 2D-planar view of the manifold on the xy plane (left), and 3D-spatial view of the manifold (right). Green and red edges are filtered Delaunay edges, each implying a significant positive or negative correlation between profiles, respectively. Yellow edges indicate the current focal point. The planar view captures the periodicity of the profiles (circle), and browsing it clockwise reconstructs the sequential ordering of the profiles. F: local dimensions displayed for part of the data. Average local dimension is 1 for all points, as expected from theory.

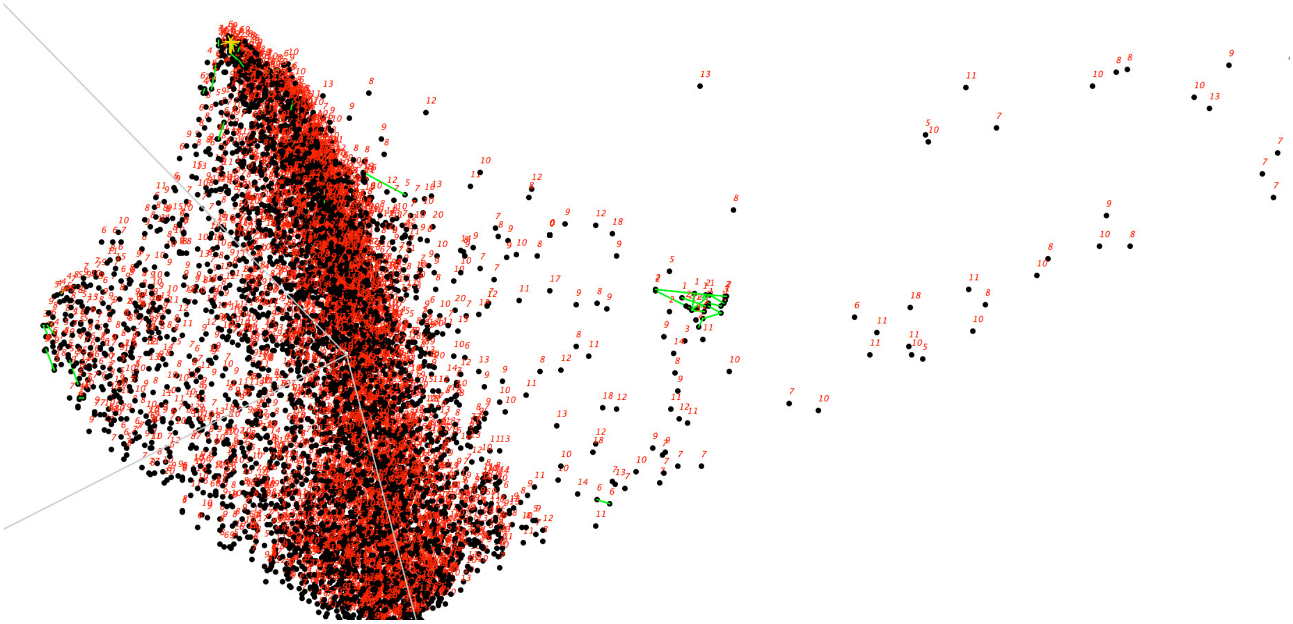

S12 Figure: Display showing local manifold dimensions and significant edges of the Delaunay triangulation ($p = .15$, Subsection 3.2).

Cluster → Pattern ↓	Yes	No
Yes	YY	YN
No	NY	NN

S1 Table: A 2×2 contingency table to test the independence assumption between a pattern in tags and a cluster.

Consequently, the local dimension estimation may vary with the experimental conditions, *e.g.*, as a function of whether they are favorable or not to the cells. A rule of thumb is that if (independent) noise is injected to data, independently of the coordinates, the intrinsic dimension increases to match, or approximately match, that of the ambient space. On the other hand, if the local dimension is very small, as for example in S11 Fig., it means that the local interactions are the result of a small number of free latent (unknown) parameters [23]. In the case of S11 Fig., the intrinsic dimension is one, since a single translation with a constant size allows one signal to be transformed into its direct neighbors.

S12 Fig. shows the local dimensions on the YCC data and the significant edges of the Delaunay triangulation for a small value of p. The genes with most of the (green) edges turn out to have a local dimension of approximately one.

3.4 Statistical post-processing of clusterings and manifolds

AGCT is able to carry out a number of statistical analyses on the outputs of manifold learning and clusterings. Here, we give a brief outline of them.

First, **AGCT** can store and process statistically the GO tagging of genes according to Biological (F)unction, (P)rocess and Cellular (C)ompartment. For any hard clustering, it is possible to build 2×2 χ^2 tables that test the independence between a pattern (in a tag) and a cluster. S1 Table is such a table. From this table, we find that $m = YY + YN + NY + NN$. The p-value is that of a χ^2 independence test with the Yates

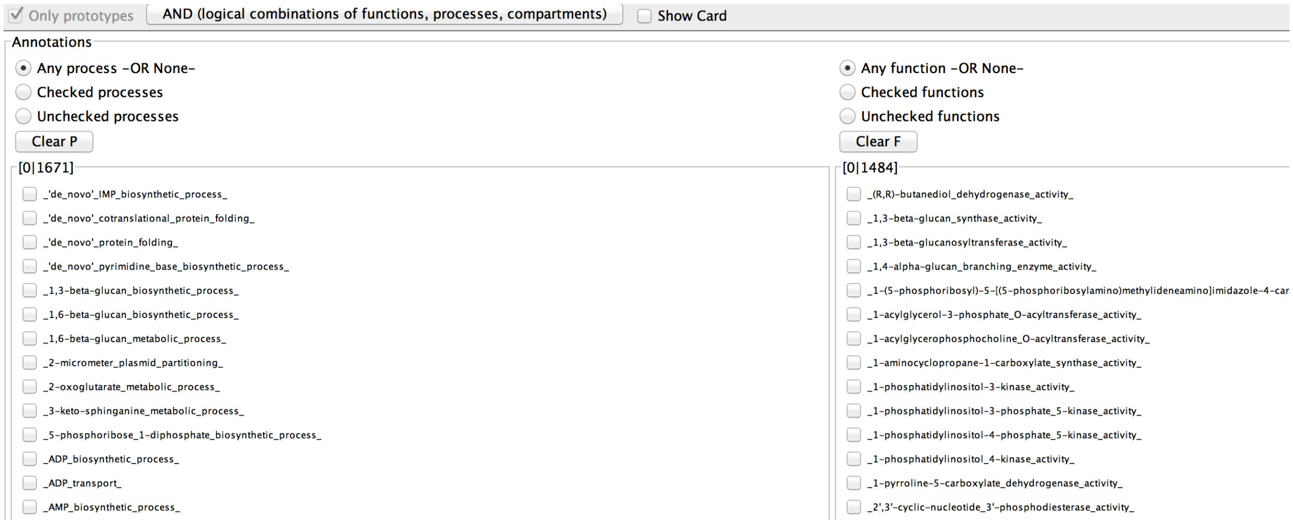

S13 Figure: Part of **AGCT**'s annotation frame, displaying all the possible tags relating a gene to biological process, function or compartment (for YCC data).

correction. The user can specify a substring of tags, in which χ^2 aggregates all tags that match the pattern. Finally, s/he can specify, by the token "All_CHI2", that χ^2 tables be built for *each* tag and *each* cluster in a clustering result. The p-values are then corrected with the Benjamini-Hochberg multiple testing procedure [24]. For example, in S13 Fig., if the user gives the tag "beta-glucan", (at least) five χ^2 tables are produced for the displayed annotations: three in biological processes ("1,3-beta-glucan_biosynthetic_process", "1,6-beta-glucan_biosynthetic_process", "1,6-beta-glucan_metabolic_process") and two in biological functions ("1,3-beta-glucan_synthase_activity", "1,3-beta-glucanosyltransferase_activity").

S2 Fig. presents the summary of an exhaustive analysis of the YCC data. The file saved by **AGCT** contains five parts:

Part (a) is the unordered set of significant tags up to a predefined p-value (here, 0.01), together with, for each of them, the set of clusters to which they are significant according to the contingency S1 Table.

Part (b) is a summary of part (a) per cluster. For each cluster, it provides the set of tags that are significant up to the predefined p-value.

Part (c) is a tabulated list of tags, ordered in increasing Benjamini-Hochberg p^* value (called q^* in the original paper, eq. (1) [24]). Each row indicates the Biological (P)rocess, (F)unction or (C)ompartment, the clustering number (in case the user has performed several clusterings), the cluster number in the given clustering, the limit p and p^* values, and the contingency table (following S1 Table and in the order YY, YN, NY, and NN).

Part (d) is a summary of part (b), indicating for each cluster the set of tags with p^* not exceeding a predefined value (here, 0.01), with, for each of them a symbol "+", "-" or "/" indicating respectively whether YY/NY $>$ YN/NN (the tag proportion among genes is larger inside the cluster compared to outside), YY/NY $<$ YN/NN (the tag proportion is smaller inside the cluster compared to outside), or YY/NY = YN/NN (the tag proportion is the same inside the cluster and outside).

Part (e) provides the data to plot the cumulative distribution of tags having p^*-value not exceeding some x value. S14 Fig. is an example plot for the data in S2 Fig., showing that more than 1 ‰ of the tags have a p^*-value below 10^{-10}.

(a)

```
All significant Chi-square tests (p_limit = 0.01)

String _(R,R)-butanediol_dehydrogenase_activity_ (F:) :
 cluster 2 : chi = 20.413827877304627 ; p-value = 6.237744711862846E-6

String _1,4-alpha-glucan_branching_enzyme_activity_ (F:) :
 cluster 2 : chi = 20.413827877304627 ; p-value = 6.237744711862846E-6

[...]
```

(b)

```
 - Summary (tags by cluster with p-value < p_limit)
 Cluster 0 : _ATPase_activity_; _DNA_binding_;
_P-P-bond-hydrolysis-driven_protein_transmembrane_transporter_activity_;

[...]
```

(c)

```
Complete and ordered list of all annotation tags, given as: Type Tag Clustering Cluster P-value P*-value [Chi2 table]
C: _cytosolic_small_ribosomal_subunit_ 0 2 1.822657302113711E-122 2.020962416583683E-118 [19.0, 31.0, 54.0, 6074.0]
F: _structural_constituent_of_ribosome_ 0 2 2.223586632169502E-121 1.2327564288747718E-117[37.0, 152.0, 36.0, 5953.0]
P: _translation_ 0 2 1.277920662259845E-94 4.7231947677123867E-91 [41.0, 249.0, 32.0, 5856.0]

[...]
```

(d)

```
Set of tags for each cluster, having P*-Values <= Satistics.LIMIT_P (0.01)
Cluster 0:

Annotation F:
- _structural_constituent_of_ribosome_ 2.077086865434843E-19
+ _transcription_factor_activity_ 6.999589848345787E-8
- _oxidoreductase_activity_ 3.9550272483544114E-7
+ _DNA_binding_ 1.0550935983963963E-4
- _catalytic_activity_ 2.3250016580073497E-4
- _endopeptidase_activity_ 3.085254206967368E-4
- _hydrogen_ion_transmembrane_transporter_activity_ 6.957390424880312E-4
+ _specific_RNA_polymerase_II_transcription_factor_activity_ 8.042338763387818E-4
- _proton-transporting_ATPase_activity,_rotational_mechanism_ 8.042338763387818E-4
- _ATPase_activity_ 0.0021760696729845003
+ _zinc_ion_binding_ 0.0021889734844762134
- _lyase_activity_ 0.002883349228454611
- _aminoacyl-tRNA_ligase_activity_ 0.003248499199051523
+ _nucleic_acid_binding_ 0.0038232265355304467
- _unfolded_protein_binding_ 0.004015300315751953
- _threonine-type_endopeptidase_activity_ 0.0062216942454285
- _hydrogen_ion_transporting_ATP_synthase_activity,_rotational_mechanism_ 0.0062216942454285
+ _transcription_activator_activity_ 0.007887973849842531

[...]
```

(e)

```
Cumulative plot (x,y), in which x=P*-value and y=%tags having P*-value <= x
2.020962416583683E-118 9.018759018759019E-5
1.2327564288747718E-117 1.8037518037518038E-4
4.7231947677123867E-91 2.7056277056277056E-4
4.38877526180365E-89 3.6075036075036075E-4
9.515640089297444E-86 4.5093795093795094E-4
2.783919028333974E-83 5.411255411255411E-4

[...]
```

S2 Table: Example of exhaustive analysis performed with token "All_CHI2" on YCC data, for $k = 3$-Means clustering. The saved file (> 1 Mb) contains five distinctive parts (a-e) (see the explanation in Subsection 3.4).

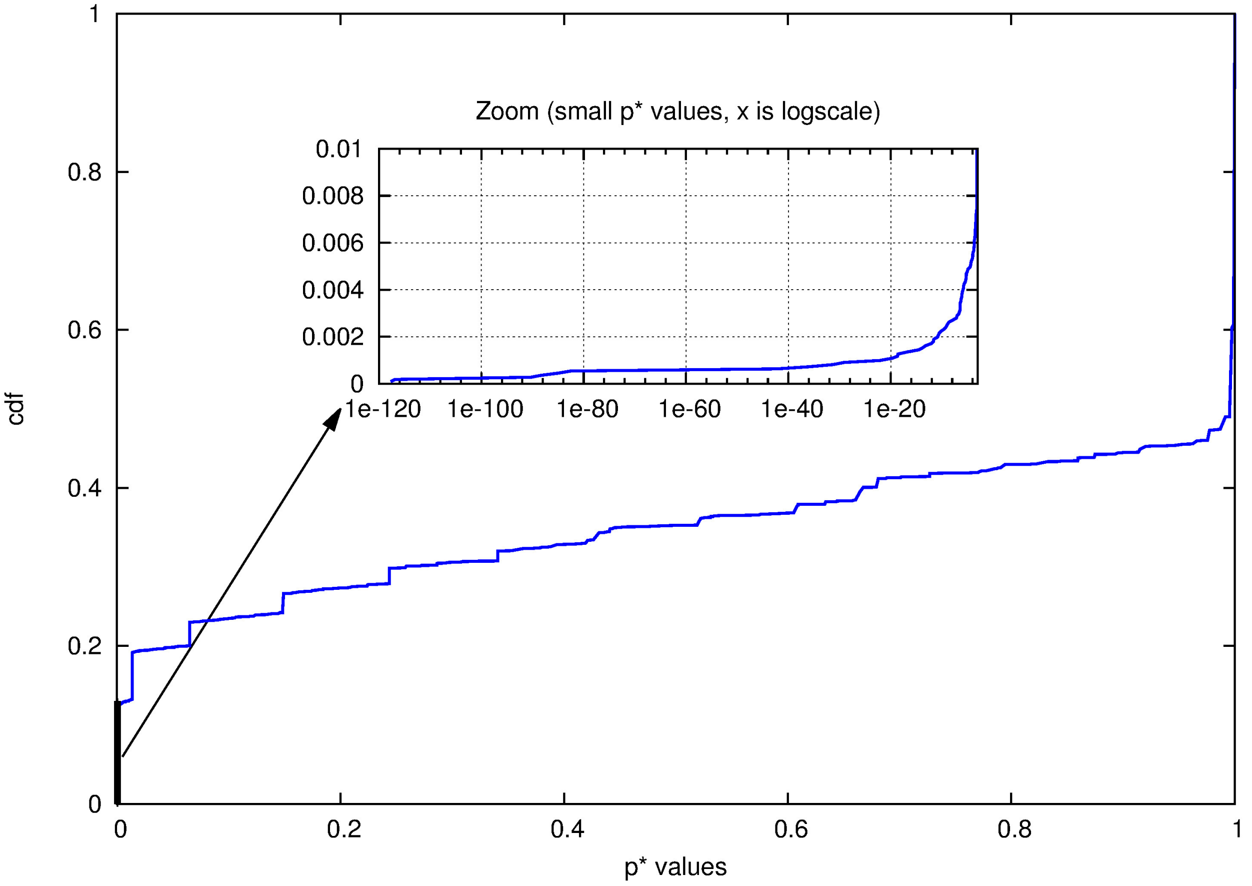

S14 Figure: Cumulative distribution function (cdf) for YCC significant tags corresponding to part (e) in S2 Table (see the explanation in Subsection 3.4).

References

[1] Roweis, S. & Saul, L. Non-linear dimensionality reduction by locally linear embedding. *Science* **290**, 2323–2326 (2000).

[2] Nock, R., Sebban, M. & Bernard, D. A simple locally adaptive nearest neighbor rule with application to pollution forecasting. *International Journal on Pattern Recognition and Artificial Intelligence* **17**, 1–14 (2003).

[3] Meilă, M. & Shi, J. Learning segmentation by random walks. In Dietterich, T. G., Becker, S. & Ghahramani, Z. (eds.) *Advances in Neural Information Processing Systems*14* (MIT Press, 2001).

[4] Sinclair, A. & Jerrum, M. Approximate counting, uniform generation and rapidly mixing Markov chains. *Information and Computation* **82**, 93–133 (1989).

[5] Shi, J. & Malik, J. Normalized cuts and image segmentation. *IEEE Transactions on Pattern Analysis and Machine Intelligence* **22**, 888–905 (2000).

[6] Krzakala, F. *et al.* Spectral redemption in clustering sparse networks. *Proc. National Academy of Sciences USA* **110**, 20935–20940 (2013).

[7] Saade, A., Krzakala, F. & Zdeborova, L. Spectral clustering of graphs with the Bethe Hessian. In *Advances in Neural Information Processing Systems*27* (Omnipress, 2014).

[8] Zass, R. & Shashua, A. Doubly-stochastic normalization for spectral clustering. In *Advances in Neural Information Processing Systems*19* (2006).

[9] Sinkhorn, R. A relationship between arbitrary positive matrices and doubly stochastic matrices. *Annals of Mathematical Statistics* **35**, 876–879 (1964).

[10] Polouliakh, N., Nock, R., Nielsen, F. & Kitano, H. G-Protein Coupled Receptor Signaling Architecture of Mammalian Immune Cells. *PLoS ONE* **4** (2009).

[11] Arthur, D. & Vassilvitskii, S. k-means++ : the advantages of careful seeding. In *Proc. of the 19 th ACM-SIAM Symposium on Discrete Algorithms*, 1027 – 1035 (2007).

[12] Nock, R., Luosto, P. & Kivinen, J. Mixed Bregman Clustering with Approximation Guarantees. In *Machine Learning and Knowledge Discovery in Databases, European Conference*, 154–169 (Springer Verlag LNCS, 2008).

[13] Bregman, L. M. The relaxation method of finding the common point of convex sets and its application to the solution of problems in convex programming. *USSR Comp. Math. and Math. Phys.* **7**, 200–217 (1967).

[14] Banerjee, A., Merugu, S., Dhillon, I. & Ghosh, J. Clustering with Bregman divergences. *Journal of Machine Learning Research* **6**, 1705–1749 (2005).

[15] Dempster, A. P., Laird, N. M. & Rubin, D. B. Maximum likelihood from incomplete data via the EM algorithm. *Journal of the Royal Statistical Society B* **39**, 1–38 (1977).

[16] Zass, R. & Shashua, A. A unifying approach to hard and probabilistic clustering. In *Proc. of the 11 th IEEE International Conference on Computer Vision*, 294–301 (2005).

[17] Frey, B.-J. & Dueck, D. Clustering by passing messages between data points. *Science* **315**, 972–976 (2007).

[18] Lee, D.-D. & Seung, H.-S. Algorithms for non-negative matrix factorization. In *Advances in Neural Information Processing Systems*13*, 556–562 (MIT press, 2000).

[19] Kim, P.-M. & Tidor, B. Subsystem identification through dimensionality reduction of large-scale gene expression data. *Genome Research* **13**, 1706–1718 (2003).

[20] Delaunay, B. Sur la sphère vide. *Izvestia Akademii Nauk SSSR, Otdelenie Matematicheskikh i Estestvennykh Nauk* **7**, 793–800 (1934).

[21] Levina, E. & Bickel, P.-J. Maximum likelihood estimation of intrinsic dimension. In *Advances in Neural Information Processing Systems*17* (2004).

[22] MacKay, D.-J.-C. & Ghahramani, Z. Comments on 'maximum likelihood estimation of intrinsic dimension' by E. Levina and P. Bickel (2005). URL `http://www.inference.phy.cam.ac.uk/mackay/dimension`.

[23] Das Gupta, M. & Huang, T.-S. Regularized maximum likelihood for intrinsic dimension estimation. In *Proc. of the 26 th Conference on Uncertainty in Artificial Intelligence,*, 220–227 (2010).

[24] Benjamini, Y. & Hochberg, Y. Controlling the false discovery rate: a practical and powerful approach to multiple testing. *Journal of the Royal Statistical Society* **57**, 289–300 (1995).
