# Supplementary material for: A Geometric Clustering Tool (AGCT) to robustly unravel the inner cluster structures of time-series gene expressions
Source: PLoS One. 2020 Jul 6;15(7):e0233755. doi: 10.1371/journal.pone.0233755 (PMC7337352; doi:10.1371/journal.pone.0233755)
Supplement: S2 File — (PDF) [file pone.0233755.s002.pdf]

AGCT Tutorial

Step-by-step introduction

AGCT dataset

```
Species Yeast.txt
E (entity)          1         Cell1
EG (entity grouping)          1         1
T (time)            36        1         2         3         4         5         6         7         8         9         10        11
R (replicate)       1
Data     564
10001_at            1.994324565         1.805780292         1.173666358         1.147356629         0.940516353         0.930283248
10003_f_at          22.51645851         11.07745647         19.84449577         17.49943733         17.20314407         18.38671112
10005_at            2.220204353         1.906358242         2.366628885         1.8436445           1.734006763         1.860566497
10006_at            0.657207727         0.635838151         0.656072617         0.905511856         1.127946138         1.001089334
10010_at            29.91146469         26.66589737         32.16685486         22.47244263         15.67227936         10.69390011
10011_at            0.264472187         0.116763011         0.17026107          0.15185602          0.14927049          0.077342048
10012_at            1.585698009         3.213872671         3.719636917         3.665916681         4.057239056         4.177559853
10013_at            0.700340509         0.897109807         1.055618644         0.942632198         0.940516353         1         0.9
10015_at            4.947786808         9.030056953         14.13961411         9.087739944         9.347924232         6.636165619
10016_at            3.312145233         2.530635595         2.947786808         3.011248827         2.66554451          2.198257208
10018_at            43.29738998         25.93988419         41.94778824         43.53430939         36.99775696         33.83224487
10019_at            40.52213287         39.93179321         34.62996674         26.75140572         24.3254776          23.30827904
10021_at            0.60839951          0.893641651         0.679909229         0.782902122         0.912458003         0.751634002
10026_at            1.220204353         2.546820879         1.316685557         2.213723421         2.059483767         1.745097995
10027_at            6.491487503         10.24624252         7.511918068         8.645668983         8.375982285         7.60566473
10028_at            0.648127139         0.582658947         0.740068078         0.373453349         0.718294084         0.698257089
10029_at            2.457434654         2.38959527          2.039727449         1.480315089         0.854096532         0.957516372
10031_at            1.404086232         1.365317822         1.769580007         1.519685149         1.904601574         1.624183059
10032_at            0.936435878         1.253179193         1.657207727         1.600674987         1.722783446         1.318082809
10036_at            1.644721866         1.964161754         2.013620853         2.424072027         2.099887848         2.165577412
10040_at            1.405221343         1.531791925         1.38138473          1.394825697         1.564534187         1.310457587
10043_at            9.01816082          19.66589546         13.46765041         11.17660332         11.27833939         9.603485107
10044_at            2.707150936         1.81734097          1.460839987         1.646794081         1.404040456         1.36601305
10045_at            2.922815084         8.425433159         45.61407471         69.77278137         60.65769196         49.42483902
10046_at            0.124858119         0.171098262         0.246311024         0.269966245         0.407407403         0.352941185
10048_at            1.293984175         1.376878619         1.248581171         1.933633208         1.758698106         2.035947561
10049_at            1.916004539         1.108670473         0.555051088         0.73453325          0.59932667          0.636165619
10050_at            3.827468872         4.931791782         3.595913649         5.411698818         2.720538855         5.583877563
10051_at            1.748013616         2.60346818          1.782065868         2.811023712         2.499439001         2.824618578
10052_at            1.860385895         1.643930554         1.703745842         1.830146194         1.493827343         1.503268003
10053_at            8.368899345         4.337572098         9.069239616         7.421822071         9.430976868         6.896513939
10054_at            23.40522194         23.17687798         29.45857048         19.60517502         17.40067482         11.58060932
10055_at            8.177071571         4.02543354          2.634506226         2.452193499         2.268238068         1.979302764
10056_at            2.304199696         5.123699665         3.113507271         4.482564926         4.317620754         3.967320442
10058_at            2.39160037          5.872832298         6.331441402         7.707536697         8.800224304         8.18954277
10059_at            4.858115673         4.272832394         3.374574184         4.865016937         5.373737335         4.647058964
10060_at            3.417707205         12.13410378         9.771850586         11.81439877         9.334456444         8.826797485
10061_at            42.83314514         45.29364014         31.3689003          38.09674072         32.96408844         31.83660316
10068_i_at          14.54370117         7.721387386         13.72077274         16.03599548         18.19079781         15.3932457
10069_f_at          9.527810097         6.567629814         11.72304249         11.0731163          10.35241413         8.009803772
10071_at            0.402951181         0.436994195         0.712826312         0.512935877         0.586980939         0.412854046
10072_at            0.473325789         1.161849737         1.089670897         1.17660296          1.125701547         1.058823466
```

Species - specify Gene Ontology File
E (entity) - number of sets (cells here) to analyze
EG (entity grouping) - number of subgroups (optional, can be ignored)
T (time) - total number of timestamps, followed by a list of stamps
R (replicate) - number of replicates
Data - number of entities in the list (number of probes here)

List of IDs followed by values (in this case: E*EG*T*R=36 tags)
10001_at 1.99432457 1.80578029 1.17366636 1.14735663 ...n=36

Launch AGCT and loading a dataset

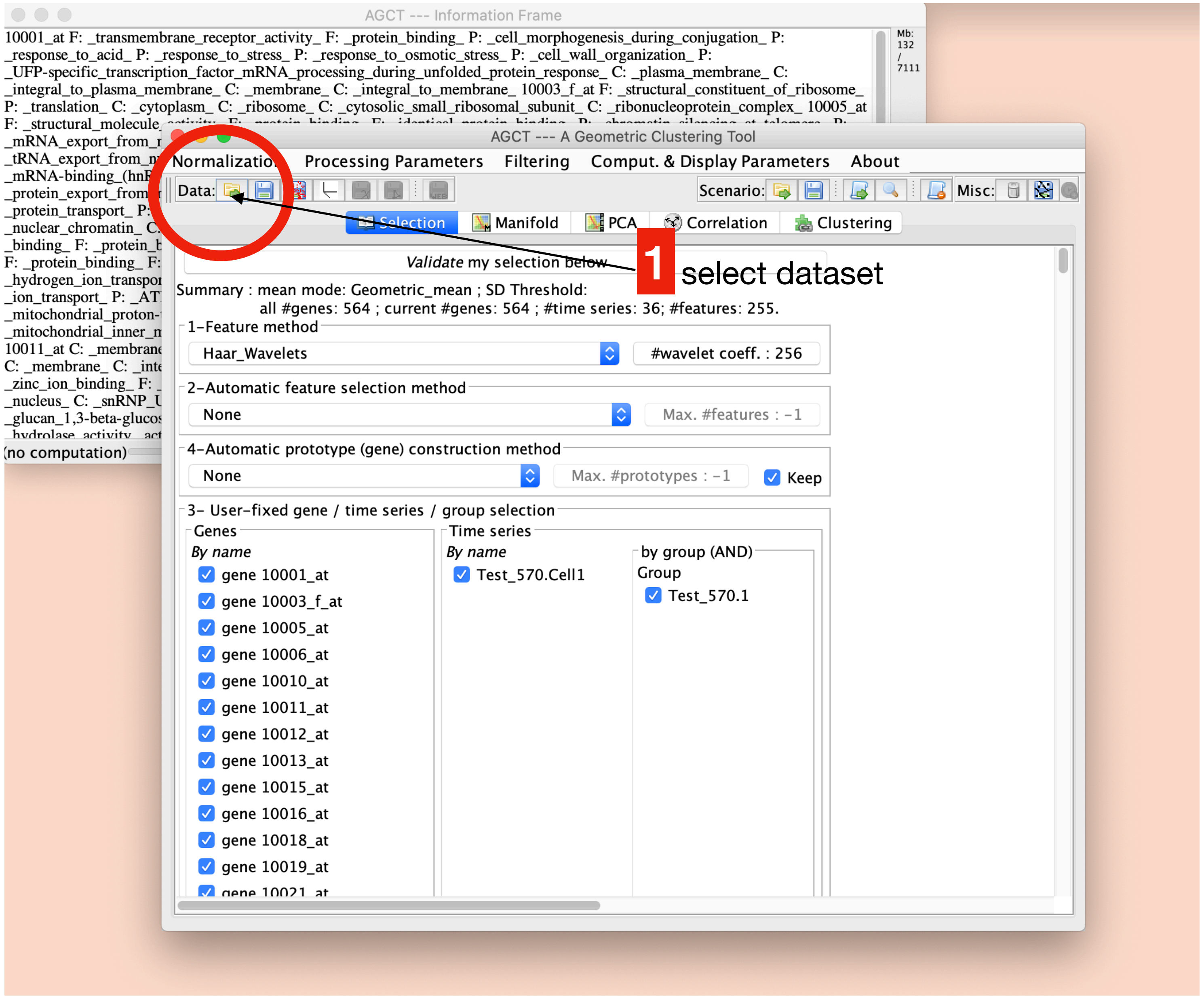

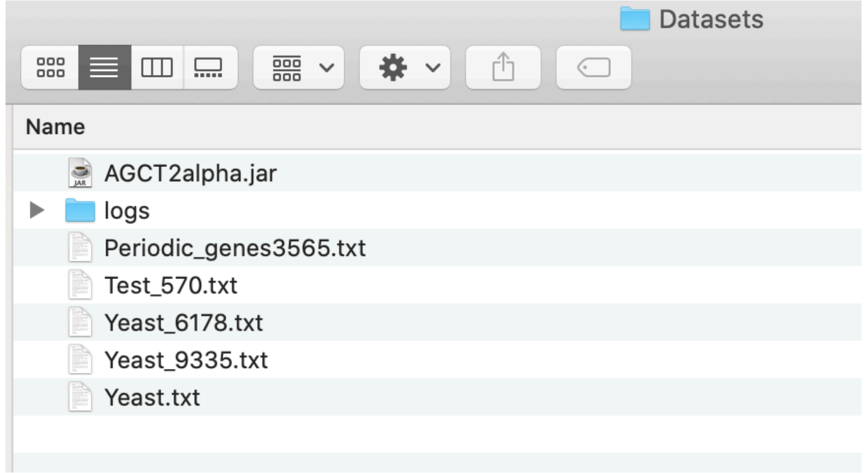

java -d64 -Xmx8000m -jar AGCT2alpha.jar

Execute AGCT on dataset

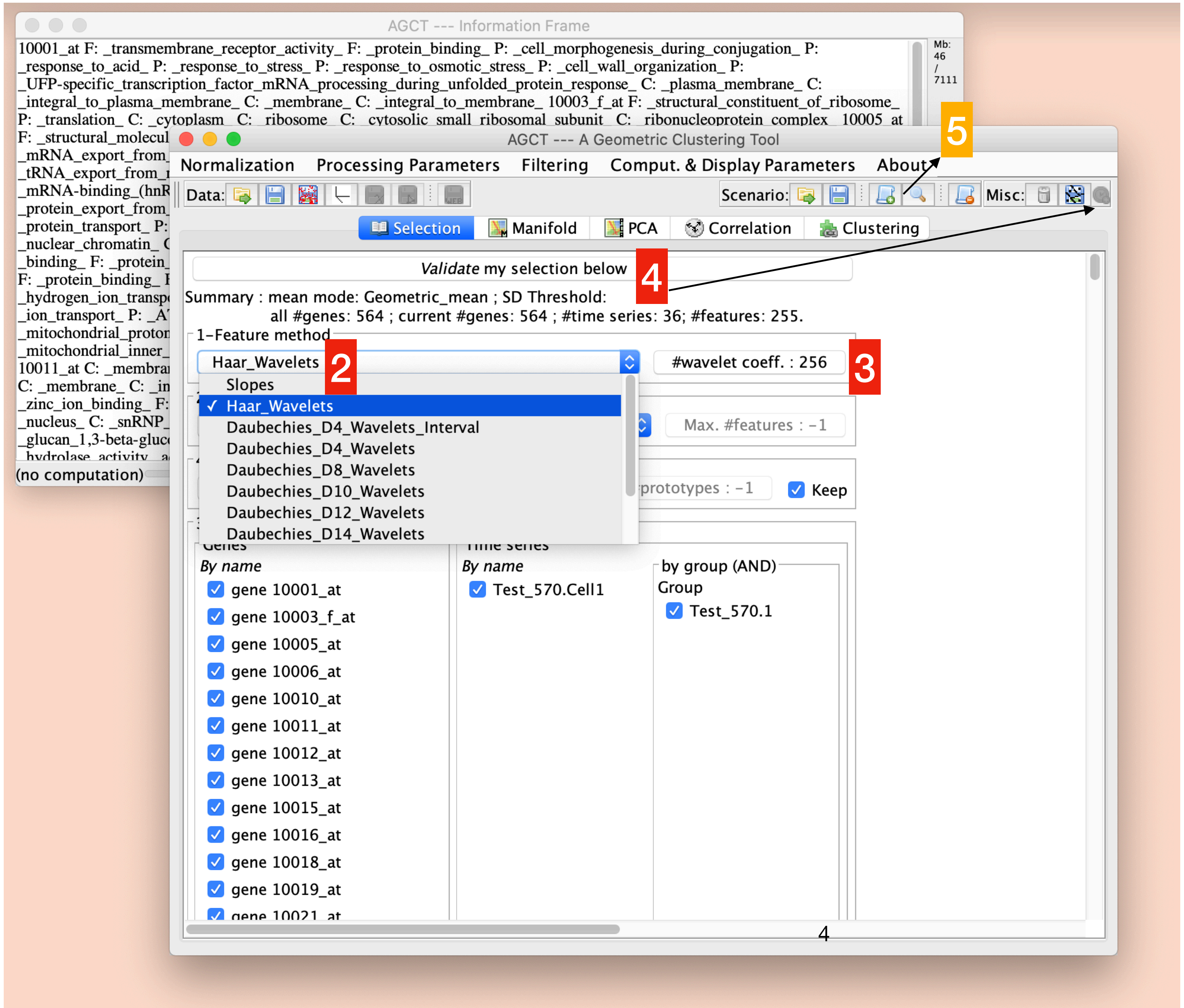

2 Select feature compression method

3 Select waveletloefficient (if used)

4 Execute job

5 Scenario automatically recorded before manually stopped

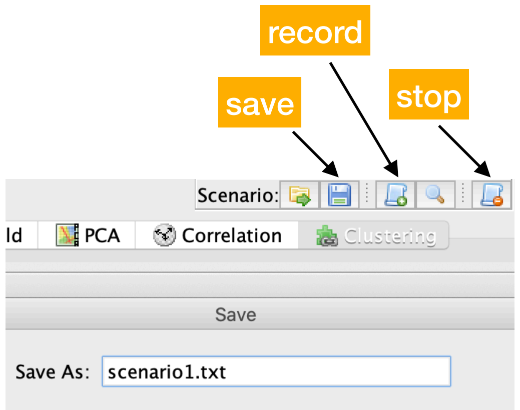

The scenario can be saved to manifold calculation.
Next time, to load **your recorded scenario** you have, first, **to stop the automatic recording** scenario button.

Visualization Manifold/PCA

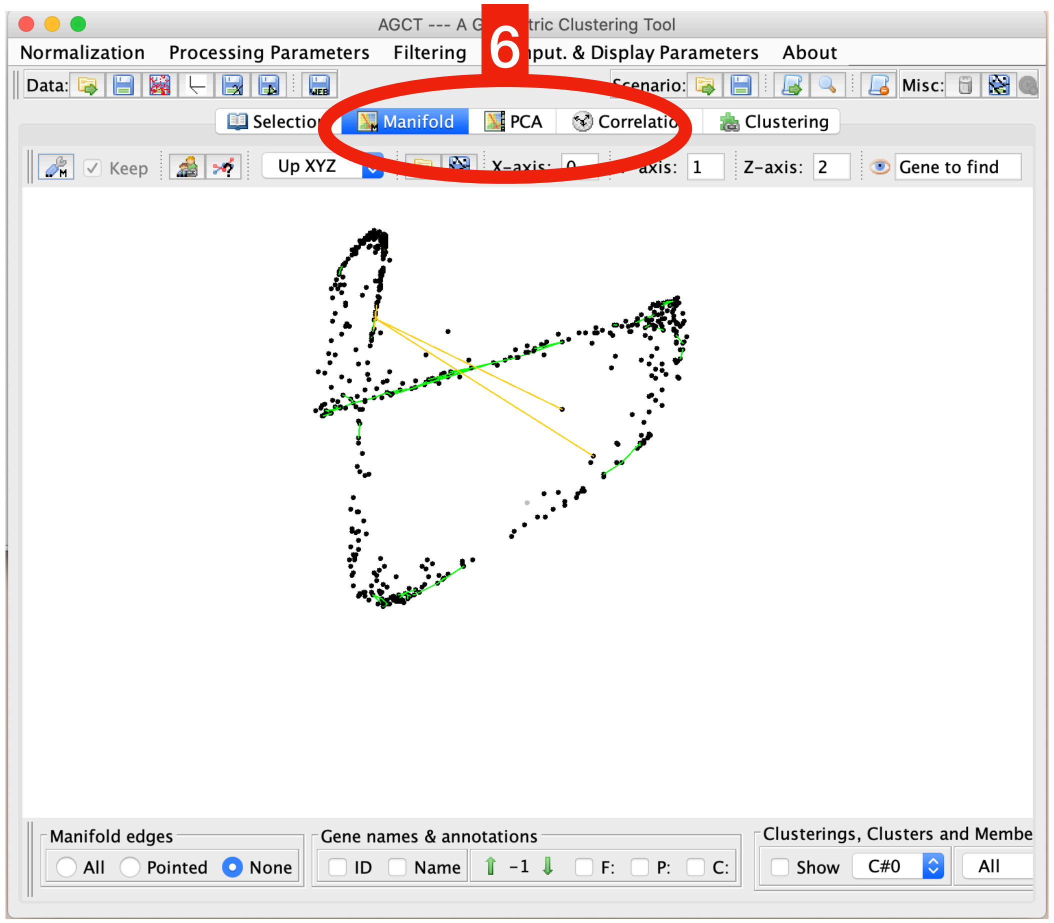

Clustering method selection

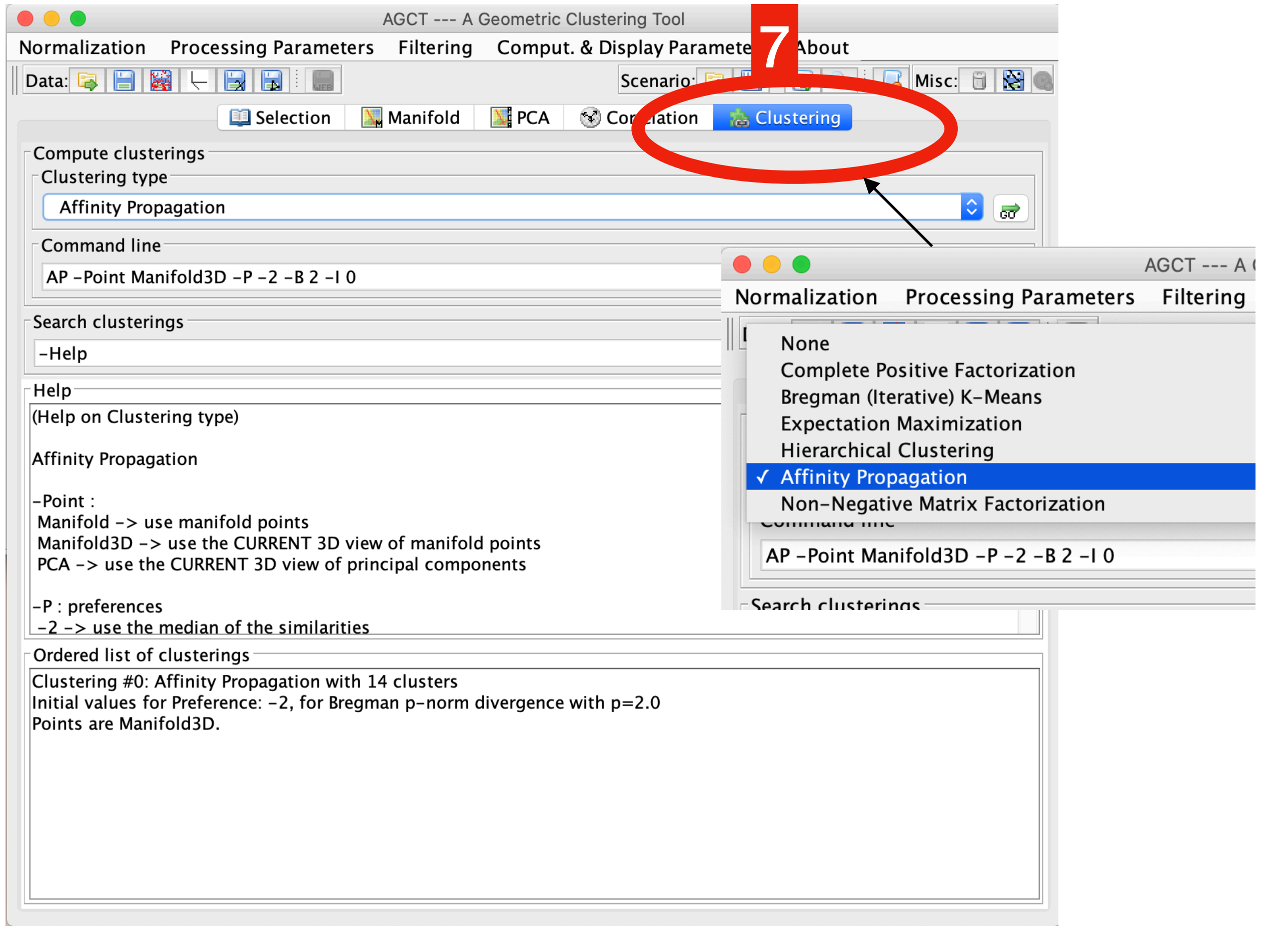

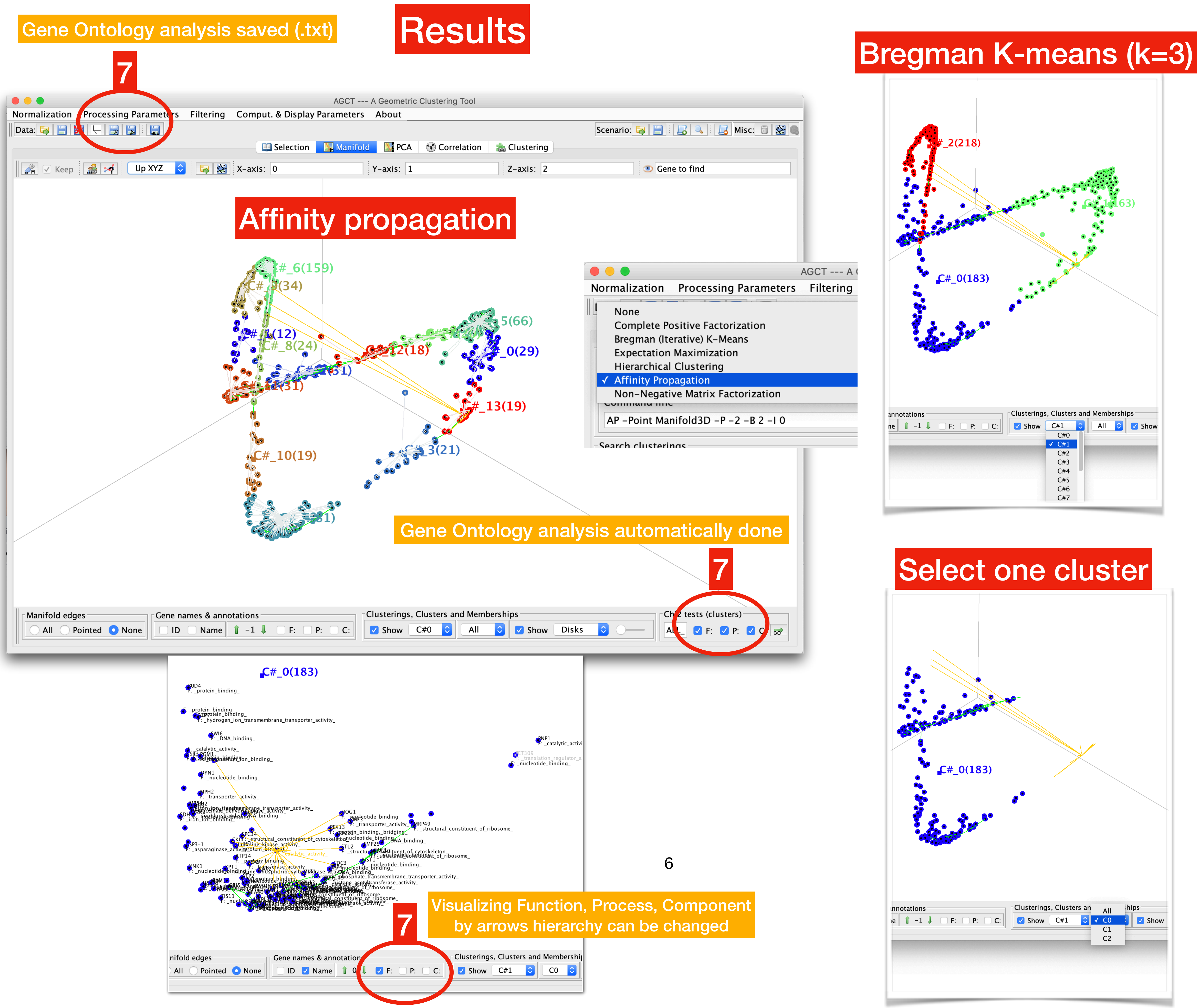
Results
Gene Ontology analysis saved (.txt)
7
AGCT --- A Geometric Clustering Tool
Normalization
Processing Parameters
Filtering
Comput. & Display Parameters
About
Selection
Manifold
PCA
Correlation
Clustering
Affinity propagation
None
Complete Positive Factorization
Bregman (Iterative) K-Means
Expectation Maximization
Hierarchical Clustering
Affinity Propagation
Non-Negative Matrix Factorization
AP -Point Manifold3D -P -2 -B 2 -I 0
Gene Ontology analysis automatically done
Manifold edges
Gene names & annotations
Clusterings, Clusters and Memberships
C#_0(183)
Visualizing Function, Process, Component by arrows hierarchy can be changed
Bregman K-means (k=3)
Select one cluster

Loading genes to highlight from separate file

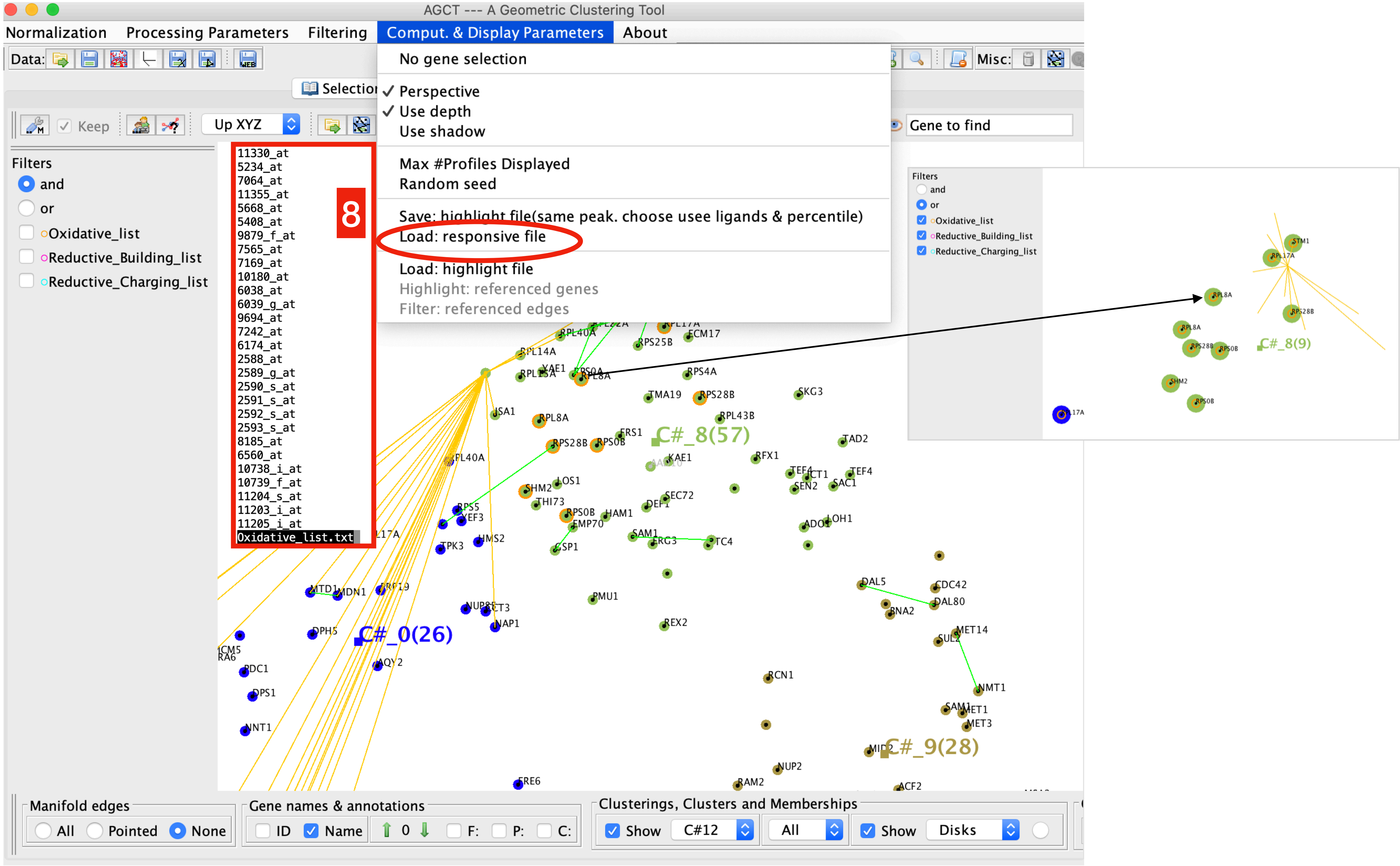

Cluster profiles

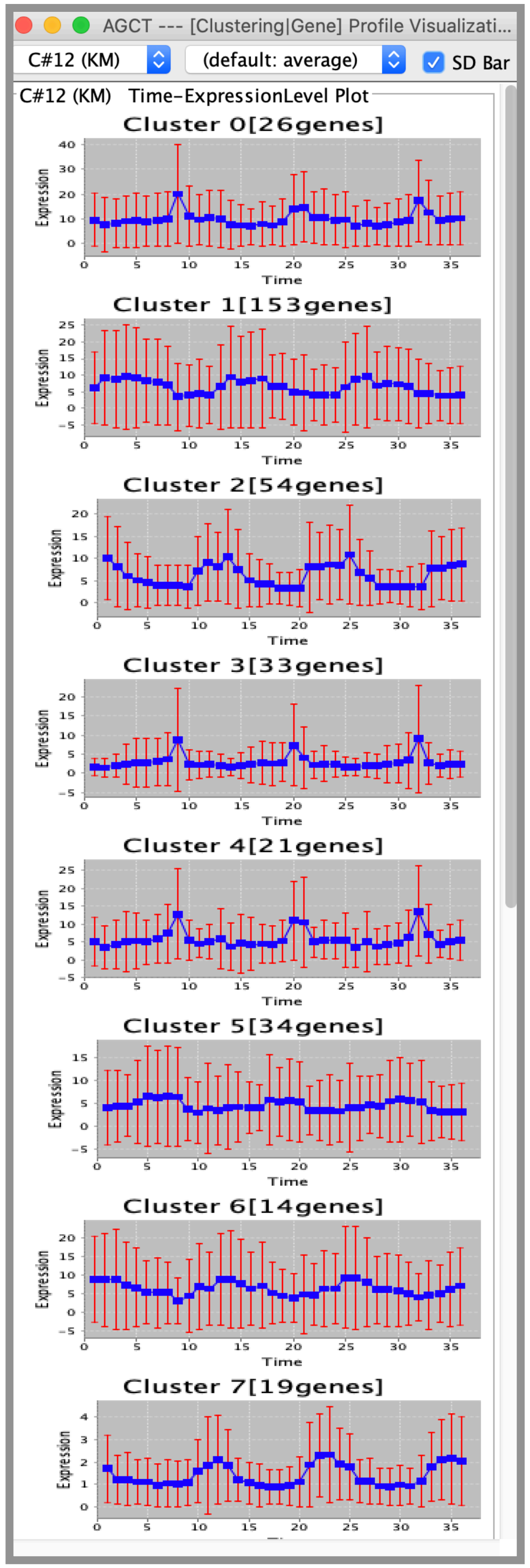

Cluster saving

9

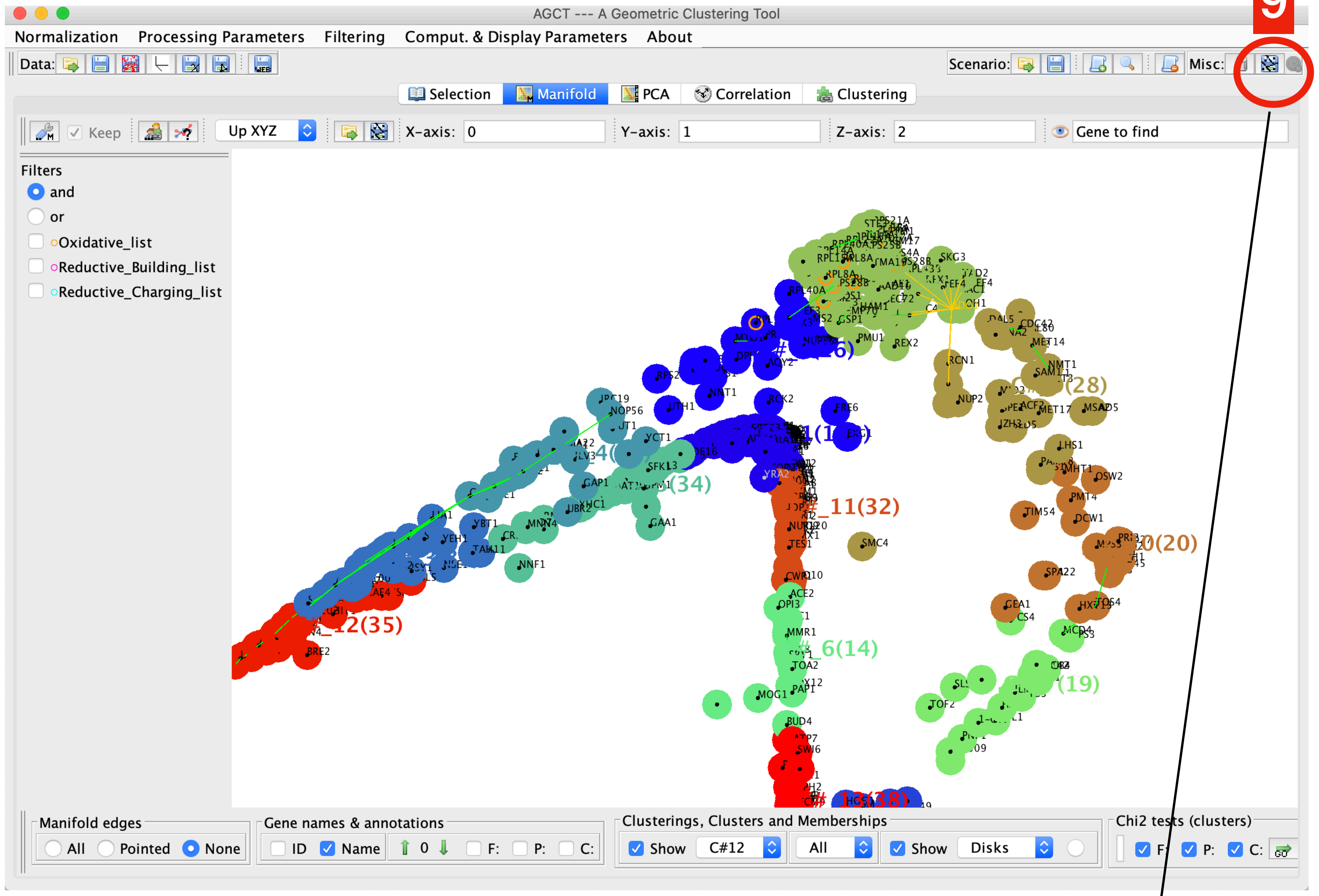


```
AGCT2alpha.jar                    Test_570_cluster_4.txt
Periodic_genes3565.txt            Test_570_cluster_5.txt
Test_570.txt                      Test_570_cluster_6.txt
Test_570_clusterProfile.txt       Test_570_cluster_7.txt
Test_570_cluster_1.txt            Test_570_cluster_8.txt
Test_570_cluster_10.txt           Test_570_cluster_9.txt
Test_570_cluster_11.txt           Test_570_grouping.txt
Test_570_cluster_12.txt           Yeast.txt
Test_570_cluster_13.txt           Yeast_6178.txt
Test_570_cluster_14.txt           Yeast_9335.txt
Test_570_cluster_2.txt            logs
Test_570_cluster_3.txt
```
