# Supplementary figures and images for: A Geometric Clustering Tool (AGCT) to robustly unravel the inner cluster structures of time-series gene expressions
Source: PLoS One. 2020 Jul 6;15(7):e0233755. doi: 10.1371/journal.pone.0233755 (PMC7337352; doi:10.1371/journal.pone.0233755)

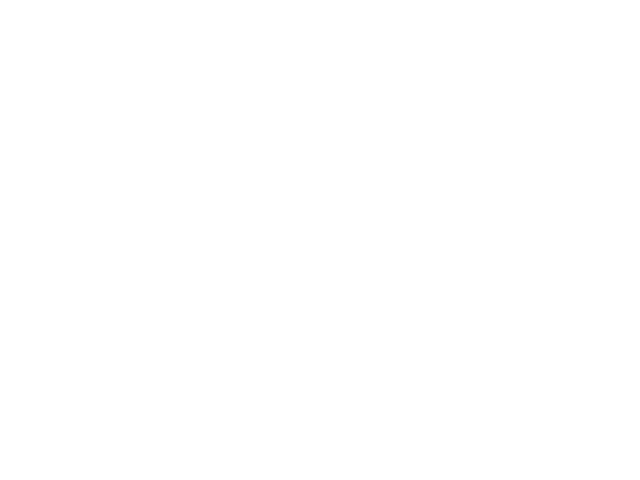

Supplement: S4 File — (ZIP) [file pone.0233755.s004.zip › ScriptFiles/OutputFiles/t-SNE of 9335 Periodic Genes Manifold Data (K-means = 4).png]

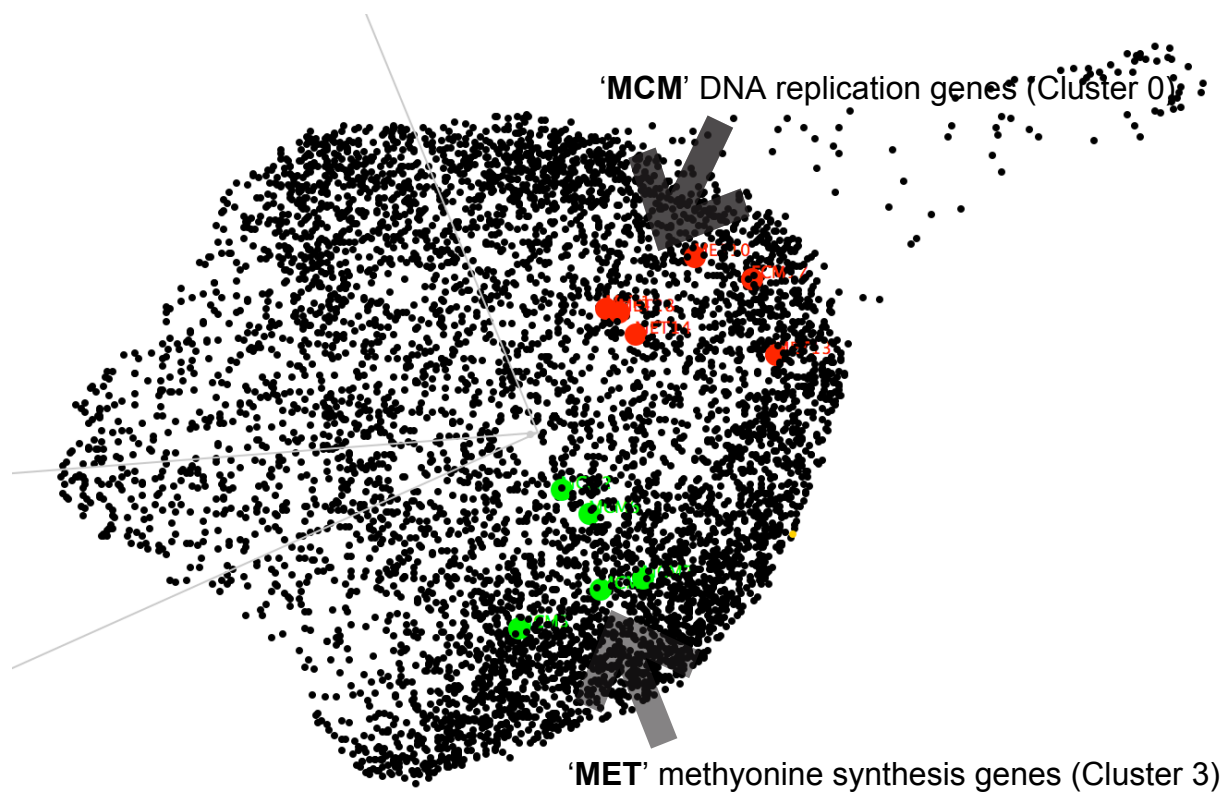

**Figure S19:** View of 'MCM' DNA replication genes and 'MET' methionine synthesis genes on the manifold.

Supplement: S5 Fig — (PDF) [file pone.0233755.s014.pdf]

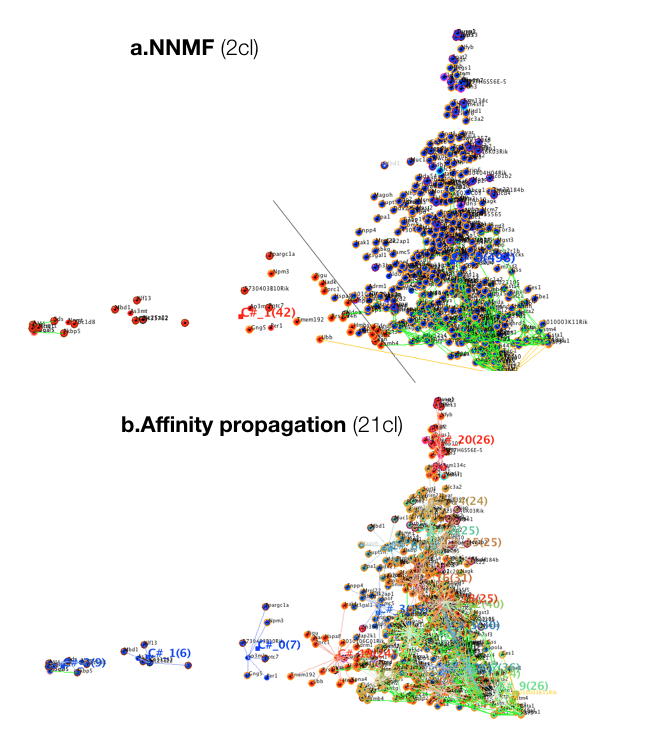

Supplement: S6 Fig — (a) NNMF (k = 2), (b) Affinity propagation (k = 21), (c) Delaunay Triangulation on top co-regulated genes (p≤0.001, green edges). (PNG) [file pone.0233755.s015.png]
