# Supplementary material for: A Geometric Clustering Tool (AGCT) to robustly unravel the inner cluster structures of time-series gene expressions
Source: PLoS One. 2020 Jul 6;15(7):e0233755. doi: 10.1371/journal.pone.0233755 (PMC7337352; doi:10.1371/journal.pone.0233755)
Supplement: S1 Table — (DOCX) [file pone.0233755.s007.docx]

**S3 Table**: Major GO tags that define four clusters of Tu et al dataset (9,334G), with p-value < 10E−3. P – GO biological process, F – molecular function, C – cellular component.

| Reductive/Charging Cluster_2 4,571G [M-G1(70%) ,M(43%), G2(40%)] | | |
| --- | --- | --- |
| P | 1.34E-743.75E-622.57E-355.81E-177.34E-174.01E-159.33E-148.15E-118.34E-111.71E-09 | _ribosome_biogenesis__mitochondrial_translation__rRNA_processing__ribosomal_large_subunit_biogenesis__endonucleolytic_cleavage_to_generate_mature_5'-end_of_SSU-rRNA_from_(SSU-rRNA,_5.8S_rRNA,_LSU-rRNA)__endonucleolytic_cleavage_in_5'-ETS_of_tricistronic_rRNA_transcript_(SSU-rRNA,_5.8S_rRNA,_LSU-rRNA)__endonucleolytic_cleavage_in_ITS1_to_separate_SSU-rRNA_from_5.8S_rRNA_and_LSU-rRNA_from_tricistronic_rRNA_transcript_(SSU-rRNA,_5.8S_rRNA,_LSU-rRNA)__tRNA_methylation__aerobic_respiration__transposition_ |
| F | 3.35E-10  1.06E-09  1.05E-08  1.37E-08  4.78E-08  7.74E-08  3.52E-07  1.22E-06  1.83E-06  1.94E-06 | _snoRNA_binding_  _DNA-directed_DNA_polymerase_activity_  _RNA-directed_DNA_polymerase_activity_  _aspartic-type_endopeptidase_activity_  _structural_constituent_of_ribosome_  _ribonuclease_H_activity_  _peptidase_activity_  _ATP-dependent_RNA_helicase_activity_  _nucleotidyltransferase_activity_  _hydrogen_ion_transporting_ATP_synthase_activity,_rotational_mechanism_ |
| C | 2.20E-57  1.00E-54  2.62E-40  5.15E-35  7.87E-25  2.60E-24  9.65E-24  3.74E-14  5.56E-13 | _mitochondrion_  _nucleolus_  _mitochondrial_large_ribosomal_subunit_  _mitochondrial_inner_membrane_  _preribosome,_large_subunit_precursor_  _mitochondrial_matrix_  _mitochondrial_small_ribosomal_subunit_  _mitochondrial_intermembrane_space_  _nucleus_ |
| Oxidative Cluster_1 2,590G [G1(59%), S(37%), G2,M] | | |
| P | 0  0  0  0  0  1.27E-43  4.28E-40  2.15E-14  1.69E-08  3.72E-07 | _DNA_recombination_  _transposition_  _proteolysis_  _DNA_integration_  _viral_procapsid_maturation_  _RNA-dependent_DNA_replication_  _amino_acid_transport_  _intron_homing_  _aerobic_respiration_  _movement_of_group_I_intron_ |
| F | 0  0  0  0  0  0  0  0  0  5.95E-302 | _magnesium_ion_binding_  _aspartic-type_endopeptidase_activity_  _RNA-directed_DNA_polymerase_activity_  _ribonuclease_H_activity_  _nuclease_activity_  _DNA-directed_DNA_polymerase_activity_  _endonuclease_activity_  _nucleotidyltransferase_activity_  _peptidase_activity_  _nucleic_acid_binding__ |
| C | 2.07E-36  9.52E-28  9.20E-06  7.05E-05  2.60E-04  3.19E-04  4.26E-04  1.E-03  3.E-03  5.E-03 | _nucleus_  _cytoplasm_  _intracellular_  _plasma_membrane_  _ribosome_  _mitochondrion_  _ribonucleoprotein_complex_  _mitochondrial_respiratory_chain_  _nucleolus_  _mitochondrial_respiratory_chain_complex_IV_ |
| Reductive/Building Cluster_0 1,674G [S(41%),G2(30%)] | | |
| P | 3.09E-53  1.32E-45  1.62E-28  2.59E-27  6.62E-27  8.93E-26  1.68E-25  2.14E-21  5.00E-14  6.28E-12  5.74E-09 | _translation_  _ribosome_biogenesis_  _viral_procapsid_maturation_  _DNA_integration_  _rRNA_processing_  _transposition_  _DNA_recombination_  _proteolysis_  _mitochondrial_translation_  _maturation_of_SSU-rRNA_from_tricistronic_rRNA_transcript_(SSU-rRNA,_5.8S_rRNA,_LSU-rRNA)_  _endonucleolytic_cleavage_in_ITS1_to_separate_SSU-rRNA_from_5.8S_rRNA_and_LSU-rRNA_from_tricistronic_rRNA_transcript_(SSU-rRNA,_5.8S_rRNA,_LSU-rRNA)_ |
| F | 1.91E-64  1.55E-52  1.28E-38  1.29E-35  1.28E-34  4.17E-32  5.90E-32  1.05E-30  1.10E-30  2.79E-30 | _RNA_binding_  _structural_constituent_of_ribosome_  _nucleic_acid_binding_  _nuclease_activity_  _endonuclease_activity_  _nucleotidyltransferase_activity_  _RNA-directed_DNA_polymerase_activity_  _aspartic-type_endopeptidase_activity_  _DNA-directed_DNA_polymerase_activity_  _ribonuclease_H_activity_ |
| C | 1.72E-59  1.42E-49  1.97E-43  9.43E-33  2.09E-21  1.35E-20  3.00E-18  1.94E-16  1.21E-12  3.91E-12 | _ribonucleoprotein_complex_  _nucleus_  _ribosome_  _nucleolus_  _intracellular_  _cytosolic_large_ribosomal_subunit_  _cytoplasm_  _90S_preribosome_  _cytosolic_small_ribosomal_subunit_  _small-subunit_processome_ |
| Aperiodic Cluster_3 499G | | |
| P | 1.03E-60  1.35E-19  4.23E-19  4.44E-19  1.12E-15  1.24E-15  7.52E-14  1.31E-13  2.52E-10  7.40E-09  8.45E-09 | _translation_  _DNA_integration_  _transposition_  _viral_procapsid_maturation_  _amino_acid_biosynthetic_process_  _DNA_recombination_  _rRNA_export_from_nucleus_  _proteolysis_  _DNA_replication_  _methionine_biosynthetic_process_  _methionine_metabolic_process_ |
| F | 5.61E-44  3.04E-21  4.00E-20  4.80E-19  7.13E-19  3.38E-16  6.60E-16  8.67E-14  9.77E-13  6.45E-09 | _structural_constituent_of_ribosome_  _RNA-directed_DNA_polymerase_activity_  _aspartic-type_endopeptidase_activity_  _endonuclease_activity_  _ribonuclease_H_activity_  _peptidase_activity_  _DNA-directed_DNA_polymerase_activity_  _nuclease_activity_  _nucleotidyltransferase_activity_  _translation_initiation_factor_activity_ |
| C | 6.80E-46  2.00E-43  2.21E-38  2.09E-37  1.28E-25  6.37E-08  1.37E-06  2.74E-05  4.18E-05  1.75E-04 | _cytosolic_large_ribosomal_subunit_  _ribonucleoprotein_complex_  _ribosome_  _cytosolic_small_ribosomal_subunit_  _intracellular_  _cytoplasm_  _small_ribosomal_subunit_  _endoplasmic_reticulum_membrane_  _endoplasmic_reticulum_  _90S_preribosome_ |
