# Supplementary material for: A Geometric Clustering Tool (AGCT) to robustly unravel the inner cluster structures of time-series gene expressions
Source: PLoS One. 2020 Jul 6;15(7):e0233755. doi: 10.1371/journal.pone.0233755 (PMC7337352; doi:10.1371/journal.pone.0233755)
Supplement: S2 Table — Statistics of Delaunay triangulation for YMC (a) and YCC (b), for different p-values. (PDF) [file pone.0233755.s008.pdf]

S4 Table: Statistics of Delaunay triangulation for YMC (a) and YCC (b), for different p-values.

a

#edges	2467	24674	45310
p	0.2	0.5	0.7
τ_m	0.05	0.23	0.81
τ_a	0.29	0.30	0.44
τ_f	0	0	0

b

#edges	406	16013	31357
p	0.2	0.5	0.7
τ_m	0.07	0.43	0.43
τ_a	0.72	0.64	0.73
τ_f	0	0	0
