# Supplementary material for: A Geometric Clustering Tool (AGCT) to robustly unravel the inner cluster structures of time-series gene expressions
Source: PLoS One. 2020 Jul 6;15(7):e0233755. doi: 10.1371/journal.pone.0233755 (PMC7337352; doi:10.1371/journal.pone.0233755)
Supplement: S3 Table — (DOCX) [file pone.0233755.s009.docx]

**S5 Table:** Major GO tags that define six clusters of Spellman CDC28 et al dataset (6,178G), with p-value < 10E−2. P – GO biological process, F – molecular function, C – cellular component.

| Cluster_0 1,324G [M(44%),M-G1, G2] | | |
| --- | --- | --- |
| P | 3.E-105.E-089.E-071.E-064.E-045.E-041.E-031.E-031.E-031.E-03 | _transport__ribosome_biogenesis__rRNA_processing__mitochondrial_translation__ATP_biosynthetic_process__aerobic_respiration__ion_transport__vesicle_docking_during_exocytosis__cell_cycle__endocytosis_ |
| F | 8.E-03  2.E-02  2.E-02  3.E-02  3.E-02  5.E-02  6.E-02  7.E-02  7.E-02  8.E-02  8.E-02 | _DNA_binding_  _RNA_binding_  _endopeptidase_activity_  _peptidase_activity_  _hydrogen_ion_transporting_ATP_synthase_activity,_rotational_mechanism_  _iron_ion_transmembrane_transporter_activity_  _transcription_factor_activity_  _protein_kinase_inhibitor_activity_  _succinate_dehydrogenase_(ubiquinone)_activity_  _glucan_endo-1,3-beta-D-glucosidase_activity_ |
| C | 8.43E-18  2.45E-13  3.51E-12  6.03E-11  5.72E-06  1.27E-05  5.03E-05  5.44E-05  1.08E-04  2.75E-04 | _mitochondrion_  _membrane_  _mitochondrial_inner_membrane_  _nucleus_  _integral_to_membrane_  _nucleolus_  _endosome_membrane_  _endosome_  _mitochondrial_matrix_  _vacuolar_membrane_ |
| Cluster_1 1,419G [G1(69%), M-G1] | | |
| P | 2.E-11  1.E-07  7.E-07  2.E-04  2.E-04  6.E-04  1.E-03  1.E-03  1.E-03  2.E-03 | _mitotic_sister_chromatid_cohesion_  _translation_  _DNA_replication_  _heteroduplex_formation_  _DNA_repair_  _lagging_strand_elongation_  _telomere_maintenance_via_telomerase_  _double-strand_break_repair_via_homologous_recombination_  _mismatch_repair_  _cell_cycle_ |
| F | 7.E-09  1.E-04  4.E-04  5.E-04  1.E-03  1.E-03  1.E-03  2.E-03  2.E-03  2.E-03 | _structural_constituent_of_ribosome_  _double-stranded_DNA_binding_  _RNA_binding_  _dolichyl-phosphate-mannose-protein_mannosyltransferase_activity_  _chaperone_activator_activity_  _copper_uptake_transmembrane_transporter_activity_  _aldo-keto_reductase_activity_  _mannosyltransferase_activity_  _ubiquitin-protein_ligase_activity_  _hydrolase_activity,_acting_on_glycosyl_bonds_ |
| C | 2.E-06  3.E-06  5.E-05  1.E-04  3.E-04  8.E-04  2.E-03  4.E-03  8.E-03  9.E-03 | _ribonucleoprotein_complex_  _ribosome_  _replication_fork_  _nuclear_cohesin_complex_  _condensed_nuclear_chromosome_  _intracellular_  _GINS_complex_  _cytosolic_large_ribosomal_subunit_  _alpha_DNA_polymerase:primase_complex_  _chromosome,_telomeric_region_ |
| Cluster_2 992G [M-G1(58%),G1] | | |
| P | 3.E-22  3.E-06  3.E-06  3.E-04  6.E-04  6.E-04  8.E-04  1.E-03  1.E-03  3.E-03 | _translation_  _glycolysis_  _regulation_of_transcription,_DNA-dependent_  _transcription_  _cell_cycle_  _oxidation_reduction_  _vacuolar_acidification_  _mitochondrial_translation_  _proton_transport_  _pyruvate_metabolic_process_ |
| F | 1.E-14  1.E-04  1.E-03  1.E-03  1.E-03  1.E-03  2.E-03  8.E-03  2.E-02  3.E-02 | _structural_constituent_of_ribosome_  _oxidoreductase_activity_  _hydrogen_ion_transmembrane_transporter_activity_  _DNA_binding_  _transcription_factor_activity_  _proton-transporting_ATPase_activity,_rotational_mechanism_  _P-P-bond-hydrolysis-driven_protein_transmembrane_transporter_activity_  _rRNA_binding_  _catalytic_activity_  _translation_initiation_factor_activity_ |
| C | 4.E-14  2.E-11  1.E-08  1.E-06  2.E-05  2.E-04  1.E-03  1.E-03  1.E-03  2.E-03 | _ribosome_  _ribonucleoprotein_complex_  _nucleus_  _mitochondrion_  _cytosolic_large_ribosomal_subunit_  _vacuolar_proton-transporting_V-type_ATPase,_V1_domain_  _endoplasmic_reticulum_  _small_ribosomal_subunit_  _intracellular_  _mitochondrial_outer_membrane_ |
| Cluster_3 1,059G [G2(58%), S, M] | | |
| P | 9.E-91  2.E-36  4.E-12  2.E-11  3.E-08  1.E-03  1.E-03  1.E-03  1.E-03  1.E-03 | _translation_  _translational_elongation_  _maturation_of_SSU-rRNA_from_tricistronic_rRNA_transcript_(SSU-rRNA,_5.8S_rRNA,_LSU-rRNA)_  _rRNA_export_from_nucleus_  _ribosomal_small_subunit_assembly_and_maintenance_  _ribosome_biogenesis_  _maintenance_of_fidelity_during_DNA-dependent_DNA_replication_  _glycine_betaine_transport_  _adenine_catabolic_process_  _nucleoside_diphosphate_phosphorylation_ |
| F | 2.E-117  2.E-04  1.E-03  1.E-03  1.E-03  1.E-03  1.E-03  1.E-03  1.E-03  1.E-03 | _structural_constituent_of_ribosome_  _RNA_binding_  _3-isopropylmalate_dehydrogenase_activity_  _flavin-linked_sulfhydryl_oxidase_activity_  _inositol_hexakisphosphate_kinase_activity_  _2-amino-4-hydroxy-6-hydroxymethyldihydropteridine_diphosphokinase_activity_  _nicotinate_phosphoribosyltransferase_activity_  _glycylpeptide_N-tetradecanoyltransferase_activity_  _RNA_lariat_debranching_enzyme_activity_  _oxidoreductase_activity,_acting_on_NADH_or_NADPH_ |
| C | 4.E-118  9.E-89  2.E-85  6.E-83  1.E-42  5.E-12  6.E-11  4.E-07  1.E-05  2.E-05 | cytosolic_small_ribosomal_subunit_  _ribosome_  _cytosolic_large_ribosomal_subunit_  _ribonucleoprotein_complex_  _intracellular_  _eukaryotic_translation_elongation_factor_1_complex_  _90S_preribosome_  _cytoplasm_  _small_ribosomal_subunit_  _small-subunit_processome_ |
| Cluster_4 73G [S, Transposon] | | |
| P | 3.E-12  1.E-11  7.E-07  9.E-07  2.E-05  6.E-05  2.E-04  6.E-04  6.E-04  1.E-03 | _transcription_  _regulation_of_transcription,_DNA-dependent_  _mitosis_  _cell_cycle_  _mRNA-binding_(hnRNP)_protein_import_into_nucleus_  _chromatin_modification_  _translation_  _ribosomal_protein_import_into_nucleus_  _snRNA_export_from_nucleus_  _ATP-dependent_chromatin_remodeling_ |
| F | 8.E-07  1.E-06  7.E-04  1.E-03  3.E-03  3.E-03  3.E-03  5.E-03  5.E-03  9.E-03 | _structural_constituent_of_ribosome_  _transcription_factor_activity_  _DNA_binding_  _zinc_ion_binding_  _protein_kinase_activity_  _protein_serine/threonine_kinase_activity_  _ATP-dependent_DNA_helicase_activity_  _catalytic_activity_  _transcription_activator_activity_  _protein_binding_ |
| C | 2.E-06  9.E-05  1.E-04  2.E-04  1.E-03  1.E-03  1.E-03  2.E-03  2.E-03  2.E-03 | _nucleus_  _ribosome_  _ribonucleoprotein_complex_  _SWI/SNF_complex_  _cellular_bud_  _nuclear_pore_  _spindle_pole_body_  _mitochondrion_  _trans-Golgi_network_  _cellular_bud_neck_contractile_ring_ |
| Cluster_5 1,311G [S,G2] | | |
| P | 3.E-32  1.E-22  2.E-06  2.E-06  6.E-06  1.E-03  1.E-03  1.E-03  1.E-03  1.E-03 | _ribosome_biogenesis_  _rRNA_processing_  _endonucleolytic_cleavage_to_generate_mature_5'-end_of_SSU-rRNA_from_(SSU-rRNA,_5.8S_rRNA,_LSU-rRNA)_  _endonucleolytic_cleavage_in_5'-ETS_of_tricistronic_rRNA_transcript_(SSU-rRNA,_5.8S_rRNA,_LSU-rRNA)_  _endonucleolytic_cleavage_in_ITS1_to_separate_SSU-rRNA_from_5.8S_rRNA_and_LSU-rRNA_from_tricistronic_rRNA_transcript_(SSU-rRNA,_5.8S_rRNA,_LSU-rRNA)_  _transport_  _oxidation_reduction_  _mitochondrial_translation_  _tRNA_processing_  _tRNA_modification_ |
| F | 5.E-05  1.E-03  2.E-03  7.E-03  1.E-02  2.E-02  2.E-02  3.E-02  3.E-02  3.E-02 | _snoRNA_binding_  _oxidoreductase_activity_  _ATP-dependent_RNA_helicase_activity_  _RNA-dependent_ATPase_activity_  _structural_constituent_of_ribosome_  _ribonuclease_MRP_activity_  _ribonuclease_P_activity_  _tRNA_methyltransferase_activity_  _RNA_binding_  _RNA_methyltransferase_activity_ |
| C | 6.E-22  3.E-14  1.E-11  1.E-09  5.E-07  3.E-04  1.E-03  1.E-03  1.E-03  1.E-03 | _nucleolus_  _mitochondrion_  _nucleus_  _90S_preribosome_  _membrane_  _mitochondrial_inner_membrane_  _small-subunit_processome_  _integral_to_membrane_  _Pwp2p-containing_subcomplex_of_90S_preribosome_  _mitochondrial_outer_membrane_ |
