# Supplementary material for: A Geometric Clustering Tool (AGCT) to robustly unravel the inner cluster structures of time-series gene expressions
Source: PLoS One. 2020 Jul 6;15(7):e0233755. doi: 10.1371/journal.pone.0233755 (PMC7337352; doi:10.1371/journal.pone.0233755)
Supplement: S1 Fig — (PDF) [file pone.0233755.s010.pdf]

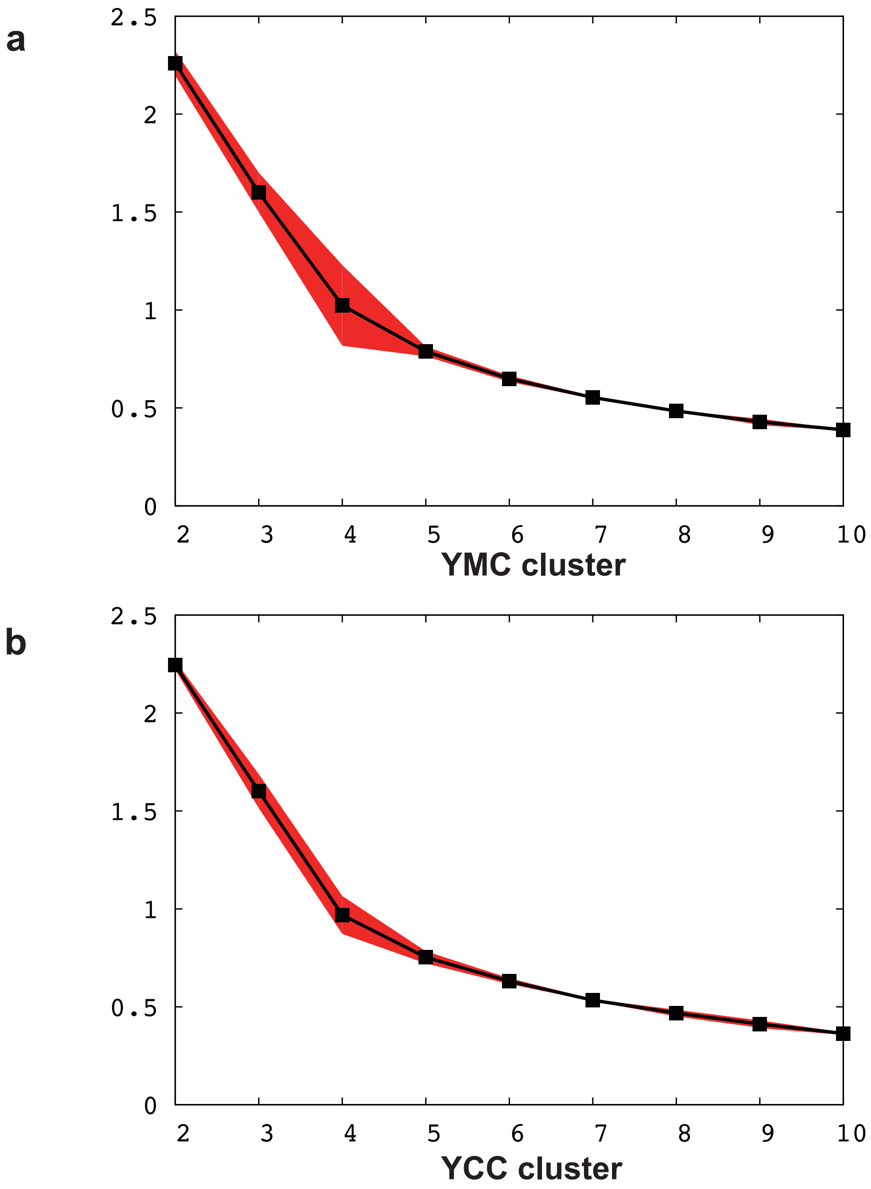

Figure S15: Black line is an average k-means potential (with Arthur-Vassilvitskii initialization); red area is ±σ area around mean.
