# Supplementary material for: A Geometric Clustering Tool (AGCT) to robustly unravel the inner cluster structures of time-series gene expressions
Source: PLoS One. 2020 Jul 6;15(7):e0233755. doi: 10.1371/journal.pone.0233755 (PMC7337352; doi:10.1371/journal.pone.0233755)
Supplement: S2 Fig — (a) Two clusters obtained with Non-Negative Matrix Factorization method on the Yeast Metabolic Cycle dataset (9,334G): Cluster 0 (596G) and Cluster 1(8,738G).(b) YMC and YCC sentinel genes belong to Cluster 1, space where most colored circles are observed. Delaunay triangulations are shown with green edges. (c, d) Cluster expression profile, with candlesticks (solid curves: in red, average values; in black, median values). Boxes indicate Q25, Q50 (black), and Q75 quantiles. (e, f) Dimension histograms for each cluster. (PDF) [file pone.0233755.s011.pdf]

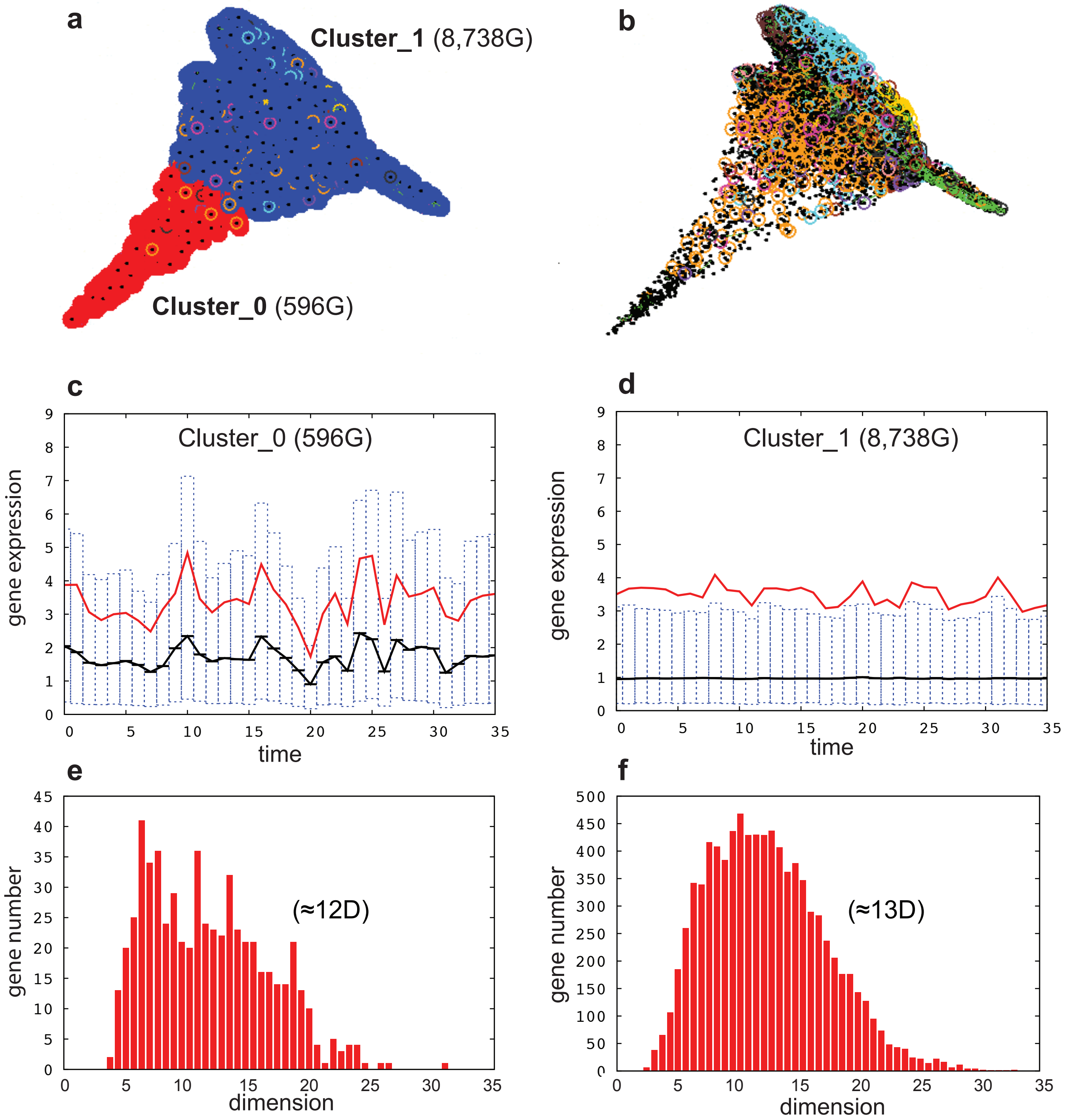

Figure S16: (a) Two clusters obtained with Non-Negative Matrix Factorization method on the Yeast Metabolic Cycle dataset (9,334G): Cluster 0 (596G) and Cluster 1(8,738G).
(b) YMC and YCC sentinel genes belong to Cluster 1, space where most colored circles are observed. Delaunay triangulations are shown with green edges. (c, d) Cluster expression profile, with candlesticks (solid curves: in red, average values; in black, median values).
Boxes indicate Q25, Q50 (black), and Q75 quantiles. (e, f) Dimension histograms for each cluster.
