# Supplementary material for: A Geometric Clustering Tool (AGCT) to robustly unravel the inner cluster structures of time-series gene expressions
Source: PLoS One. 2020 Jul 6;15(7):e0233755. doi: 10.1371/journal.pone.0233755 (PMC7337352; doi:10.1371/journal.pone.0233755)
Supplement: S3 Fig — Reductive/Charging and Oxidative sentinels are detected in groups up (blue) and down (light green) on the manifold edges (a,b).(a) M-G1 phase sentinels bridging two clusters to promote cell cycle progression. (b) High local dimension genes of YMC are in the core of Reductive/Charging cluster 2 and around “M-G1—G1” bridge area (a), specifying Barycenter of YMC dataset. (PDF) [file pone.0233755.s012.pdf]

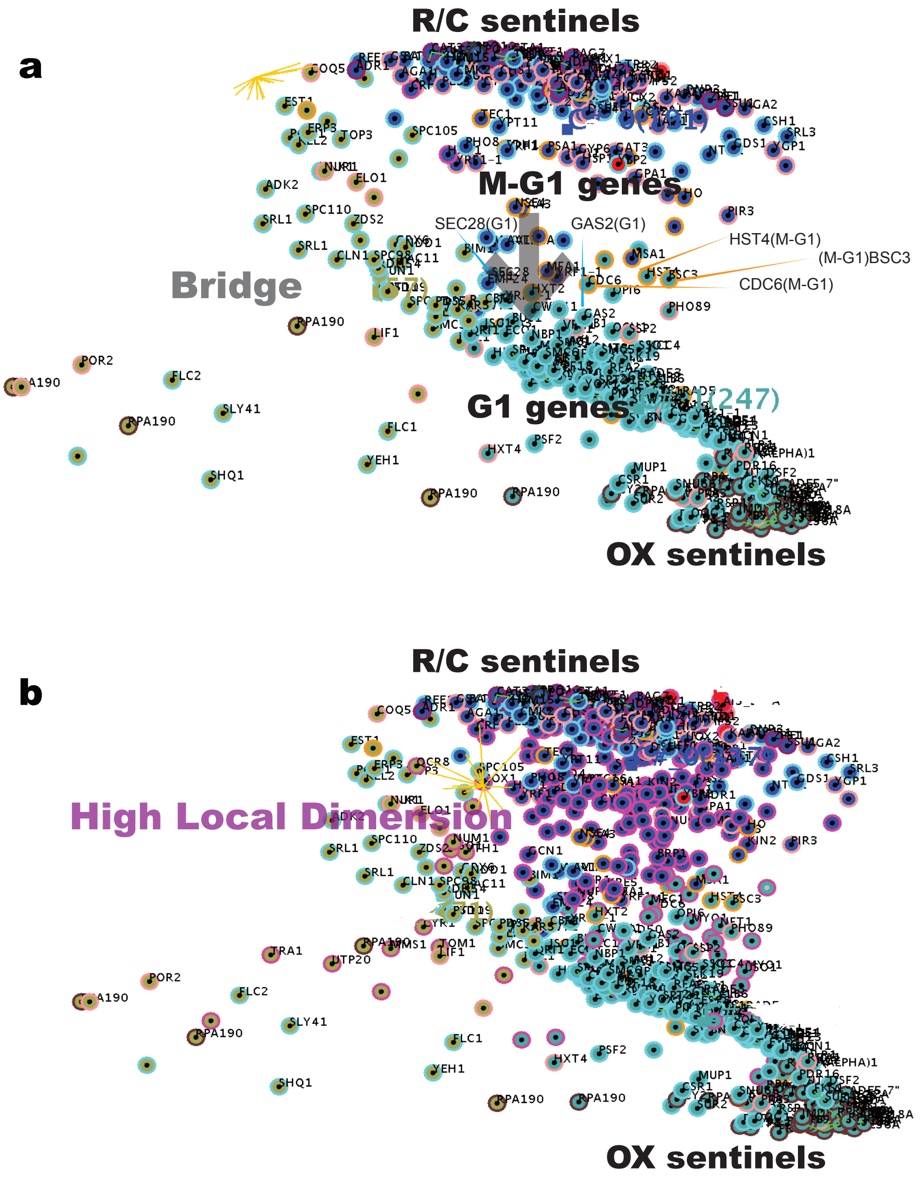

Figure S17: The transition between M-G1 phase (Reductive/Charging cluster 2) and G1 phase (Oxidative cluster 1) of Yeast Metabolic Cycle. Reductive/Charging and Oxidative sentinels are detected in groups up (blue) and down (light green) on the manifold edges (a,b).
(a) M-G1 phase sentinels bridging two clusters to promote cell cycle progression.
(b) High local dimension genes of YMC are in the core of Reductive/Charging cluster 2 and around "M-G1 - G1" bridge area (a), specifying Barycenter of YMC dataset.
