# Supplementary material for: A Geometric Clustering Tool (AGCT) to robustly unravel the inner cluster structures of time-series gene expressions
Source: PLoS One. 2020 Jul 6;15(7):e0233755. doi: 10.1371/journal.pone.0233755 (PMC7337352; doi:10.1371/journal.pone.0233755)
Supplement: S4 Fig — Blue—Metabolic genes (ADH2, ACS1, ATO2, FDH1, POX1, FOX2), Red—YCC genes (EGT23, DSE3, TIP1, TSL1, Spellman at al.), Green—Yeast Cell Cycle (NCE102 PFK26, PRR2) identified in this study. The thick red line corresponds to the enlarged region. (PDF) [file pone.0233755.s013.pdf]

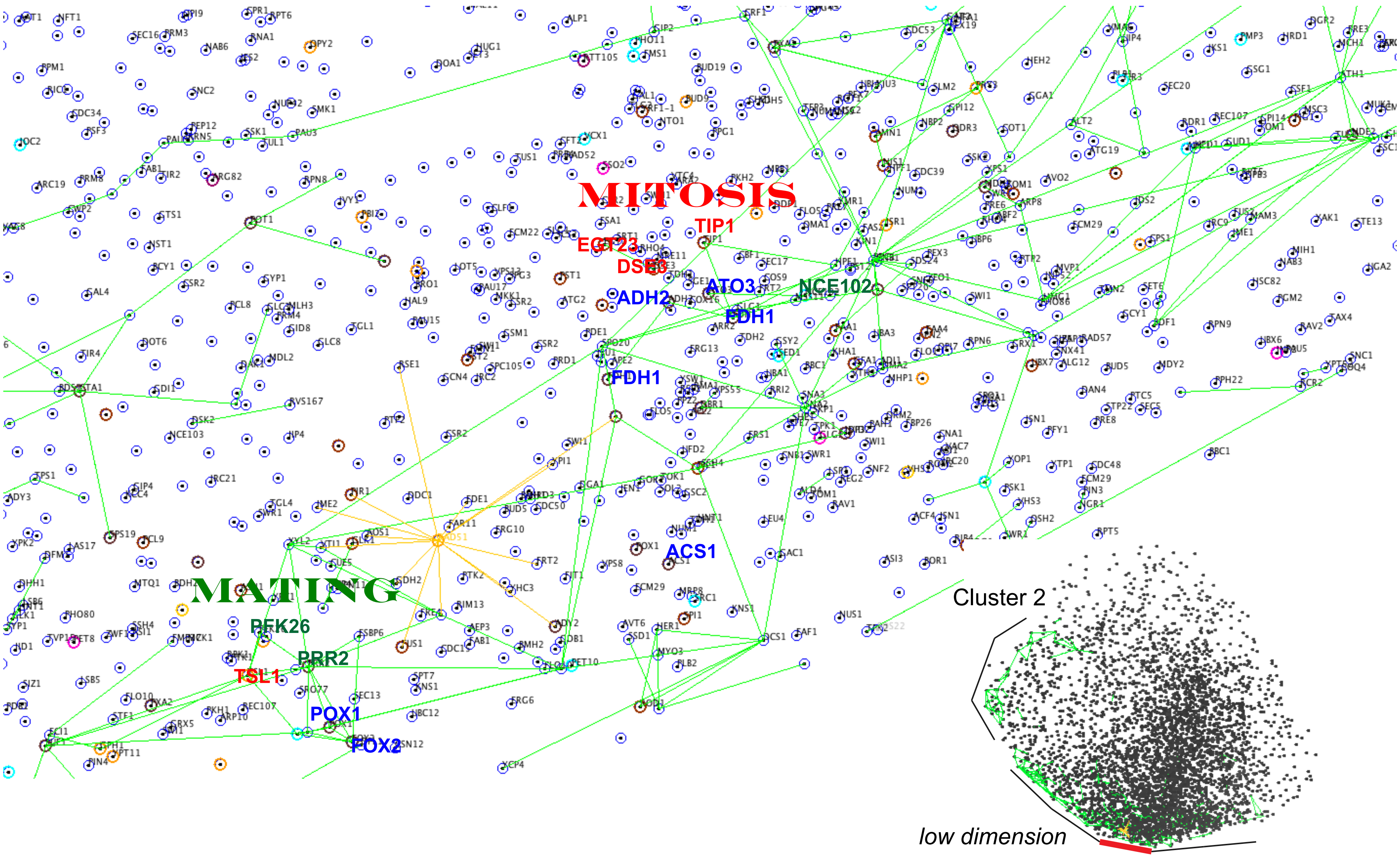

Figure S18: A network of highly co-regulated periodic genes (Spellman et al.) extracted on top of Delaunay triangulation (p≤0.001) of Reductive/Charging Cluster 2 (4,572G). Blue - Metabolic genes (ADH2, ACS1, ATO2, FDH1, POX1, FOX2), Red - YCC genes (EGT23, DSE3, TIP1, TSL1, Spellman at al.), Green - Yeast Cell Cycle (NCE102 PFK26, PRR2) identified in this study. The thick red line corresponds to the enlarged region.
