# Supplementary material for: A Geometric Clustering Tool (AGCT) to robustly unravel the inner cluster structures of time-series gene expressions
Source: PLoS One. 2020 Jul 6;15(7):e0233755. doi: 10.1371/journal.pone.0233755 (PMC7337352; doi:10.1371/journal.pone.0233755)
Supplement: S7 Fig — Green and red edges equivalent to positive and negative correlations according to Delaunay triangulation (p≤0.001). (PDF) [file pone.0233755.s016.pdf]

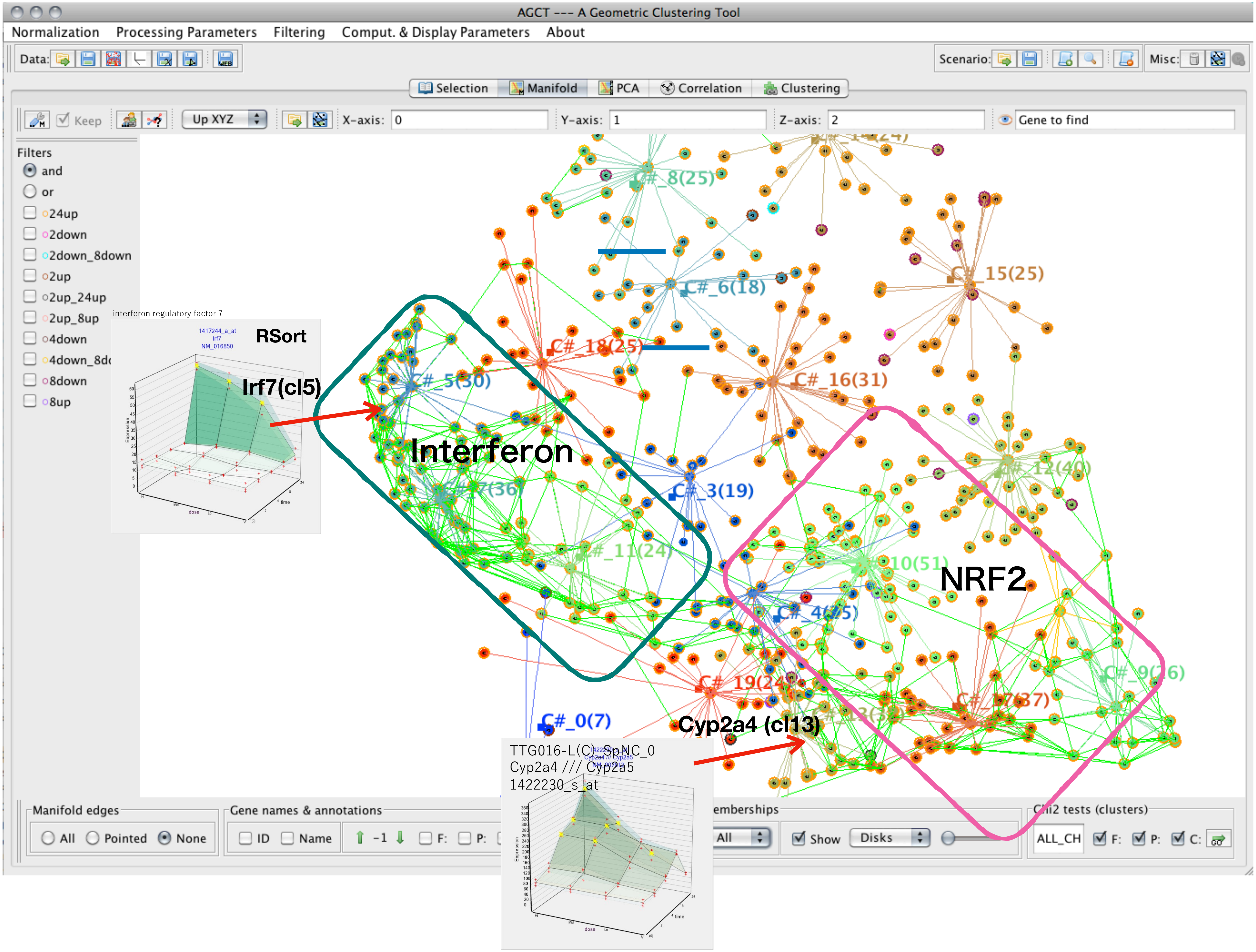

AGCT --- A Geometric Clustering Tool
Normalization
Processing Parameters
Filtering
Comput. & Display Parameters
About
Data:
Scenario:
Misc:
Selection
Manifold
PCA
Correlation
Clustering
Keep
Up XYZ
X-axis: 0
Y-axis: 1
Z-axis: 2
Gene to find
Filters
and
or
o24up
o2down
o2down_8down
o2up
o2up_24up
o2up_8up
o4down
o4down_8d
o8down
o8up
interferon regulatory factor 7
1417244_a_at
Irf7
NM_016850
RSort
Irf7(cl5)
Interferon
C#_8(25)
C#_6(18)
C#_15(25)
C#_18(25)
C#_5(30)
C#_16(31)
C#_12(40)
C#_3(19)
C#_7(36)
C#_11(24)
C#_10(51)
NRF2
C#_4(25)
C#_9(26)
C#_19(24)
C#_17(37)
C#_0(7)
Cyp2a4 (cl13)
TTG016-L(C)SpNC_0
Cyp2a4 /// Cyp2a5
1422230_s_at
Manifold edges
All
Pointed
None
Gene names & annotations
ID
Name
-1
F:
P:
emberships
All
Show
Disks
Chi2 tests (clusters)
ALL_CH
F:
P:
C:
