# Supplementary material for: A Geometric Clustering Tool (AGCT) to robustly unravel the inner cluster structures of time-series gene expressions
Source: PLoS One. 2020 Jul 6;15(7):e0233755. doi: 10.1371/journal.pone.0233755 (PMC7337352; doi:10.1371/journal.pone.0233755)

Expander A Gene Expression Analysis and Visualization Software

**A.**

(1) Two datasets of 9335 and 3656 probes were run on EXPANDER using their CLICK algorithm. EXPANDER has provided the clustering info which can be seen in 2 and 3, an expression matrix in 4 and mean cluster pattern in 5.

(2) Clustering information for 9335 genes provided by EXPANDER.

**Clustering Info:**
Algorithm: CLICK
Expected mean homogeneity: Not specified (default)
Overall Average Homogeneity: 0.943

| ID | Name | Size | Homogeneity |
|---|---|---|---|
| 1 | Cluster_1 | 9277 | 0.943 |

(3) Clustering information for 3565 genes provided by EXPANDER.

**Clustering Info:**
Algorithm: CLICK
Expected mean homogeneity: Not specified (default)
Overall Average Homogeneity: 0.765
Overall Average Separation: -0.221
Number of clusters: 11
Number of singletons: 0

| ID | Name | Size | Homogeneity |
|---|---|---|---|
| 1 | Cluster_1 | 1194 | 0.787 |
| 2 | Cluster_2 | 900 | 0.809 |
| 3 | Cluster_3 | 707 | 0.789 |
| 4 | Cluster_4 | 281 | 0.758 |
| 5 | Cluster_5 | 174 | 0.728 |
| 6 | Cluster_6 | 125 | 0.538 |
| 7 | Cluster_7 | 115 | 0.612 |
| 8 | Cluster_8 | 71 | 0.600 |
| 9 | Cluster_9 | 39 | 0.558 |
| 10 | Cluster_10 | 32 | 0.592 |
| 11 | Cluster_11 | 18 | 0.531 |

(4) Expression matrix provided by EXPANDER.

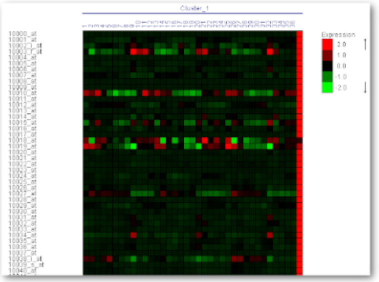

(5) Mean cluster patter for cluster 1 on 9335 probes, provided by EXPANDER.

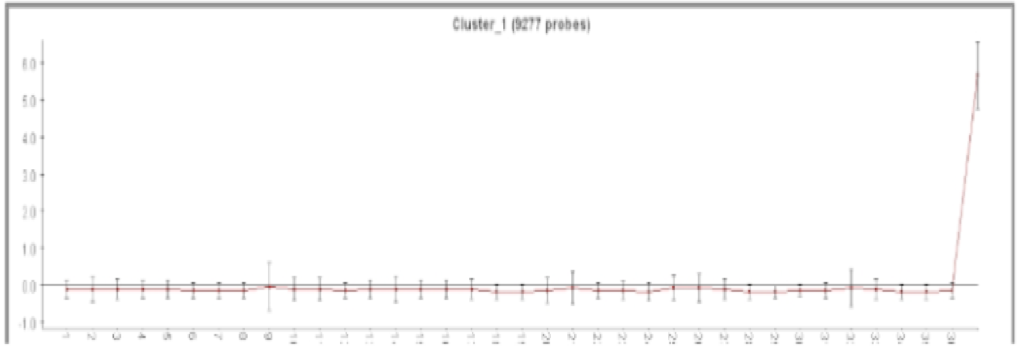


(6) Results from clustering 9335 and 3656 probes using EXPANDER's CLICK algorithm. Classification of Oxidative, Reactive Building and Reactive Charging genes.

| Clusters (1) | Probes (9335) | OXD (61) | RB (40) | RC (45) | NOC |
|---|---|---|---|---|---|
| 0 | 58 | 0 | 0 | 1 | - |
| Cluster_1 | 9277 | **61** | **40** | **44** | 84 |
| Total | 9277 | 61 | 40 | 44 | 145 |
| Missing | 0.62% | 0.00% | 0.00% | 2.22% | 0.68% |
| Probe Cluster Ability | | | | | 99.38% |
| Cluster Collisions | | | | | 57.93% |
| Sentinal Cluster Efficiency | | 100.00% | 100.00% | 100.00% | 100.00% |
| Sentinal Clustering Ability | | | | | 99.32% |

| Clusters (11) | Probes (3656) | OXD (55) | RB (40) | RC (41) | NOC |
|---|---|---|---|---|---|
| Cluster_1 | 1194 | 0 | 0 | **40** | 0 |
| Cluster_2 | 900 | **55** | 0 | 0 | 0 |
| Cluster_3 | 707 | 0 | **40** | 0 | 0 |
| Cluster_4 | 281 | 0 | 0 | **1** | 0 |
| Cluster_5 | 174 | 0 | 0 | 0 | 0 |
| Cluster_6 | 125 | 0 | 0 | 0 | 0 |
| Cluster_7 | 115 | 0 | 0 | 0 | 0 |
| Cluster_8 | 71 | 0 | 0 | 0 | 0 |
| Cluster_9 | 39 | 0 | 0 | 0 | 0 |
| Cluster_10 | 32 | 0 | 0 | 0 | 0 |
| Cluster_11 | 18 | 0 | 0 | 0 | 0 |
| Total | 3656 | 55 | 40 | 41 | 136 |
| Mission | 0.00% | 0.00% | 0.00% | 0.00% | 0 |
| Probe Cluster Ability | | | | | 100.00% |
| Cluster Collisions | | | | | 0.00% |
| Sentinal Cluster Efficiency | | 100.00% | 100.00% | 97.56% | 99.19% |
| Sentinal Clustering Ability | | | | | 100.00% |

EXpander A Gene Expression Analysis and Visualization Software

**B.**

(1) Two datasets of 9335 and 3565 genes were run on the EXPANDER using their Biclustering algorithm SAMBA. EXPANDER has provided clustering information in 2, clusters for the genes in 3, and expression matrix for each cluster in 4.

(5) Results from clustering 9335 and 3656 probes using EXPANDER's SAMBA algorithm. Classification of Oxidative, Reactive Building and Reactive Charging genes.

| Clusters (25) | Probes (9335) | OXD (61) | RB (40) | RC (45) | NOC |
|---|---|---|---|---|---|
| 1 | 553 | **16** | 0 | **21** | 16 |
| 2 | 551 | 0 | 0 | **3** | 0 |
| 3 | 541 | **7** | 0 | 0 | 0 |
| 4 | 478 | **18** | 0 | 0 | 0 |
| 5 | 544 | 0 | **3** | 0 | 0 |
| 6 | 487 | 0 | 0 | 0 | 0 |
| 7 | 539 | 0 | 0 | 0 | 0 |
| 8 | 546 | 0 | 0 | 0 | 0 |
| 9 | 529 | 0 | 0 | 0 | 0 |
| 10 | 452 | 0 | 0 | 0 | 0 |
| 11 | 535 | 0 | 0 | 0 | 0 |
| 12 | 182 | 0 | 0 | 0 | 0 |
| 13 | 185 | 0 | 0 | 0 | 0 |
| 14 | 189 | 0 | 0 | 0 | 0 |
| 15 | 202 | 0 | 0 | 0 | 0 |
| 16 | 170 | 0 | 0 | 0 | 0 |
| 17 | 203 | 0 | 0 | 0 | 0 |
| 18 | 196 | 0 | 0 | **2** | 0 |
| 19 | 181 | 0 | 0 | 0 | 0 |
| 20 | 152 | 0 | 0 | 0 | 0 |
| 21 | 191 | 0 | 0 | 0 | 0 |
| 22 | 210 | 0 | 0 | 0 | 0 |
| 23 | 202 | 0 | 0 | 0 | 0 |
| 24 | 195 | 0 | 0 | 0 | 0 |
| 25 | 192 | 0 | 0 | 0 | 0 |
| Total | 8405 | 41 | 3 | 26 | 70 |
| Missing | 10% | 33% | 93% | 42% | 52.05% |
| Probe Cluster Ability | | | | | 90% |
| Cluster Collisions | | | | | 23% |
| Sentinal Cluster Efficiency | | 44% | 100% | 81% | 75% |
| Sentinal Clustering Ability | | | | | 47.95% |

| Clusters (21) | Probes (3656) | OXD (55) | RB (40) | RC (41) | NOC |
|---|---|---|---|---|---|
| 1 | 232 | **2** | 0 | **18** | 2.00 |
| 2 | 199 | 0 | 0 | 0 | 0.00 |
| 3 | 193 | **11** | 0 | 0 | 0.00 |
| 4 | 223 | 0 | 0 | **1** | 0.00 |
| 5 | 176 | **13** | 0 | 0 | 0.00 |
| 6 | 200 | 0 | 0 | 0 | 0.00 |
| 7 | 212 | 0 | 0 | 0 | 0.00 |
| 8 | 194 | **7** | 0 | 0 | 0.00 |
| 9 | 223 | 0 | 0 | 0 | 0.00 |
| 10 | 143 | **0** | 0 | 0 | 0.00 |
| 11 | 130 | 0 | 0 | 0 | 0.00 |
| 12 | 138 | 0 | 0 | 0 | 0.00 |
| 13 | 149 | 0 | 0 | **3** | 0.00 |
| 14 | 153 | 0 | 0 | 0 | 0.00 |
| 15 | 87 | 0 | 0 | 0 | 0.00 |
| 16 | 113 | 0 | 0 | 0 | 0.00 |
| 17 | 143 | 0 | 0 | 0 | 0.00 |
| 18 | 118 | 0 | 0 | 0 | 0.00 |
| 19 | 135 | 0 | 0 | 0 | 0.00 |
| 20 | 125 | 0 | 0 | 0 | 0.00 |
| 21 | 161 | 0 | 0 | 0 | 0.00 |
| Total | 3447 | 33 | 0 | 22 | 55 |
| Missing | 5.72% | 40.00% | 100.00% | 46.34% | 81 |
| Probe Cluster Ability | | | | | 94.28% |
| Cluster Collisions | | | | | 3.64% |
| Sentinal Cluster Efficiency | | 39.39% | - | 81.82% | 60.61% |
| Sentinal Clustering Ability | | | | | 40.44% |

(4) Expression matrix for Bicluster 15 provided by EXPANDER.

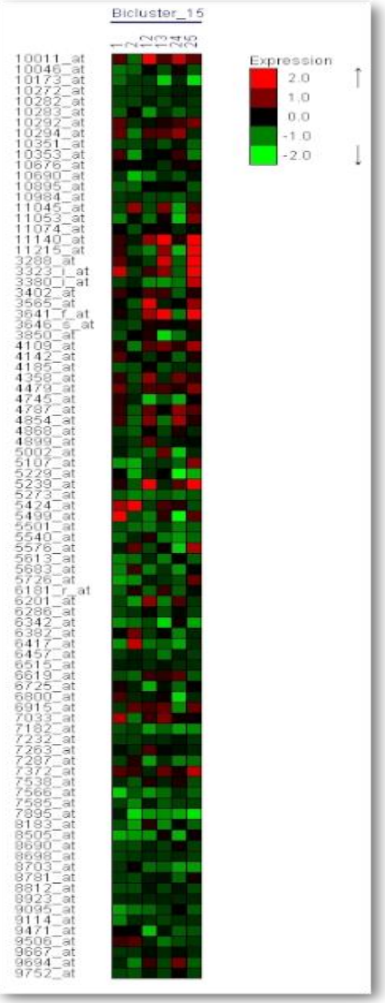


(2) Cluster information provided by EXPANDER.

**Biclustering info:**

Matrix data: GE Data 2 1
option files location input: valsp_3ap
Permitted overlap between two biclusters: 0.1
try covering all probes input: false
Take log input: 1
Maximal memory allocated for hashing stage: 100
Maximal kernel size in the hashing stage: 4
Minimal kernel size in the hashing stage: 4
Minimal number of responding probes per condition: 100
Total number of biclusters: 21

(3) Clustering results from SAMBA provided by EXPANDER.

| ID | Name | Score | #Conditions | #Probes |
|---|---|---|---|---|
| 1 | Bicluster_1 | 1282.85 | 18 | 232 |
| 2 | Bicluster_2 | 890.237 | 14 | 199 |
| 3 | Bicluster_3 | 995.515 | 18 | 193 |
| 4 | Bicluster_4 | 1004.94 | 15 | 223 |
| 5 | Bicluster_5 | 1710.33 | 32 | 176 |
| 6 | Bicluster_6 | 1221.89 | 20 | 200 |
| 7 | Bicluster_7 | 1381.2 | 24 | 212 |
| 8 | Bicluster_8 | 836.576 | 14 | 194 |
| 9 | Bicluster_9 | 1123.9 | 18 | 223 |
| 10 | Bicluster_10 | 623.066 | 17 | 143 |
| 11 | Bicluster_11 | 901.286 | 26 | 130 |
| 12 | Bicluster_12 | 925.303 | 25 | 138 |
| 13 | Bicluster_13 | 593.831 | 14 | 149 |
| 14 | Bicluster_14 | 651.373 | 15 | 153 |
| 15 | Bicluster_15 | 140.79 | 6 | 87 |
| 16 | Bicluster_16 | 620.859 | 21 | 113 |
| 17 | Bicluster_17 | 629.875 | 15 | 143 |
| 18 | Bicluster_18 | 906.688 | 28 | 118 |
| 19 | Bicluster_19 | 770.904 | 19 | 135 |
| 20 | Bicluster_20 | 908.645 | 28 | 125 |
| 21 | Bicluster_21 | 680.989 | 15 | 161 |

C.

# Expander A Gene Expression Analysis and Visualization Software

(1) Two datasets of time series gene expression data (of sizes 9335 and 3656) were inputted into EXPANDER and run on the using SOM algorithm. EXPANDER provided cluster information in which can be seen in 2, an Expression Matrix in 3 and mean cluster patterns in 4.

(2) Cluster information provided by EXPANDER.

**Clustering Info:**
Algorithm: SOM
SOM K: 30
SOM L: 24
Iterations: 20000
Overall Average Homogeneity: 0.499
Overall Average Separation: 0.098
Number of clusters: 36
Number of singletons: 0

| ID | Name | Size | Homogeneity |
|---|---|---|---|
| 1 | Cluster_1 | 190 | 0.613 |
| 2 | Cluster_2 | 69 | 0.339 |
| 3 | Cluster_3 | 226 | 0.662 |
| 4 | Cluster_4 | 1045 | 0.132 |
| 5 | Cluster_5 | 198 | 0.526 |
| 6 | Cluster_6 | 28 | 0.372 |
| 7 | Cluster_7 | 53 | 0.881 |
| 8 | Cluster_8 | 75 | 0.410 |
| 9 | Cluster_9 | 212 | 0.701 |
| 10 | Cluster_10 | 174 | 0.764 |
| 11 | Cluster_11 | 134 | 0.540 |
| 12 | Cluster_12 | 110 | 0.782 |
| 13 | Cluster_13 | 159 | 0.727 |
| 14 | Cluster_14 | 130 | 0.614 |
| 15 | Cluster_15 | 92 | 0.651 |
| 16 | Cluster_16 | 51 | 0.545 |
| 17 | Cluster_17 | 99 | 0.702 |
| 18 | Cluster_18 | 52 | 0.724 |
| 19 | Cluster_19 | 13 | 0.523 |
| 20 | Cluster_20 | 174 | 0.623 |
| 21 | Cluster_21 | 62 | 0.821 |
| 22 | Cluster_22 | 92 | 0.816 |
| 23 | Cluster_23 | 56 | 0.367 |
| 24 | Cluster_24 | 40 | 0.751 |
| 25 | Cluster_25 | 1 | NaN |
| 26 | Cluster_26 | 8 | 0.707 |
| 27 | Cluster_27 | 10 | 0.818 |
| 28 | Cluster_28 | 50 | 0.734 |
| 29 | Cluster_29 | 4 | 0.166 |
| 30 | Cluster_30 | 4 | 0.527 |
| 31 | Cluster_31 | 16 | 0.607 |
| 32 | Cluster_32 | 2 | 0.501 |
| 33 | Cluster_33 | 6 | 0.876 |
| 34 | Cluster_34 | 11 | 0.918 |
| 35 | Cluster_35 | 6 | 0.211 |
| 36 | Cluster_36 | 4 | -0.029 |

(3) Expression Matrix for cluster 24 provided by EXPANDER.

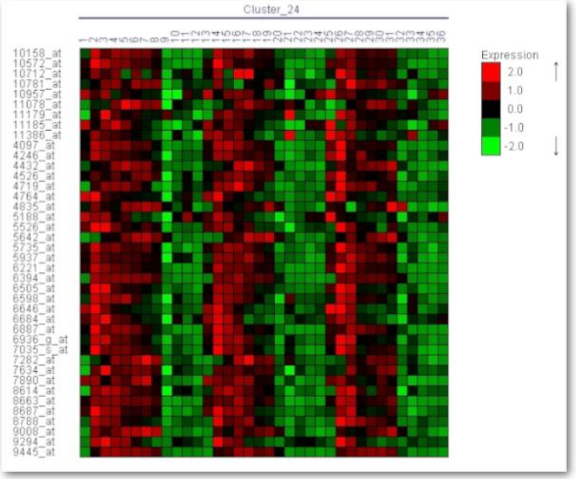


(4) Mean pattern for cluster 24 provided by EXPANDER on the 3656 dataset.

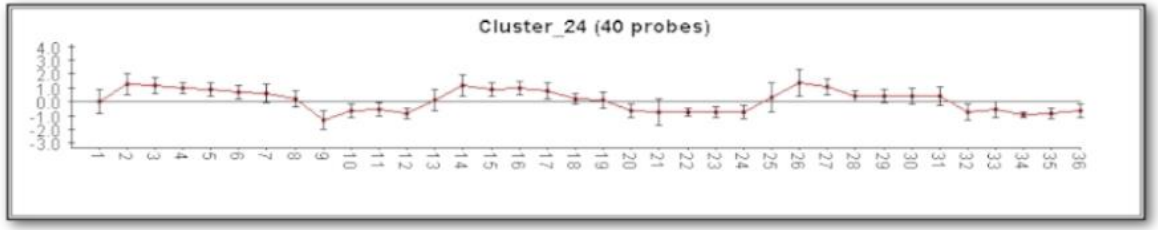


(5) Clustering results from EXPANDER SOM algorithm on 9335 and 3656 probes. Classification of Oxidative, Reactive Building and Reactive Charging genes into clusters.

| Clusters (46) | Probes (9335) | OXD (61) | RB (40) | RC (45) | NOC |
|---|---|---|---|---|---|
| 1 | 3112 | 4 | 0 | 2 | 2.00 |
| 2 | 284 | 0 | 0 | 0 | 0.00 |
| 3 | 37 | 0 | 0 | 0 | 0.00 |
| 4 | 73 | 16 | 0 | 0 | 0.00 |
| 5 | 267 | 5 | 3 | 0 | 3.00 |
| 6 | 224 | 1 | 0 | 0 | 0.00 |
| 7 | 255 | 0 | 0 | 3 | 0.00 |
| 8 | 80 | 0 | 0 | 0 | 0.00 |
| 9 | 219 | 0 | 0 | 1 | 0.00 |
| 10 | 141 | 0 | 2 | 1 | 1.00 |
| 11 | 74 | 1 | 2 | 0 | 1.00 |
| 12 | 231 | 1 | 11 | 0 | 1.00 |
| 13 | 103 | 4 | 0 | 2 | 2.00 |
| 14 | 151 | 0 | 0 | 0 | 0.00 |
| 15 | 369 | 0 | 1 | 1 | 1.00 |
| 16 | 231 | 0 | 0 | 0 | 0.00 |
| 17 | 188 | 0 | 0 | 0 | 0.00 |
| 18 | 214 | 0 | 0 | 11 | 0.00 |
| 19 | 330 | 0 | 2 | 0 | 0.00 |
| 20 | 164 | 0 | 0 | 4 | 0.00 |
| 21 | 122 | 2 | 0 | 7 | 2.00 |
| 22 | 50 | 0 | 0 | 3 | 0.00 |
| 23 | 277 | 0 | 2 | 0 | 0.00 |
| 24 | 234 | 0 | 0 | 1 | 0.00 |
| 25 | 98 | 10 | 0 | 0 | 0.00 |
| 26 | 133 | 0 | 4 | 0 | 0.00 |
| 27 | 100 | 1 | 0 | 0 | 0.00 |
| 28 | 224 | 1 | 0 | 0 | 0.00 |
| 29 | 189 | 0 | 0 | 0 | 0.00 |
| 30 | 253 | 0 | 3 | 0 | 0.00 |
| 31 | 77 | 0 | 0 | 1 | 0.00 |
| 32 | 52 | 0 | 2 | 0 | 0.00 |
| 33 | 182 | 0 | 3 | 0 | 0.00 |
| 34 | 37 | 0 | 0 | 0 | 0.00 |
| 35 | 147 | 0 | 0 | 5 | 0.00 |
| 36 | 131 | 6 | 3 | 0 | 3.00 |
| 37 | 37 | 0 | 0 | 0 | 0.00 |
| 38 | 93 | 0 | 0 | 0 | 0.00 |
| 39 | 5 | 0 | 0 | 0 | 0.00 |
| 40 | 13 | 0 | 2 | 0 | 0.00 |
| 41 | 49 | 0 | 0 | 2 | 0.00 |
| 42 | 41 | 5 | 0 | 0 | 0.00 |
| 43 | 11 | 0 | 0 | 0 | 0.00 |
| 44 | 12 | 0 | 0 | 0 | 0.00 |
| 45 | 11 | 0 | 0 | 1 | 0.00 |
| 46 | 10 | 4 | 0 | 0 | 0.00 |
| Total | 9335 | 61 | 40 | 45 | 146.00 |
| Missing | 0% | 0% | 0% | 0% | 0.00 |
| Probe Cluster Ability | | | | | 100% |
| Cluster Collisions | | | | | 11% |
| Sentinal Cluster Efficiency | | 26% | 28% | 24% | 26% |
| Sentinal Clustering Ability | | | | | 100.00% |

| Cluster (37) | Probes (3656) | OXD (55) | RB (40) | RC (41) | NOC |
|---|---|---|---|---|---|
| 1 | 190 | 0 | 7 | 0 | 0.00 |
| 2 | 69 | 7 | 0 | 0 | 0.00 |
| 3 | 226 | 0 | 2 | 0 | 0.00 |
| 4 | 1045 | 1 | 4 | 3 | 4.00 |
| 5 | 198 | 0 | 0 | 0 | 0.00 |
| 6 | 28 | 0 | 0 | 0 | 0.00 |
| 7 | 53 | 11 | 0 | 0 | 0.00 |
| 8 | 75 | 3 | 0 | 4 | 3.00 |
| 9 | 212 | 0 | 0 | 3 | 0.00 |
| 10 | 174 | 0 | 0 | 6 | 0.00 |
| 11 | 134 | 0 | 1 | 0 | 0.00 |
| 12 | 110 | 0 | 0 | 8 | 0.00 |
| 13 | 159 | 0 | 0 | 3 | 0.00 |
| 14 | 130 | 3 | 4 | 0 | 3.00 |
| 15 | 92 | 10 | 1 | 0 | 1.00 |
| 16 | 51 | 8 | 0 | 0 | 0.00 |
| 17 | 99 | 0 | 11 | 0 | 0.00 |
| 18 | 52 | 0 | 4 | 0 | 0.00 |
| 19 | 13 | 0 | 0 | 0 | 0.00 |
| 20 | 174 | 5 | 6 | 0 | 5.00 |
| 21 | 62 | 0 | 0 | 6 | 0.00 |
| 22 | 92 | 5 | 0 | 0 | 0.00 |
| 23 | 56 | 0 | 0 | 0 | 0.00 |
| 24 | 40 | 0 | 0 | 4 | 0.00 |
| 25 | 1 | 0 | 0 | 0 | 0.00 |
| 26 | 8 | 0 | 0 | 0 | 0.00 |
| 27 | 10 | 0 | 0 | 0 | 0.00 |
| 28 | 50 | 0 | 0 | 1 | 0.00 |
| 29 | 4 | 0 | 0 | 0 | 0.00 |
| 30 | 4 | 0 | 0 | 0 | 0.00 |
| 31 | 16 | 2 | 0 | 0 | 0.00 |
| 32 | 2 | 0 | 0 | 0 | 0.00 |
| 33 | 6 | 0 | 0 | 1 | 0.00 |
| 34 | 11 | 0 | 0 | 2 | 0.00 |
| 35 | 6 | 0 | 0 | 0 | 0.00 |
| 36 | 4 | 0 | 0 | 0 | 0.00 |
| Total | 3656 | 55 | 40 | 41 | 136 |
| Mission | 0.00% | 0.00% | 0.00% | 0.00% | 0 |
| Probe Cluster Ability | | | | | 100.00% |
| Cluster Collisions | | | | | 11.76% |
| Sentinal Cluster Efficiency | | 20.00% | - | 19.51% | 19.76% |
| Sentinal Clustering Ability | | | | | 100.00% |

# Short Time-series Expression Miner (STEM)

**D.**

(1) Two datasets of sizes 9335 and 3656 of time-series gene expression data were inputted into the Short Time-series Expression Miner (STEM). The process and results can be seen in 2, 3, 4 and 5.

(5) The results of the clustering on the datasets can be seen below. The datasets were run twice once with the ‘m’ parameter set to 3 and 7, and again set to the default 50. The table shows number of Oxidative, Reactive and Reactive Charging genes in each cluster.

(2) STEM import window.

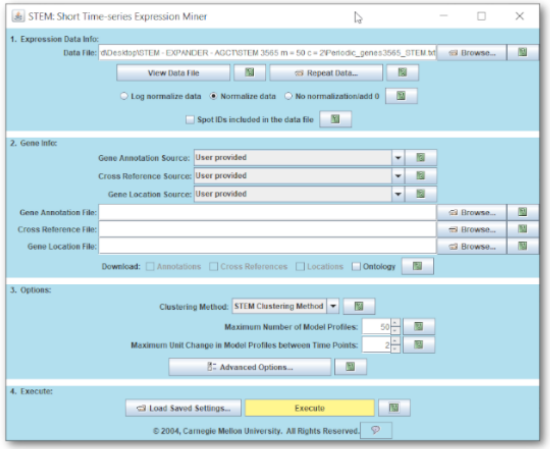


(3) STEM profile window.

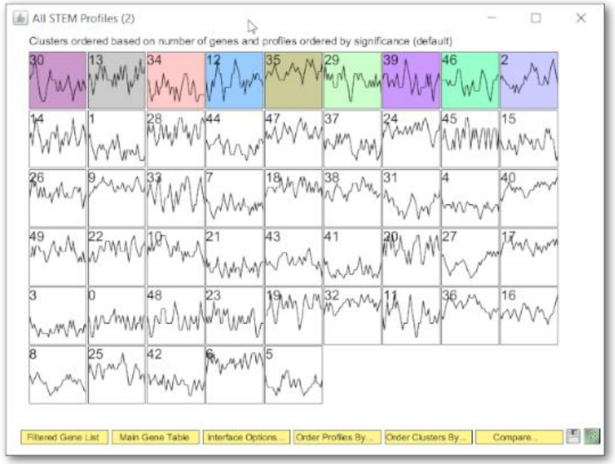


(4) STEM gene expression profile, for profile 30.

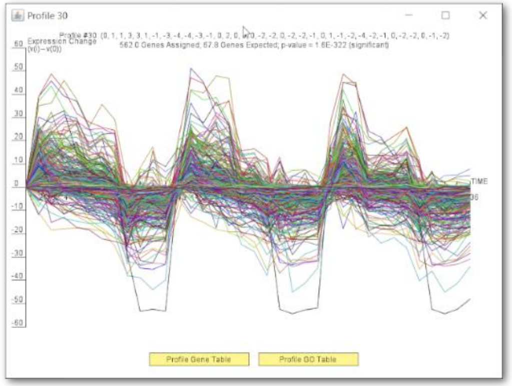


STEM v1

| Profiles (3) | Probes (9335) | OXD (61) | RB (40) | RC (45) | NOC |
|---|---|---|---|---|---|
| 1 | 1698 | 0 | 0 | **24** | 0 |
| 2 | 1245 | 0 | 0 | 0 | 0 |
| 3 | 167 | 0 | 0 | 0 | 0 |
| Total | 3110 | 0 | 0 | 24 | 24 |
| Missing | 66.68% | 100.00% | 100.00% | 46.67% | 122 |
| Probe Cluster Ability | | | | | 33.32% |
| Cluster Collisions | | | | | 0.00% |
| Sentinal Cluster Efficiency | | - | - | 100.00% | 100.00% |
| Sentinal Clustering Ability | | | | | 16.44% |

| Profiles (50) | Probes (3656) | OXD (55) | RB (40) | RC (41) | NOC |
|---|---|---|---|---|---|
| 1 | 38 | 0 | 0 | 0 | 0 |
| 2 | 453 | 0 | 0 | **13** | 0 |
| 3 | 501 | **1** | **31** | 0 | 1 |
| 4 | 466 | 0 | 0 | 0 | 0 |
| 5 | 96 | 0 | 0 | 0 | 0 |
| 6 | 715 | 0 | 0 | **28** | 0 |
| 7 | 841 | **54** | **9** | 0 | 9 |
| Total | 3110 | 55 | 40 | 41 | 136 |
| Missing | 14.93% | 0.00% | 0.00% | 0.00% | 0 |
| Probe Cluster Ability | | | | | 85.07% |
| Cluster Collisions | | | | | 7.35% |
| Sentinal Cluster Efficiency | | 98.18% | 77.50% | 68.29% | 81.32% |
| Sentinal Clustering Ability | | | | | 100.00% |

STEM v2

| Profiles (50) | Probes (9335) | OXD (61) | RB (40) | RC (45) | NOC |
|---|---|---|---|---|---|
| 1 | 77 | 0 | 0 | 0 | 0 |
| 2 | 7 | 0 | 0 | 0 | 0 |
| 3 | 6 | 0 | 0 | 0 | 0 |
| 4 | 21 | 0 | 0 | 0 | 0 |
| 5 | 14 | 0 | 0 | 0 | 0 |
| 6 | 27 | 0 | 0 | 0 | 0 |
| 7 | 11 | 0 | 0 | 0 | 0 |
| 8 | 47 | 0 | 0 | 0 | 0 |
| 9 | 89 | **3** | 0 | 0 | 0 |
| 10 | 4 | 0 | 0 | 0 | 0 |
| 11 | 305 | 0 | 0 | 0 | 0 |
| 12 | 18 | 0 | 0 | 0 | 0 |
| 13 | 133 | 0 | 0 | 0 | 0 |
| 14 | 57 | 0 | 0 | 0 | 0 |
| 15 | 300 | **4** | 0 | 0 | 0 |
| 16 | 662 | 0 | 0 | **22** | 0 |
| 17 | 10 | 0 | 0 | 0 | 0 |
| 18 | 1 | 0 | 0 | 0 | 0 |
| 19 | 64 | 0 | 0 | 0 | 0 |
| 20 | 14 | 0 | 0 | 0 | 0 |
| 21 | 272 | 0 | **36** | 0 | 0 |
| 22 | 14 | 0 | 0 | 0 | 0 |
| 23 | 83 | 0 | 0 | 0 | 0 |
| 24 | 20 | 0 | 0 | 0 | 0 |
| 25 | 389 | 0 | 0 | **20** | 0 |
| 26 | 0 | 0 | 0 | 0 | 0 |
| 27 | 60 | **1** | 0 | 0 | 0 |
| 28 | 60 | 0 | 0 | 0 | 0 |
| 29 | 22 | 0 | 0 | 0 | 0 |
| 30 | 105 | 0 | **2** | 0 | 0 |
| 31 | 24 | 0 | 0 | 0 | 0 |
| 32 | 55 | 0 | 0 | 0 | 0 |
| 33 | 51 | 0 | 0 | 0 | 0 |
| 34 | 61 | 0 | 0 | 0 | 0 |
| 35 | 32 | 0 | 0 | 0 | 0 |
| 36 | 75 | 0 | 0 | 0 | 0 |
| 37 | 389 | **3** | 0 | 0 | 0 |
| 38 | 6 | 0 | 0 | 0 | 0 |
| 39 | 468 | 0 | **2** | **1** | 1 |
| 40 | 11 | 0 | 0 | 0 | 0 |
| 41 | 10 | 0 | 0 | 0 | 0 |
| 42 | 31 | 0 | 0 | 0 | 0 |
| 43 | 35 | 2 | 0 | 0 | 0 |
| 44 | 39 | 0 | 0 | 0 | 0 |
| 45 | 7 | 0 | 0 | 0 | 0 |
| 46 | 74 | 0 | 0 | 0 | 0 |
| 47 | 109 | 0 | 0 | 0 | 0 |
| 48 | 57 | 0 | 0 | 0 | 0 |
| 49 | 676 | **44** | 0 | 0 | 0 |
| 50 | 0 | 0 | 0 | 0 | 0 |
| Total | 5102 | 57 | 40 | 43 | 140 |
| Missing | 45.35% | 6.56% | 0.00% | 4.44% | 6 |
| Probe Cluster Ability | | | | | 54.65% |
| Cluster Collisions | | | | | 0.71% |
| Sentinal Cluster Efficiency | | 77.19% | 90.00% | 51.16% | 72.79% |
| Sentinal Clustering Ability | | | | | 95.89% |

| Profiles (50) | Probes (3656) | OXD (55) | RB (40) | RC (41) | NOC |
|---|---|---|---|---|---|
| 1 | 72 | 0 | 0 | 0 | 0 |
| 2 | 100 | 0 | 0 | 0 | 0 |
| 3 | 9 | 0 | 0 | 0 | 0 |
| 4 | 5 | 0 | 0 | 0 | 0 |
| 5 | 0 | 0 | 0 | 0 | 0 |
| 6 | 0 | 0 | 0 | 0 | 0 |
| 7 | 11 | 0 | 0 | 0 | 0 |
| 8 | 1 | 0 | 0 | 0 | 0 |
| 9 | 23 | **1** | 0 | 0 | 0 |
| 10 | 13 | 0 | 0 | 0 | 0 |
| 11 | 4 | 0 | 0 | 0 | 0 |
| 12 | 323 | **1** | 0 | 0 | 0 |
| 13 | 559 | **51** | 0 | 0 | 0 |
| 14 | 66 | 0 | 0 | 0 | 0 |
| 15 | 19 | 0 | 0 | 0 | 0 |
| 16 | 2 | 0 | 0 | 0 | 0 |
| 17 | 10 | 0 | 0 | 0 | 0 |
| 18 | 4 | 0 | 0 | 0 | 0 |
| 19 | 5 | 0 | 0 | 0 | 0 |
| 20 | 11 | **1** | 0 | 0 | 0 |
| 21 | 9 | 0 | 0 | 0 | 0 |
| 22 | 1 | 0 | 0 | 0 | 0 |
| 23 | 6 | 0 | 0 | 0 | 0 |
| 24 | 13 | 0 | 0 | 0 | 0 |
| 25 | 0 | 0 | 0 | 0 | 0 |
| 26 | 20 | 0 | 0 | 0 | 0 |
| 27 | 11 | 0 | 0 | 0 | 0 |
| 28 | 58 | 0 | 0 | 0 | 0 |
| 29 | 157 | 0 | **6** | 0 | 0 |
| 30 | 562 | 0 | 0 | **36** | 0 |
| 31 | 1 | 0 | 0 | 0 | 0 |
| 32 | 4 | 0 | 0 | 0 | 0 |
| 33 | 5 | 0 | 0 | 0 | 0 |
| 34 | 324 | 0 | **30** | 0 | 0 |
| 35 | 300 | 0 | 0 | **4** | 0 |
| 36 | 2 | 0 | 0 | 0 | 0 |
| 37 | 24 | 0 | 0 | 0 | 0 |
| 38 | 8 | 0 | 0 | 0 | 0 |
| 39 | 120 | 0 | 0 | **1** | 0 |
| 40 | 6 | 0 | 0 | 0 | 0 |
| 41 | 13 | 0 | 0 | 0 | 0 |
| 42 | 0 | 0 | 0 | 0 | 0 |
| 43 | 13 | 0 | 0 | 0 | 0 |
| 44 | 36 | **1** | 0 | 0 | 0 |
| 45 | 16 | 0 | 0 | 0 | 0 |
| 46 | 106 | 0 | **3** | 0 | 0 |
| 47 | 31 | 0 | **1** | 0 | 0 |
| 48 | 7 | 0 | 0 | 0 | 0 |
| 49 | 12 | 0 | 0 | 0 | 0 |
| 50 | 0 | 0 | 0 | 0 | 0 |
| Total | 3102 | 55 | 40 | 41 | 136 |
| Missing | 15.15% | 0.00% | 0.00% | 0.00% | 0 |
| Probe Cluster Ability | | | | | 84.85% |
| Cluster Collisions | | | | | 0.00% |
| Sentinal Cluster Efficiency | | 92.73% | 75.00% | 87.80% | 85.18% |
| Sentinal Clustering Ability | | | | | 100.00% |

# t-SNE sklearn 3656

**E.**

(1) Two datasets of size 3656 probes were inputted into the t-SNE algorithm using the Python Scikit library. Clustering was done using k-means. The datasets were the original time series gene expression data provided by the probes and the other was the manifold data provided by AGCT from processing the original time series gene expression data. The results can be seen plotted in 2 and 3.

(2) Results plotted from t-SNE on original 3656 probe time series gene expression data.

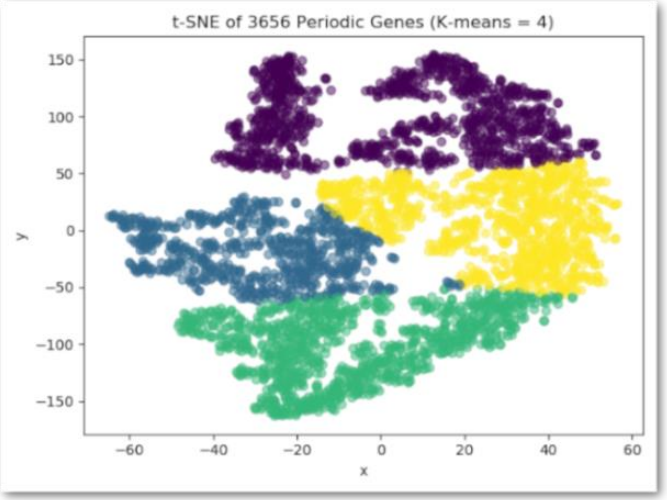


(3) Results plotted from t-SNE using manifold data provided AGCT after using 3656 probe time series gene expression data.

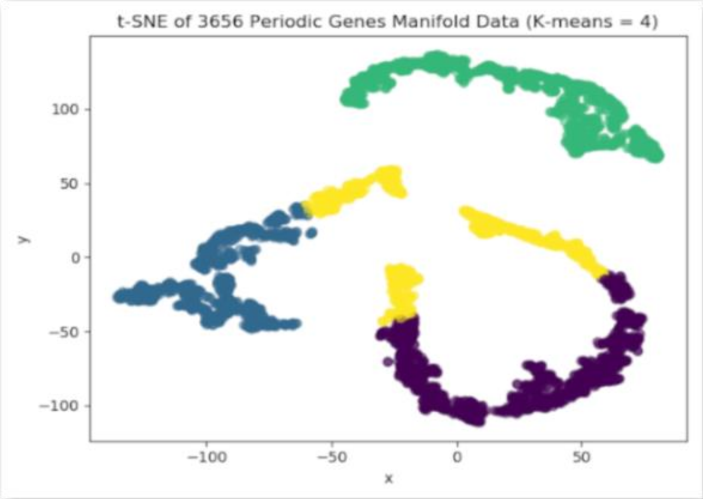


(4) The results of the clustering on the datasets can be seen below. The table shows number of Oxidative, Reactive Building and Reactive Charging genes in each cluster.

| Time Series Data | | | | | | Manifold Data | | | | | |
|---|---|---|---|---|---|---|---|---|---|---|---|
| Clusters (7) | Probes (3656) | OXD (55) | RB (40) | RC (41) | NOC | Clusters (7) | Probes (3656) | OXD (55) | RB (40) | RC (41) | NOC |
| 1 | 407 | **32** | 0 | **18** | 18 | 1 | 331 | 0 | 4 | 0 | 0 |
| 2 | 569 | 0 | **5** | **5** | 5 | 2 | 683 | **55** | 0 | 0 | 0 |
| 3 | 540 | 0 | 0 | **4** | 0 | 3 | 477 | 0 | 0 | **22** | 0 |
| 4 | 454 | 0 | **1** | **13** | 1 | 4 | 624 | 0 | 0 | 0 | 0 |
| 5 | 640 | **1** | **1** | **1** | 2 | 5 | 425 | 0 | 0 | **6** | 0 |
| 6 | 408 | **17** | **22** | 0 | 17 | 6 | 529 | 0 | 0 | **13** | 0 |
| 7 | 638 | **5** | **11** | 0 | 5 | 7 | 587 | 0 | **36** | 0 | 0 |
| Total | 3656 | 55 | 40 | 41 | 136 | Total | 3656 | 55 | 40 | 41 | 136 |
| Missing | 0.00% | 0.00% | 0.00% | 0.00% | 0 | Missing | 0.00% | 0.00% | 0.00% | 0.00% | 0 |
| Probe Cluster Ability | | | | | 100.00% | Probe Cluster Ability | | | | | 100.00% |
| Cluster Collisions | | | | | 35.29% | Cluster Collisions | | | | | 0.00% |
| Sentinal Cluster Efficiency | | 58.18% | 55.00% | 43.90% | 52.36% | Sentinal Cluster Efficiency | | 100.00% | 90.00% | 53.66% | 81.22% |
| Sentinal Clustering Ability | | | | | 100.00% | Sentinal Clustering Ability | | | | | 100.00% |

# t-SNE sklearn 9335

**F.**

(1) Two datasets of size 9335 probes were inputted into the t-SNE algorithm using the Python Scikit library. The k-means algorithm was used for clustering. The datasets were the original time series gene expression data provided by the probes and the other was the manifold data provided by AGCT from processing the original time series gene expression data. The results can be seen plotted in 2 and 3.

(2) Results plotted from t-SNE on original 9335 probe time series gene expression data.

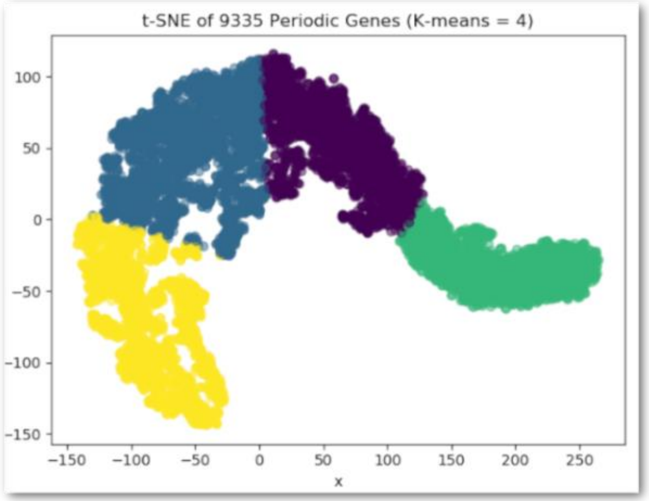


(3) Results plotted from t-SNE using manifold data provided AGCT after using 9335 probe time series gene expression data.

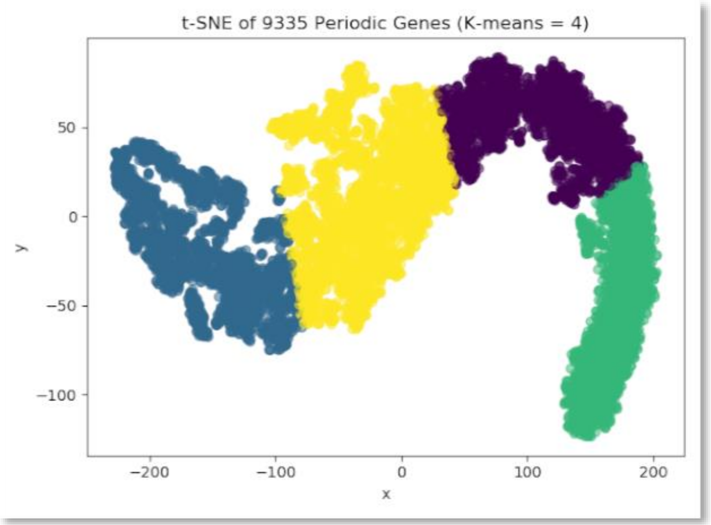


(4) The results of the clustering on the datasets is below. The table shows number of Oxidative, Reactive Building and Reactive Charging genes in each cluster.

| Time Series Data | | | | | | Manifold Data | | | | | |
|---|---|---|---|---|---|---|---|---|---|---|---|
| Clusters (3) | Probes (9335) | OXD (61) | RB (40) | RC (45) | NOC | Clusters (3) | Probes (9335) | OXD (61) | RB (40) | RC (45) | NOC |
| 1 | 3550 | **3** | **6** | **8** | 9 | 1 | 2657 | **3** | **6** | **8** | 9 |
| 2 | 2720 | **4** | 0 | **1** | 1 | 2 | 3140 | **4** | 0 | **1** | 1 |
| 3 | 3065 | **54** | **34** | **36** | 70 | 3 | 3538 | **54** | **34** | **36** | 70 |
| Total | 9335 | 61 | 40 | 45 | 146 | Total | 9335 | 61 | 40 | 45 | 146 |
| Missing | 0.00% | 0.00% | 0.00% | 0.00% | 0 | Missing | 0.00% | 0.00% | 0.00% | 0.00% | 0 |
| Probe Cluster Ability | | | | | 100.00% | Probe Cluster Ability | | | | | 100.00% |
| Cluster Collisions | | | | | 54.79% | Cluster Collisions | | | | | 54.79% |
| Sentinal Cluster Efficiency | | 88.52% | 85.00% | 80.00% | 84.51% | Sentinal Cluster Efficiency | | 88.52% | 85.00% | 80.00% | 84.51% |
| Sentinal Clustering Ability | | | | | 100.00% | Sentinal Clustering Ability | | | | | 100.00% |

# Isomap scikit 3656

**G.**

(1) Two datasets of 3656 probes were inputted into the t-SNE algorithm from Python's Scikit library. The datasets were the original time-series gene expression data, and the manifold data provided by AGCT from processing the original time-series gene expression data. The results can be seen plotted in 2 and 4.

(3) Results plotted from Isomap on original 9335 probes time-series gene expression data.

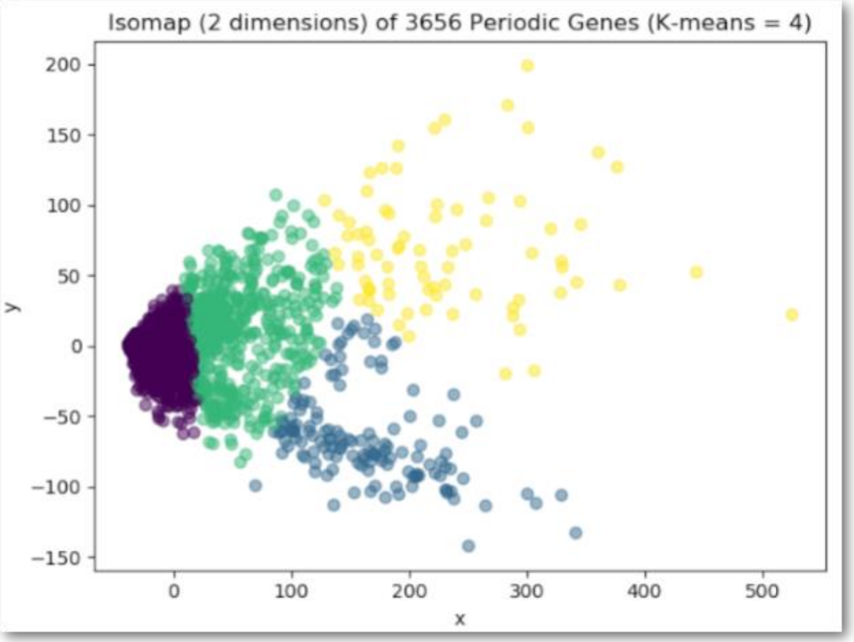


(4) Results plotted from Isomap on original 3656 probes using the manifold data.

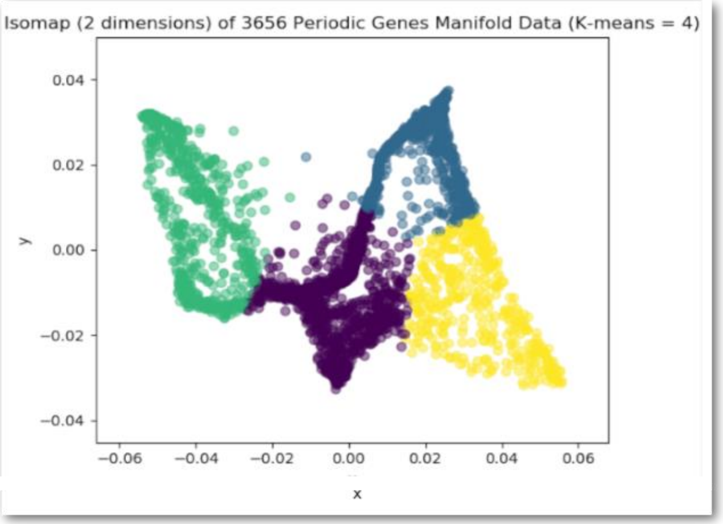


(5) The results from clustering on the dataset can be seen below. The table shows the number of Oxidative, Reactive Building and Reactive Charging genes in each cluster.

| Time Series Data | | | | | |
|---|---|---|---|---|---|
| Clusters (7) | Probes (3656) | OXD (55) | RB (40) | RC (41) | NOC |
| 1 | 2239 | 5 | 18 | 9 | 14 |
| 2 | 128 | 0 | 0 | 7 | 0 |
| 3 | 253 | 0 | 0 | 16 | 0 |
| 4 | 79 | 16 | 0 | 0 | 0 |
| 5 | 43 | 0 | 0 | 4 | 0 |
| 6 | 157 | 24 | 0 | 0 | 0 |
| 7 | 757 | 10 | 22 | 5 | 15 |
| Total | 3656 | 55 | 40 | 41 | 136 |
| Missing | 60.84% | 0.00% | 0.00% | 0.00% | 0 |
| Probe Cluster Ability | | | | | 100.00% |
| Cluster Collisions | | | | | 21.32% |
| Sentinal Cluster Efficiency | | 43.64% | 55.00% | 39.02% | 45.89% |
| Sentinal Clustering Ability | | | | | 93.15% |

| Manifold Data | | | | | |
|---|---|---|---|---|---|
| Clusters (7) | Probes (3656) | OXD (55) | RB (40) | RC (41) | NOC |
| 1 | 297 | 0 | 2 | 5 | 2 |
| 2 | 561 | 8 | 6 | 7 | 13 |
| 3 | 564 | 11 | 7 | 11 | 18 |
| 4 | 335 | 9 | 5 | 2 | 7 |
| 5 | 404 | 5 | 5 | 3 | 8 |
| 6 | 1340 | 20 | 14 | 11 | 25 |
| 7 | 155 | 2 | 1 | 2 | 3 |
| Total | 3656 | 55 | 40 | 41 | 136 |
| Missing | 0.00% | 0.00% | 0.00% | 0.00% | 0 |
| Probe Cluster Ability | | | | | 100.00% |
| Cluster Collisions | | | | | 55.88% |
| Sentinal Cluster Efficiency | | 36.36% | 35.00% | 26.83% | 32.73% |
| Sentinal Clustering Ability | | | | | 100.00% |

# Visgenex 2.0

**H.**

(1) One dataset of 3656 time-series gene expressions was run using Visgenex. No-Fscoring was used. The process and result can be seen in 2, 3, and 4. The results of the clustering on the dataset can be seen in 5. The table shows the number of Oxidative, Reactive Building and Reactive Charging genes in each cluster.

(5) Visgenex 2.0 clustering results.

| Clusters (4) | Probes (3656) | OXD (55) | RB (40) | RC (41) | NOC |
|---|---|---|---|---|---|
| 1 | 925 | 0 | **40** | 0 | 0 |
| 2 | 944 | 0 | 0 | **16** | 0 |
| 3 | 1004 | **55** | 0 | 0 | 0 |
| 4 | 783 | 0 | 0 | **25** | 0 |
| Total | 3656 | 55 | 40 | 41 | 136 |
| Missing | 0.00% | 0.00% | 0.00% | 0.00% | 0 |
| Probe Cluster Ability | | | | | 100.00% |
| Cluster Collisions | | | | | 0.00% |
| Sentinal Cluster Efficiency | | 100.00% | 100.00% | 60.98% | 86.99% |
| Sentinal Clustering Ability | | | | | 100.00% |

(2) Visgenex 2.0 gui repository.

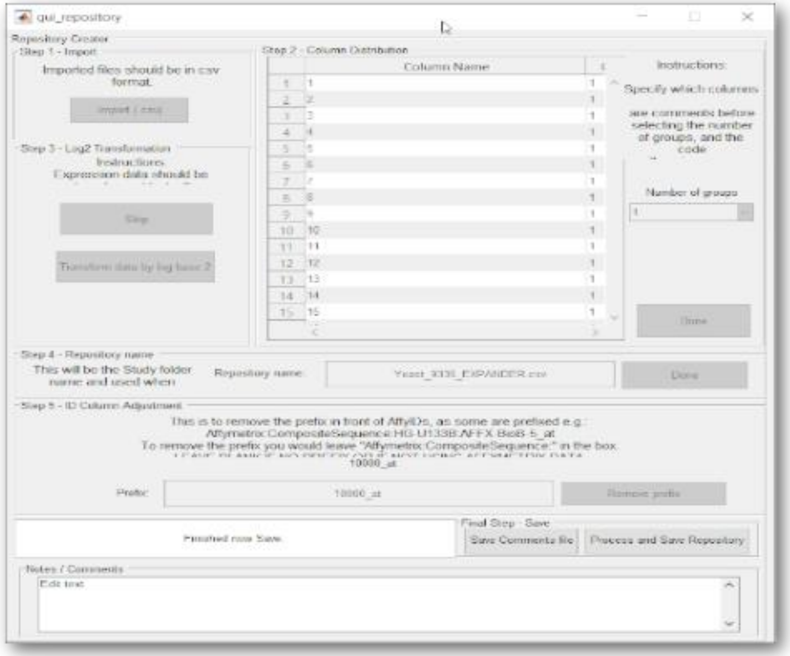

(3) Visgenex 2.0 gui study.

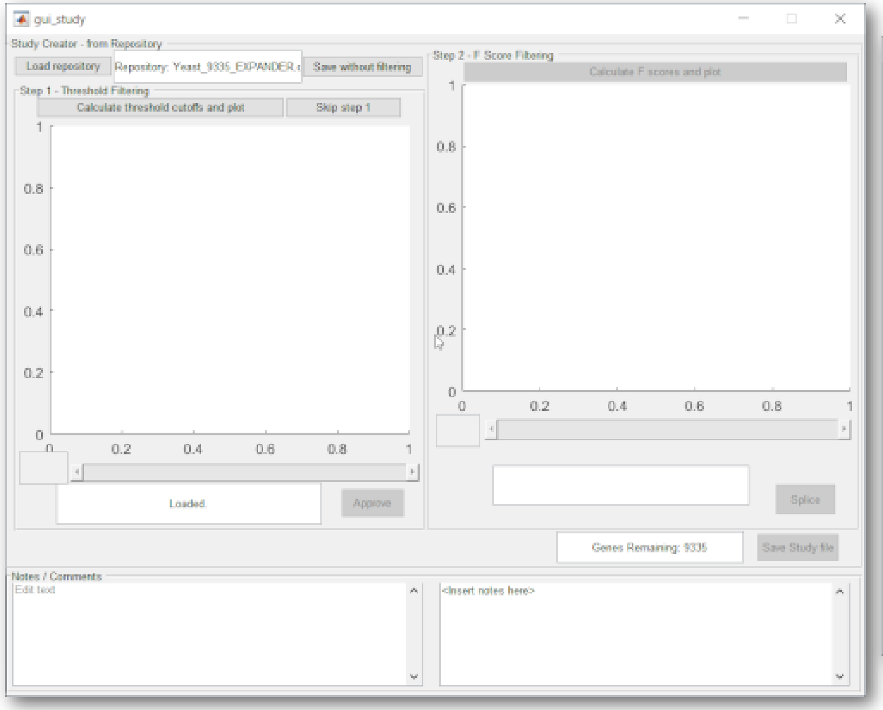

(4) Visgenex 2.0 analysis tools.

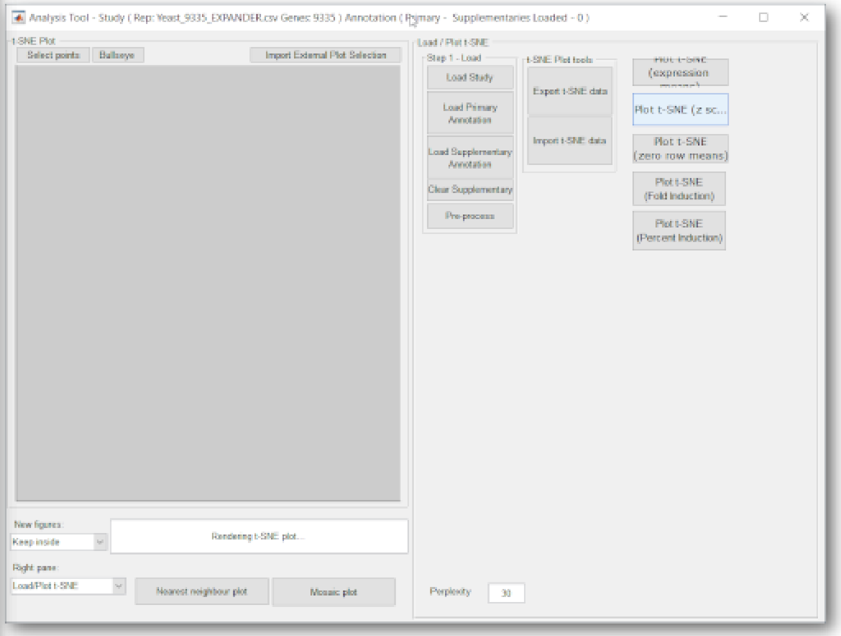

Supplement: S8 Fig — (PDF) [file pone.0233755.s017.pdf]
